# Clonal evolution in primary breast cancers under sequential epirubicin and docetaxel monotherapy

**DOI:** 10.1186/s13073-022-01090-2

**Published:** 2022-08-11

**Authors:** Andreas Venizelos, Christina Engebrethsen, Wei Deng, Jürgen Geisler, Stephanie Geisler, Gjertrud T. Iversen, Turid Aas, Hildegunn S. Aase, Manouchehr Seyedzadeh, Eli Sihn Steinskog, Ola Myklebost, Sigve Nakken, Daniel Vodak, Eivind Hovig, Leonardo A. Meza-Zepeda, Per E. Lønning, Stian Knappskog, Hans P. Eikesdal

**Affiliations:** 1 K.G.Jebsen Center for Genome Directed Cancer Therapy, Department of Clinical Science, University of Bergen, Bergen, Norway; 2 Department of Oncology, Haukeland University Hospital, Bergen, Norway; 3 Current address: Department of Oncology, Akershus University Hospital, Lørenskog, Norway; 4 Department of Surgery, Haukeland University Hospital, Bergen, Norway; 5 Department of Radiology, Haukeland University Hospital, Bergen, Norway; 6 Current address: Department of Radiology, Akershus University Hospital, Lørenskog, Norway; 7 Department of Tumor Biology, Institute of Cancer Research, The Norwegian Radium Hospital, Oslo University Hospital, Oslo, Norway; 8 Norwegian Cancer Genomics Consortium, Institute for Cancer Research, The Norwegian Radium Hospital, Oslo University Hospital, Oslo, Norway; 9 Current address: Department of Clinical Science, University of Bergen, Bergen, Norway; 10 Centre for Cancer Cell Reprogramming, Institute of Clinical Medicine, Faculty of Medicine, University of Oslo, Oslo, Norway; 11 Centre for Bioinformatics, Department of Informatics, University of Oslo, Oslo, Norway; 12 Genomics Core Facility, Department of Core Facilities, Institute of Cancer Research, The Norwegian Radium Hospital, Oslo University Hospital, Oslo, Norway

**Keywords:** Breast cancer, Clonal evolution, Neoadjuvant chemotherapy, Chemoresistance

## Abstract

**Background:**

Subclonal evolution during primary breast cancer treatment is largely unexplored. We aimed to assess the dynamic changes in subclonal composition of treatment-naïve breast cancers during neoadjuvant chemotherapy.

**Methods:**

We performed whole exome sequencing of tumor biopsies collected before, at therapy switch, and after treatment with sequential epirubicin and docetaxel monotherapy in 51 out of 109 patients with primary breast cancer, who were included in a prospectively registered, neoadjuvant single-arm phase II trial.

**Results:**

There was a profound and differential redistribution of subclones during epirubicin and docetaxel treatment, regardless of therapy response. While truncal mutations and main subclones persisted, smaller subclones frequently appeared or disappeared. Reassessment of raw data, beyond formal mutation calling, indicated that the majority of subclones seemingly appearing during treatment were in fact present in pretreatment breast cancers, below conventional detection limits. Likewise, subclones which seemingly disappeared were still present, below detection limits, in most cases where tumor tissue remained. Tumor mutational burden (TMB) dropped during neoadjuvant therapy, and copy number analysis demonstrated specific genomic regions to be systematically lost or gained for each of the two chemotherapeutics.

**Conclusions:**

Sequential epirubicin and docetaxel monotherapy caused profound redistribution of smaller subclones in primary breast cancer, while early truncal mutations and major subclones generally persisted through treatment.

**Trial registration:**

ClinicalTrials.gov, NCT00496795, registered on July 4, 2007.

**Supplementary Information:**

The online version contains supplementary material available at 10.1186/s13073-022-01090-2.

## Background

The genomic landscape of early as well as metastatic breast cancer has been described in detail [1–6]. While subclonal redistribution from the primary to the metastatic setting is frequently observed [6, 7], it is challenging to distinguish redistributions caused by the metastatic process and immunoediting [8] from those caused by therapy-induced selection [9–11].

A better understanding of subclonal redistribution and identification of emerging or disappearing genomic aberrations during treatment would pave the way for identification of molecular mechanisms underlying resistance to the treatment applied and pinpoint potential targets to improve therapeutic outcome. Neoadjuvant trials present an ideal setting to explore molecular aberrations dictating response to chemotherapy, as well as subclonal redistribution, in early breast cancer. Here, therapy response may be recorded in detail clinically, or radiologically using magnetic resonance imaging (MRI). Moreover, for patients with larger tumors, repeated biopsies can be collected before and after treatment with each particular regimen [12–14].

Anthracyclines and taxanes are the two types of chemotherapy most frequently applied in breast cancer. The lack of cross-resistance between these compounds provides a rationale for sequential monotherapy instead of combination regimens, thereby allowing the application of higher doses, including dose-dense treatment, for each compound [15–17]. Importantly, a recent meta-analysis demonstrated sequential therapy to be superior to or at least as good as combined treatment [15]. Moreover, a monotherapy design allows identification of factors predicting outcome to individual compounds [18], contrasting combination regimens for which factors predicting sensitivity to individual compounds may not be identified.

In order to explore the efficacy of sequential dose-dense epirubicin and docetaxel monotherapy and to identify potential predictive biomarkers to guide treatment selection, we conducted a phase II clinical trial in 109 patients with large, treatment-naïve primary breast cancers. We performed whole exome sequencing (WES) of biopsies collected before and after epirubicin and docetaxel treatment in a subset of patients (*n* = 51) selected to balance the number of responders and non-responders to each regimen (see the “Methods” section). Furthermore, out of the remaining tumors (*n* = 58), 45 pretreatment biopsies underwent targeted sequencing of six key breast cancer-related genes. While we detected no specific genomic alteration predicting response to therapy, we recorded a profound redistribution of cancer subclones during treatment with both drugs. Notably, for most of the investigated tumors, subclones appearing or disappearing could be identified as small subclones below conventional detection limits before and after treatment, respectively.

## Methods

### Patients and study protocol

The Dose-Dense Protocol (DDP) study was a prospectively registered, single-arm, phase II clinical trial which enrolled patients ≤ 65 years, with non-inflammatory, primary breast cancer and tumor size > 4 cm and/or N2-3 lymph node metastases. Patients were enrolled on an all-comers basis, provided eligibility according to the inclusion criteria and upon each patient’s informed consent. Enrollment was performed at a single institution in Bergen, Norway (for details, see Additional file 1). The trial was approved by the Regional Ethics Committee of Western Norway and registered at ClinicalTrials.org (NCT00496795) prior to trial initiation. The first patient was included on November 19, 2007, and the last patient entered the trial on February 11, 2016. Patient inclusion has been finalized, but survival follow-up continues for 10 years postoperatively (i.e., until February 11, 2026).

**Fig. 1:**
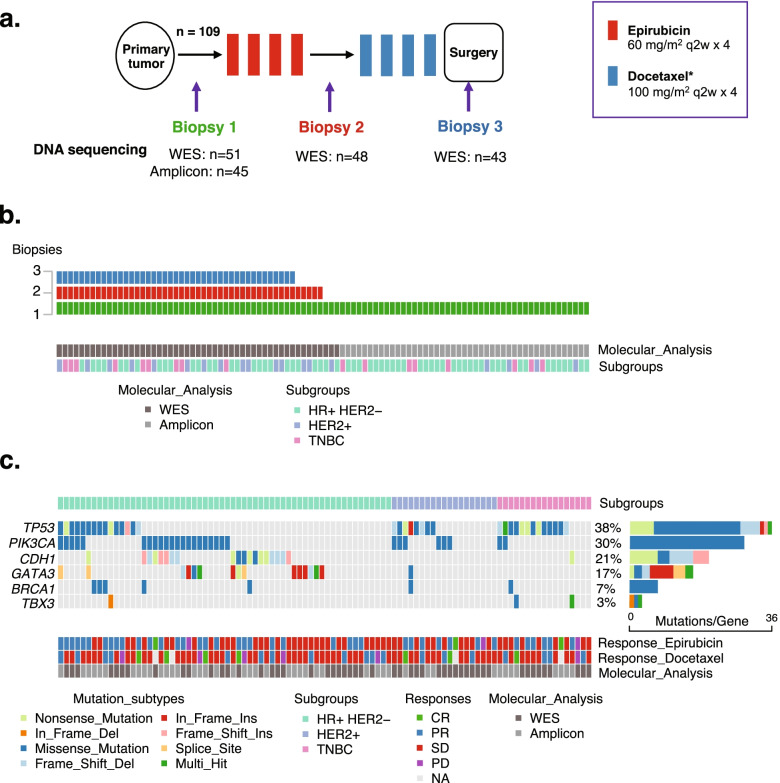
**a** Dose-Dense trial design. All patients (*n* = 109) received sequential epirubicin and docetaxel monotherapy*. Biopsies were collected before treatment, after epirubicin, and after docetaxel. Whole exome sequencing (WES) was performed for 51 patients in pretreatment tumor biopsies and matched blood samples. Among these 51 patients, repeated tumor biopsies after epirubicin (*n* = 48) and after docetaxel (*n* = 43) were subjected to WES (all 43 patients with biopsies after docetaxel were among the 48 with biopsies after epirubicin). Out of the remaining 58 patients in the trial, pretreatment tumor biopsies from 45 underwent amplicon-based targeted sequencing of a six-gene panel. **b** Diagram depicting the number of tumor biopsies used for DNA sequencing pretreatment (green), after epirubicin (red), and after docetaxel (blue). Out of 96 pretreatment biopsies, WES was performed on 51 (dark gray) and amplicon-based sequencing on the remaining 45 samples (light gray), whereas subsequent analyses after epirubicin (*n* = 48) and docetaxel treatment (*n* = 43) were performed by WES only. The distribution of breast cancer subgroups (hormone receptor positive, HER2 normal (HR+HER2−), HER2 positive (HER2+), and triple-negative breast cancers (TNBC)) are indicated by light green, purple, and pink bars, respectively, in the lower panel. **c** Mutation status pretreatment. Oncoplot showing mutations of six genes in pretreatment samples from all patients in the study with available tumor DNA (*n* = 96). The mutation list is sorted by the subgroups; HR+/HER2−, HER2+, and TNBC. Mutations are colored according to mutation type. Percentages and bars on the right indicate the prevalence of mutations in each of the genes, among the 96 tumor samples analyzed. Each column represents one tumor/patient. Colors in the lower panel show the individual clinical responses to sequential epirubicin and docetaxel* neoadjuvant chemotherapy, and the molecular analysis used for each tumor sample (whole exome sequencing (WES) or amplicon-based sequencing). Responses listed: CR (complete response), PR (partial response), SD (stable disease), according to RECIST, and PD (progressive disease), according to UICC criteria. *HER2-positive breast cancers received concomitant docetaxel and trastuzumab

The study protocol (Additional file 2) dictated four courses of i.v. epirubicin 60 mg/m^2^ q2w followed by four courses of docetaxel 100 mg/m^2^ q2w (Fig. 1a) prior to surgery, including pegfilgrastim after each chemotherapy course. Patients who did not tolerate docetaxel treatment were shifted to paclitaxel (*n* = 3), 80 mg/m^2^ qW for the remaining part of the 8-week taxane period. Patients with HER2-positive disease received weekly i.v. trastuzumab (4 mg/kg loading dose, then maintenance 2 mg/kg) during neoadjuvant taxane treatment, as well as postoperatively, to a total treatment duration of 52 weeks. All patients received postoperative radiotherapy to the chest wall and regional lymph nodes, and adjuvant endocrine therapy (premenopausal women: tamoxifen, postmenopausal women: aromatase inhibitor (AI) or sequential AI-tamoxifen) was given for 5 years to patients with hormone receptor (HR)-positive disease, in accordance with national guidelines.

All tissue samples in the present study were collected as dictated by the DDP trial protocol. Prior to commencing neoadjuvant treatment, all patients had an incisional breast tumor biopsy taken. Additionally, a Tru-cut biopsy was obtained, from the same site, after completing epirubicin treatment, and finally, tumor tissue, again from the same site, was sampled at surgery (Fig. 1a). All samples were snap-frozen in liquid nitrogen immediately upon removal. Leucocytes from pretreatment blood samples were used as a source for normal DNA.

Clinical and MRI responses to epirubicin and subsequent docetaxel treatment were classified according to the Response Evaluation Criteria in Solid Tumors (RECIST) [19], where patients were dichotomized into responders (complete response (CR) and partial response (PR), i.e., objective response) and non-responders (stable disease (SD) and progressive disease (PD)). Additionally, to assure early detection of progressive disease, the common UICC criteria were used, where PD was defined as an increase of ≥25% in the product of the largest and the perpendicular tumor diameter [20], as opposed to ≥20% increase in the largest tumor diameter by the RECIST criteria (see Additional file 1 for details). Follow-up was defined as the time period from trial inclusion until data cut-off date or death for each patient. No patient was lost to follow-up.

The trial’s primary endpoint was to correlate molecular parameters to objective response to each of the two chemotherapy regimens applied. The secondary endpoints were (a) to correlate molecular parameters to relapse-free and overall survival and (b) to identify and explore characteristics of epithelial and mesenchymal stem cells isolated in tumor tissue and bone marrow. Apart from a previous case report of a patient with lethal pneumonitis [21], this is the first report of the primary and secondary endpoints of the trial. The last survival assessment was conducted in March 2021, when each patient had a minimum of 5 years of follow-up from inclusion in the trial or until the time of death. The secondary endpoint (b) regarding stem cell assessment is not addressed in the current report.

### Massive parallel sequencing

The present analyses were performed on preoperative tumor biopsies from 109 patients included in the DDP trial (Fig. 1a, b; for details, see Additional file 1 and Additional file 3: Fig. S1). Among these patients, response outliers (*n* = 51/109) were selected for whole exome sequencing (WES), based on objective response (OR) or lack of OR to epirubicin and/or docetaxel and availability of tumor tissue for DNA extraction, with a minimum of eight patients in each group (Table 1). Tumor DNA samples from eight patients with OR to both epirubicin and docetaxel, 18 patients without OR to either drug, and 25 patients with OR to one drug only were analyzed. Within this selection, 11 patients had a pathological complete response (pCR) (Table 1).

**Table 1:** Tumors selected for DNA sequencing strategies, based on clinical response^a^

**WES**^**b**^ **(*n*= 51)**		***Docetaxel responder***		***Docetaxel non-responder***		***Docetaxel not evaluable***
		CR	PR	SD	PD	
***Epirubicin responder***	CR					**2** (1)
	PR	**2** (1)	**6** (3)	**13** (4)	**2** (0)	
***Epirubicin non-responder***	SD		**8** (1)	**15** (0)		
	PD			**2** (1)	**1** (0)	
**Amplicon**^**c**^ **(*n*= 45)**		***Docetaxel responder***		***Docetaxel non-responder***		***Docetaxel not evaluable***
		CR	PR	SD	PD	
***Epirubicin responder***	CR					**1** (0)
	PR	**4** (0)	**2** (0)	**9** (1)	**2** (1)	
***Epirubicin non-responder***	SD	**1** (0)	**3** (1)	**21** (0)	**1** (0)	**1** (0)
	PD					

Numbers in paratheses indicate patients with pCR at surgery

^a^Tumors selected as response outliers (response per RECIST) and with tumor tissue available for DNA extraction (*n* = 96/109)

^b^WES whole exome sequencing

^c^Amplicon: amplicon-based sequencing of 6-gene panel

For the purpose of longitudinal comparisons (assessment of subclonal evolution), WES was performed as described previously [22], using DNA from tumor biopsies collected pretreatment, after epirubicin, and after docetaxel, together with matched blood DNA. Among the 51 response outliers, WES was performed on DNA from 51 pretreatment biopsies, 48 biopsies collected after epirubicin (but before docetaxel), and 43 biopsies collected after sequential epirubicin and docetaxel, as well as on matched pretreatment blood samples (*n* = 51). Eleven post-treatment biopsies (three after epirubicin, eight after docetaxel) were not sequenced due to the lack of tumor DNA (Fig. 1a, b). In brief, library preparation was performed using the Agilent SureSelectXT Human All Exon V5 kit, and the resulting library was sequenced on a HiSeq2500 (Illumina Inc, San Diego, CA, USA).

For the remaining patients (*n* = 58/109), tissue for genomic analysis was available from 45 pretreatment tumor biopsies (Fig. 1a, b). To assess the potential predictive value of mutations in key breast cancer genes, pretreatment samples from these tumors were analyzed for mutations in *BRCA1*, *PIK3CA*, *TP53*, *CDH1*, *GATA3*, and *TBX3* by amplicon-based targeted sequencing and the results were combined with pretreatment status of the same genes in the WES-analyzed tumors. The five former genes were selected on the basis of being key breast cancer genes, while *TBX3* was selected due its potential role as a *CDH1* regulator and due to its relatively high mutation frequency in breast cancer. Library preparation was performed with a custom-designed Accel-Amplicon panel (Swift Biosciences, Ann Arbor, MI, USA) and sequencing was performed on an illumina MiSeq (for details, see Additional file 1).

### Data analyses

#### Mutation calling

Sequence reads were aligned to the human genome (Build-UCSC hg19) using the BWA-MEM alignment algorithm [23]. Sample-wise sorting and duplicate marking were performed using Picard tools (http://broadinstitute.github.io/picard). Indel realignment and base quality recalibration were performed by GATK tools [24]. Somatic small variant identification on the matched tumor-normal sample pairs was performed using MuTect [25] (single nucleotide variant [SNV] detection) and Strelka [26] (SNV and small indel detection), using default parameters, and applying the intersect as the true positive for SNVs [22]. Variant allelic fraction (VAF) of at least 5% was required for further analyses. Functional annotation of SNVs and InDels was performed with ANNOVAR [27], and only amino acid changing mutations were included for further analyses.

#### Copy number analysis

Copy number profiling and assessment of tumor cell fraction were performed using the ASCAT algorithm [28].

#### Mutational signatures

Mutational signatures were determined using the R package DeconstructSigs [29, 30]. Quality control of the raw input data was performed with the FastQC program (http://www.bioinformatics.babraham.ac.uk/projects/fastqc).

#### Annotation of driver events

To identify likely driver events, we followed a similar approach as we have previously described for breast cancer data sets [3]. We used a two-step approach where we first selected the genes most likely to contribute to breast cancer oncogenesis and then, for each individual mutation within these genes, assessed any evidence indicating a role as a driver mutation (for details, see Additional file 1).

#### Estimation of subclonality

For a measure of the cellular prevalence of mutations, we calculated rVAF of each mutation as the ratio between observed and expected VAF, given local copy number state, tumor cell fraction, and estimated number of mutated alleles.

$$rVAF=VAFobs/VAFexp=VAFobs/(n mut x p/2x(1-p)+n tot x p),$$

where *n*mut refers to the number of mutated alleles, *n*tot refers to the total copy number at the mutated locus, and *ρ* refers to the tumor cell fraction. Sample mutation clustering across samples collected at different time points for each patient (pretreatment, post-epirubicin, and post-docetaxel/after neoadjuvant treatment) was performed by the use of PyClone [31] and displayed in parallel coordinate and fish plots (Additional file 3: Fig. S9).

#### Graphics

All graphics were generated using R version 3.6.1 (http://www.R-project.org/). The "ggplot2()" function was used to generate coxcomb plots [32]. Other packages used were dplyr, data.table, and tidyverse. Time scape and copy number packages from bioconductor were used for the visualization of clonal evolution and copy number alterations, respectively [33, 34].

Full details about the data analyses are given in Additional file 1.

### Statistical analyses

Correlations between mutations and response to therapy were assessed by the Fisher exact test and trend test across the response groups (prop.trend.test function in R). Comparison of continuous variables between groups was performed by the Mann-Whitney rank test, while the Wilcoxon rank test was used for paired samples. Survival data were assessed by a Cox regression analysis, calculating hazard ratios for each parameter. For Kaplan-Meier plots, patient subgroups were compared by the log rank test (for further details, see Additional file 1). Genomic Identifications of Significant Targets in Cancer (GISTIC) 2.0 [35] was used to identify frequent focal- and arm-level amplifications and deletions.

Statistical analyses were performed using R, version 3.6.3, or the SPSS 26/PASW 17.0 software package. All *p*-values reported are two-tailed, and *p* < 0.05 was considered statistically significant.

## Results

### Patient characteristics and clinical outcome

Out of 109 patients included in the trial, 97% (*n* = 106/109) completed epirubicin, whereas 88% (*n* = 95/108) completed docetaxel treatment per protocol (CONSORT diagram; Additional file 3: Fig. S1). Patient and tumor baseline characteristics are given in Additional file 4: Table S1 and Additional file 1. The median largest tumor diameter at inclusion was 57 mm (range 12–270 mm). Tumor responses to sequential epirubicin and docetaxel are summarized in Additional file 4: Table S3. Briefly, the clinical objective response rate (ORR) after completing sequential epirubicin and docetaxel was 71.6%, where initial epirubicin treatment yielded an ORR of 41.3%, followed by ORR 29.5% for docetaxel after epirubicin. In comparison, for breast MRI, the ORR was 91.7%, with ORR 36.2% for epirubicin and 76.8% for docetaxel, excluding patients where MRI exams were not performed as part of the evaluation of treatment response. Overall, the pCR rate was 15% (*n* = 16/107), with a pCR of 3% for HR positive, HER2−; 33% for HER2+ breast cancers; and 30% for triple-negative breast cancers (TNBC) (Additional file 4: Table S3). For further details regarding clinical results of the trial, see Additional file 1.

Each patient had a minimum of 5 years of follow-up from inclusion in the trial (median 111 months; range 61–160 months), or until the time of death. The survival outcome is depicted in Additional file 3 (Fig. S2). For patients with M0 disease at inclusion and who underwent surgery, recurrences have been established in 25 out of 106 patients (24%). The median disease-free survival was 95 months (range 1–156). Out of 20 patients (19%) who died during follow-up, 16 patients died due to breast cancer, one died due to the breast cancer treatment (see below), and three died from other causes, unrelated to breast cancer or breast cancer treatment. The median overall survival was 103 months (range 5–160).

Adverse events were retrospectively collected by review of the patients’ hospital records and scored using Common Terminology Criteria for Adverse Events (CTCAE), version 4. Apart from one patient dying from a taxane-induced pneumonitis [21], the side effects from the neoadjuvant chemotherapy was as expected, with hand-foot syndrome, peripheral neuropathy, and infectious complications being the most common grade 2–4 adverse events (for details, see Additional file 1 and Additional file 4: Table S2).

### Massive parallel sequencing

The current molecular analyses are based on biopsies from 109 patients included in the DDP trial (Fig. 1b; for details, see Table 1 and in Additional file 5: Table S4). For longitudinal assessment of subclonal composition, WES was performed on tumor biopsies from 51 response outliers: pretreatment biopsies (*n* = 51), post-epirubicin but pre-docetaxel biopsies (*n* = 48), and post-docetaxel biopsies (*n* = 43), as well as on matched blood samples (*n* = 51). Eleven post-treatment biopsies (three after epirubicin, eight after docetaxel) were not sequenced due to the lack of tumor DNA (Fig. 1a, b). The mean of mean target coverage for WES was 159× for tumor samples and 69× for blood samples (Additional file 3: Fig. S3).

Out of the remaining 58 patients, 45 pretreatment biopsies had sufficient DNA for analyses. To assess the predictive impact of mutations in key breast cancer genes, these samples were subject to amplicon-based targeted sequencing of a six-gene panel, resulting in a mean target coverage of 5094× for tumor samples (matched blood samples not analyzed; Additional file 3: Fig. S4).

#### Mutational spectrum in pretreatment samples

Six predefined, key breast cancer genes were assessed in all 96 pretreatment tumor samples, either by WES or by amplicon sequencing, based on their high mutational prevalence and/or predominant role in breast cancer progression [2, 36, 37]. This analysis yielded mutation frequencies of *TP53* 38%, *PIK3CA* 30%, *CDH1* 21%, *GATA3* 17%, *BRCA1* 7%, and *TBX3* 3% (Fig. 1c). Thus, the frequencies of *CDH1* and *BRCA1* mutations were slightly higher than in larger breast cancer cohorts [38], probably due to random variation within our sample set. Mutations in none of these selected key breast cancer genes were predictive of response to either epirubicin or subsequent docetaxel treatment (Additional file 4: Table S5).

In the 51 tumors subjected to WES, in addition to mutations in the genes described above, six tumors harbored *PTEN* mutations (12%), and five tumors harbored *FLG* and *MUC16* mutations (each 10%). The mutational distribution across individual genes is depicted in Additional file 3: Fig. S5. When testing for predictive value across genes mutated in >2 patients, *JAK2* mutations (*n* = 3/51) were associated with response to epirubicin treatment (*p* = 0.02); however, this did not remain significant after correction for multiple testing (Additional file 4: Table S6). No other mutations or sets of mutations in key breast cancer pathways examined by WES in pretreatment biopsies predicted response to epirubicin.

Regarding copy number alterations (CNAs), pretreatment samples revealed a high frequency of losses within 1p, 8p, 11q, 17p, and 19p and gains within 1q and 8q (Additional file 3: Fig. S6). Based on copy number profiling, we inferred whole-genome duplication (WGD) events to have occurred in 18 out of 51 tumors (35%).

The tumor mutational burden (TMB) in pretreatment biopsies analyzed by WES varied from 0.02 to 8.38 mutations per megabase (MB) (median *n* = 0.64, mean *n* = 1.14). While the mean TMB in pretreatment biopsies was numerically lower in responders than non-responders (TMB *n* = 0.94 versus *n* = 1.32), this was not statistically significant. We found no correlation between CNAs and TMB (Additional file 3: Fig. S7).

Analyzing for somatic mutation signatures previously defined in breast cancer [39], no signature was predictive of response to epirubicin (Additional file 3: Fig. S8a).

### Mutational spectrum after epirubicin treatment

**Fig. 2:**
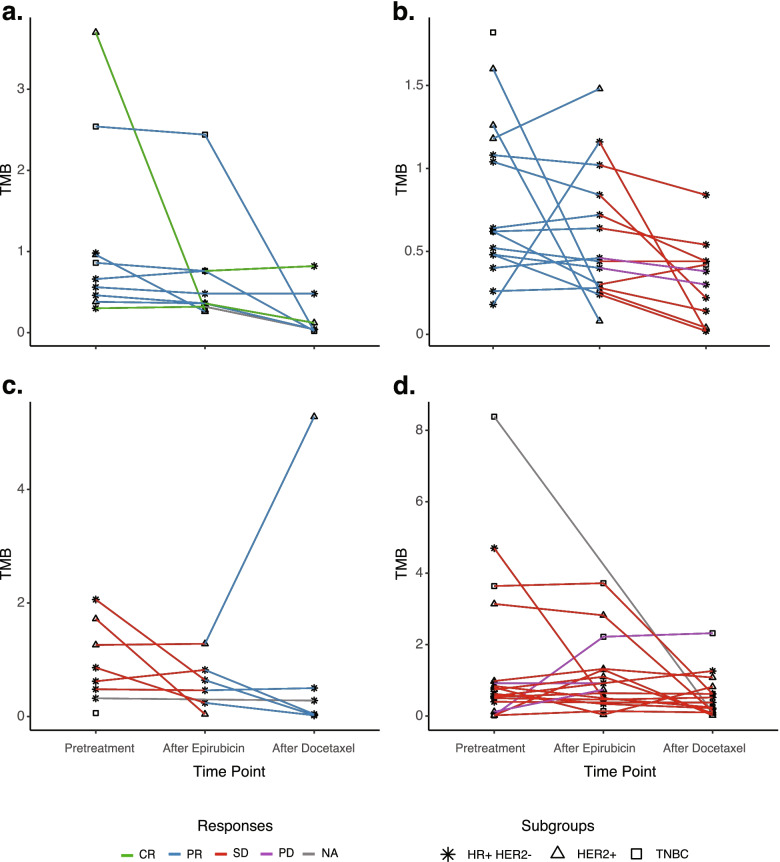
Changes in tumor mutational burden during treatment. Parallel coordinate plots showing changes in tumor mutational burden (TMB) for individual breast cancers undergoing sequential epirubicin and docetaxel*. TMB was assessed by whole exome sequencing (WES) in pretreatment samples, post-epirubicin, and post-docetaxel. *Y*-axes indicate the TMB. Results are split by response groups: **a** objective response (CR, complete response, or PR, partial response) to both drugs. **b** Objective response to epirubicin; no response to docetaxel (SD, stable disease, or PD, progressive disease). **c** No response to epirubicin; objective response to docetaxel. **d** No response to either epirubicin or docetaxel. Green lines: CR, blue lines: PR, red lines: SD, purple lines: PD, gray line: non-evaluable response (NE). Single point, without line: only one biopsy available for analysis. Asterisk: hormone receptor positive, HER2 normal (HR+HER2−) tumors; triangle: HER2+ tumors; squares: triple-negative breast cancers (TNBC). A significant drop in TMB was observed under epirubicin treatment, among objective responders (*p* = 0.043; left sides of panels **a** and **b**). A significant drop in TMB was also observed under docetaxel treatment and non-responders (*p* = 0.006; right sides of panels **b** and **d**). *HER2-positive breast cancers received concomitant docetaxel and trastuzumab

Among the 51 patients selected for WES, 48 tumors were re-biopsied after epirubicin treatment and analyzed by WES. Within this subset, the mean TMB dropped significantly for epirubicin responders (pretreatment: 0.9 mutations per MB, after epirubicin: 0.62 mutations per MB; *p* = 0.043; Fig. 2a, b), whereas no significant change was observed among non-responders (1.12 and 0.94 mutations per MB, respectively; Fig. 2c, d).

Among mutations which disappeared during epirubicin treatment, *TP53* mutations were lost in 7/18, *PIK3CA* in 2/14, *PTEN* in 3/6, *ATM* in 3/3, *CDH1* in 2/6, *ARID1A* in 2/3, *GATA3* in 1/5, and *JAK2* in 1/3 tumors (Additional file 3: Fig. S5).

*TP53* mutations emerged in four, *PIK3CA* in one, and *GATA3* in one tumor during epirubicin treatment (Additional file 3: Fig. S5). Notably, for all emerging mutations (*n* = 323 mutations in 19 tumors), we re-examined the raw data to assess whether any mutations were present in small subclones pretreatment. For 67 out of 323 mutations (18 out of 19 tumors), 1–5 sequencing reads were detected indicating that even though escaping formal mutation calling, these variants were present at very low variant allele frequencies (VAFs). Among mutations emerging during epirubicin treatment in the above-mentioned predefined breast cancer genes (mutations seen in *TP53*, *PIK3CA*, and *GATA3*), only one of the *GATA3* variants was observed in the pretreatment data.

Furthermore, we detected a numerical decrease of C>T substitutions during epirubicin treatment, but this decrease did not reach statistical significance (mean number of mutations before and after epirubicin, 22.4 versus 16.5; *p* > 0.05; Additional file 3: Fig. S8c).

**Fig. 3:**
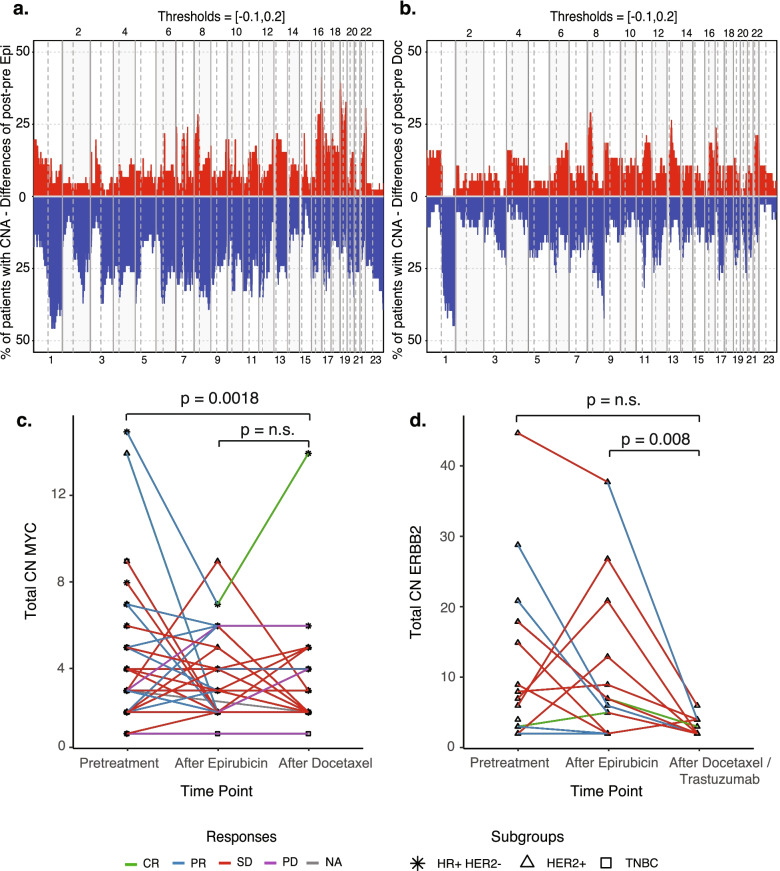
Dynamics of copy number alterations during treatment. **a**, **b** Changes in copy number alterations (CNA) during sequential epirubicin and docetaxel treatment* **a** pre- to post-epirubicin and **b** pre- to post-docetaxel. Bars indicate the differences in fraction of patients with CNA (i.e., the difference: fraction of patients with CNAs in post-treatment samples minus the corresponding number in pretreatment samples). The *Y*-axis indicates the difference in copy number losses (blue) and gains (red). Chromosome numbers are indicated on the *X*-axis. Chromosomes and chromosome arms are separated by vertical lines and shaded background. For specific CNAs called as driver events in biopsies pretreatment, after epirubicin, and/or after docetaxel in individual patients, see Additional file 3: Fig. S10. **c** Parallel coordinate plots showing total copy number changes for *MYC* across the three time points (pretreatment, post-epirubicin, and post-docetaxel*), *n* = 51 tumors analyzed by whole exome sequencing (WES). The *Y*-axis indicates copy numbers, and the *X*-axis indicates the time points. Lines are colored based on the response to each of the two treatments. Asterisk: hormone receptor positive, HER2 normal (HR+HER2−) tumors; triangle: HER2+ tumors; squares: triple-negative breast cancers (TNBC). *MYC* copy numbers were significantly lowered during docetaxel treatment. **d** Parallel coordinate plots showing total copy number changes for *ERRB2* across the three time points (pretreatment, post-epirubicin, and post-docetaxel/trastuzumab), *n* = 15 tumors/patients analyzed by WES. The *Y*-axis indicates copy numbers and the *X*-axis the time points. Lines are colored based on the response to each of the two treatments. Asterisk: HR+HER2− tumors, triangle: HER2+ tumors, squares: TNBC. *ERBB2* copy numbers are significantly lower after docetaxel and trastuzumab treatment as compared to post-epirubicin tumors. *HER2-positive breast cancers received concomitant docetaxel and trastuzumab

Importantly, we observed major changes in CNA in samples collected after epirubicin as compared to before treatment. The most prominent changes were an increase in copy number losses in chromosomes 1q, 2q, 3q (*PIK3CA*, *SOX2*) and 6 and 8q (*MYC*) and an increase in copy number gains in chromosomes 16q and 19 (*STK11*, *AKT2*; Fig. 3a). These copy number changes were similar among epirubicin responders and non-responders.

Finally, no single mutation or set of mutations in key breast cancer pathways detected in post-epirubicin biopsies predicted response to subsequent docetaxel treatment (Additional file 4: Table S7, Additional file 3: Fig. S5). Also, no somatic mutation signatures in post-epirubicin biopsies were associated with response to docetaxel (Additional file 3: Fig. S8b). For HER2-positive tumors, no genomic aberrations characterized tumors responding or not responding to docetaxel + trastuzumab.

### Mutational spectrum at surgery, after epirubicin and docetaxel

After completing docetaxel treatment, tumor specimens for repeated WES were available at surgery from 43 out of the 51 patients subjected to pretreatment WES. The mean TMB was significantly decreased after docetaxel treatment (pre-docetaxel: *n* = 0.84, post-docetaxel: *n* = 0.52; *p* = 0.0011). While no significant TMB reduction was observed among responders (pre-docetaxel: *n* = 0.78, post-docetaxel: *n*=0.68; *p*=0.15; Fig. 2a, c), a significant decrease in TMB was observed among non-responders to docetaxel treatment (pre-docetaxel: *n* = 0.88, post-docetaxel: *n* = 0.48; *p* = 0.006; Fig. 2b, d).

Among mutations disappearing during docetaxel treatment, *TP53* mutations were lost in 6/13, *PIK3CA* in 6/13, *CDH1* in 2/4, and *GATA3* in 3/5 tumors (Additional file 3: Fig. S5).

*TP53* mutations emerged in two, *ATM* in one, and *CDH1* in one tumor during docetaxel treatment (Additional file 3: Fig. S5). In the raw data, 56 out of the 155 variants which emerged during docetaxel treatment had 1–5 sequencing reads in biopsies collected after epirubicin treatment, indicating their presence before commencing docetaxel. Notably, this was not the case for any of the mutations in the key breast cancer genes *TP53*, *ATM*, and *CDH1* emerging during docetaxel treatment (see above), indicating that these variants may have been acquired during docetaxel.

We detected a significant decrease in C>T, T>C, and C>A substitutions (*p*-values = 2 × 10^−3^, 4 × 10^−4^, and 0.015, respectively) during docetaxel treatment (Additional file 3: Fig. S8d).

Also, we observed a general increase in copy number losses in chromosome regions 1q and 8q (*MYC*) and an increase in copy number gains in 8p (*FGFR1*, *WHSC1L1*) after docetaxel as compared to after epirubicin treatment (Fig. 3b). While all of these three types of CNA changes were observed during epirubicin treatment, they were also observed during docetaxel treatment. Assessing the *MYC* gene specifically, we found a significant increase in copy number losses from the pretreatment setting to the end of neoadjuvant treatment (i.e., after epirubicin and docetaxel treatment; *p* = 0.0018; Fig. 3c).

For HER2-positive tumors, a significant decrease in copy numbers of *ERBB2* on chromosome 17 was observed after docetaxel + trastuzumab as compared to post-epirubicin tumors (*p* = 0.008; Fig. 3d). This contrasted with HER2-negative tumors where no corresponding changes were observed after docetaxel monotherapy. Apart from this, there were no other mutational changes which characterized HER2-positive breast cancers, subsequent to docetaxel and trastuzumab treatment.

### Subclonal redistribution during epirubicin treatment

**Fig. 4:**
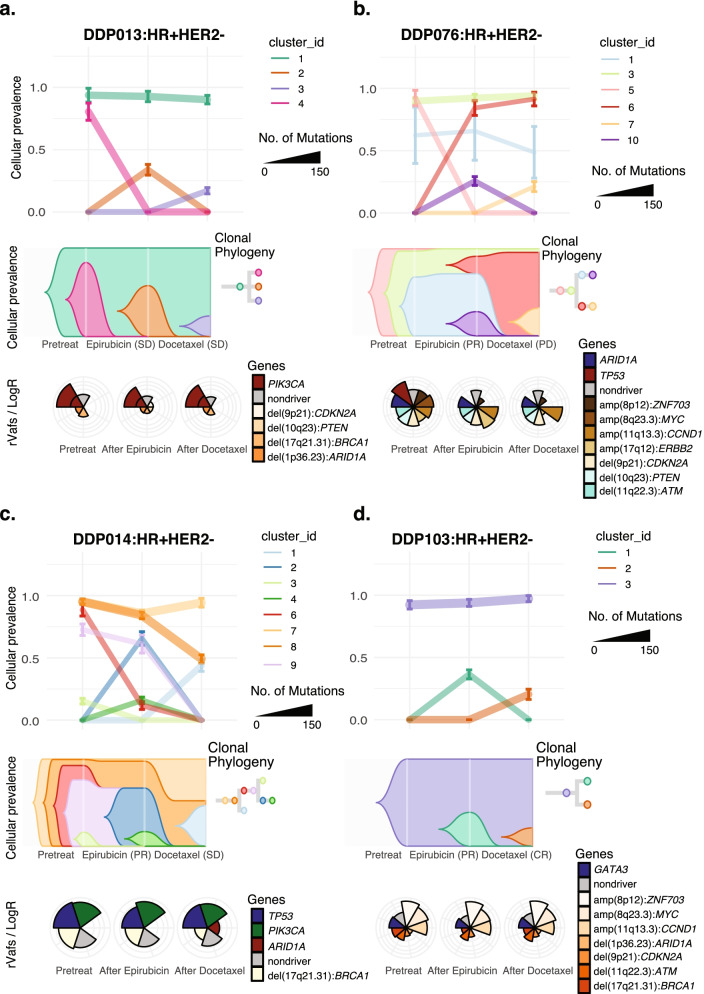
Clonal evolution during treatment. Graphical representation of clonal evolution during sequential epirubicin and docetaxel*, for selected patients: **a** DDP013, **b** DDP076, **c** DDP014, and **d** DDP103. The top panel within each subfigure (**a**–**d**) show allelic prevalence of mutation clusters, after clustering using the PyClone algorithm (see Additional file 1), based on variant allele frequencies (VAFs) for all mutations in the samples extracted for each patient. “Clusters” with one mutation have been merged to nearest cluster based on *z*-score (range of −1, +1) probability. Subsequent clusters with <3 mutations have been removed from the panel, for clarity. The middle panel within each subfigure is a visualization of a likely pattern of tumor evolution using the “timescape algorithm” (see Additional file 1). These models are based on the clusters in the top panels. Diagrams do not distinguish between new subclones appearing, harboring all truncal mutations and those appearing that have lost some truncal mutations. Each color represents an estimated subclone from the mutation clusters. The horizontal axis denotes three different time points during tumor evolution: pretreatment, post-epirubicin, and post-docetaxel. The bottom panel within each subfigure (coxcomb plots) represents somatic aberrations (mutations and copy number alterations; CNAs) for each time point (pretreatment, post-epirubicin, and post-docetaxel). Gray wedges represent merged “passenger mutations” while colored wedges represent driver somatic mutations and driver CNAs. Relative variant allele frequencies (rVAFs) as well as logR are presented by lateral extension of an outlined wedge. *HER2-positive breast cancers received concomitant docetaxel and trastuzumab

Based on WES data with sufficient tumor cellularity (≥ 20%) in both pre- and post-epirubicin biopsies, subclonal evolution was assessed in 19 out of 51 patients (Additional file 3: Figs. S9-10). Overall, the analysis demonstrated that certain subclones were eradicated by epirubicin in responding tumors, while other subclones remained or expanded. While the majority of truncal subclones remained during epirubicin treatment, the largest shifts in subclone sizes were related to subclones that were relatively small in size, as exemplified in tumors DDP013 and DDP076 (Fig. 4). Interestingly, in 18 out of 19 breast cancers, we detected at least one subclone which disappeared during epirubicin treatment. The only exception (DDP103) was a patient where all pre-epirubicin mutations were clonal (no subclones present; Fig. 4). Notably, assessing the raw data beyond conventional mutation calling, we found traces (sequencing reads) of the disappearing subclones in post-epirubicin biopsies from 17 out of 18 investigated cases, indicating that these subclones were not entirely eradicated.

Furthermore, we observed novel mutations after epirubicin treatment in all 19 patients with eligible paired tumor samples, indicating the emergence of new subclones during epirubicin treatment. However, while 35 subclones appeared (*n* = 19 patients), traces of 28 of these were present in pretreatment biopsies when re-examining the raw data. This indicates that the majority of subclones emerging during epirubicin treatment were present at low level pretreatment, rather than being new, acquired subclones.

Importantly, epirubicin substantially changed the composition of subclones in the tumors, even in patients revealing clinical stable disease during treatment. Thus, even in tumors with stable disease, some subclones seemed to be eradicated, while other subclones expanded (Additional file 3: Fig. S9; DDP085 and DDP089).

The number of subclones in pretreatment tumors was not predictive of response to epirubicin. Despite the observation that subclones emerged or disappeared in individual tumors, the numerical differences in subclones in pre- versus post-epirubicin biopsies were small. Typically, the changes in the absolute number of subclones were between zero and two, thus precluding formal statistical assessment.

**Fig. 5:**
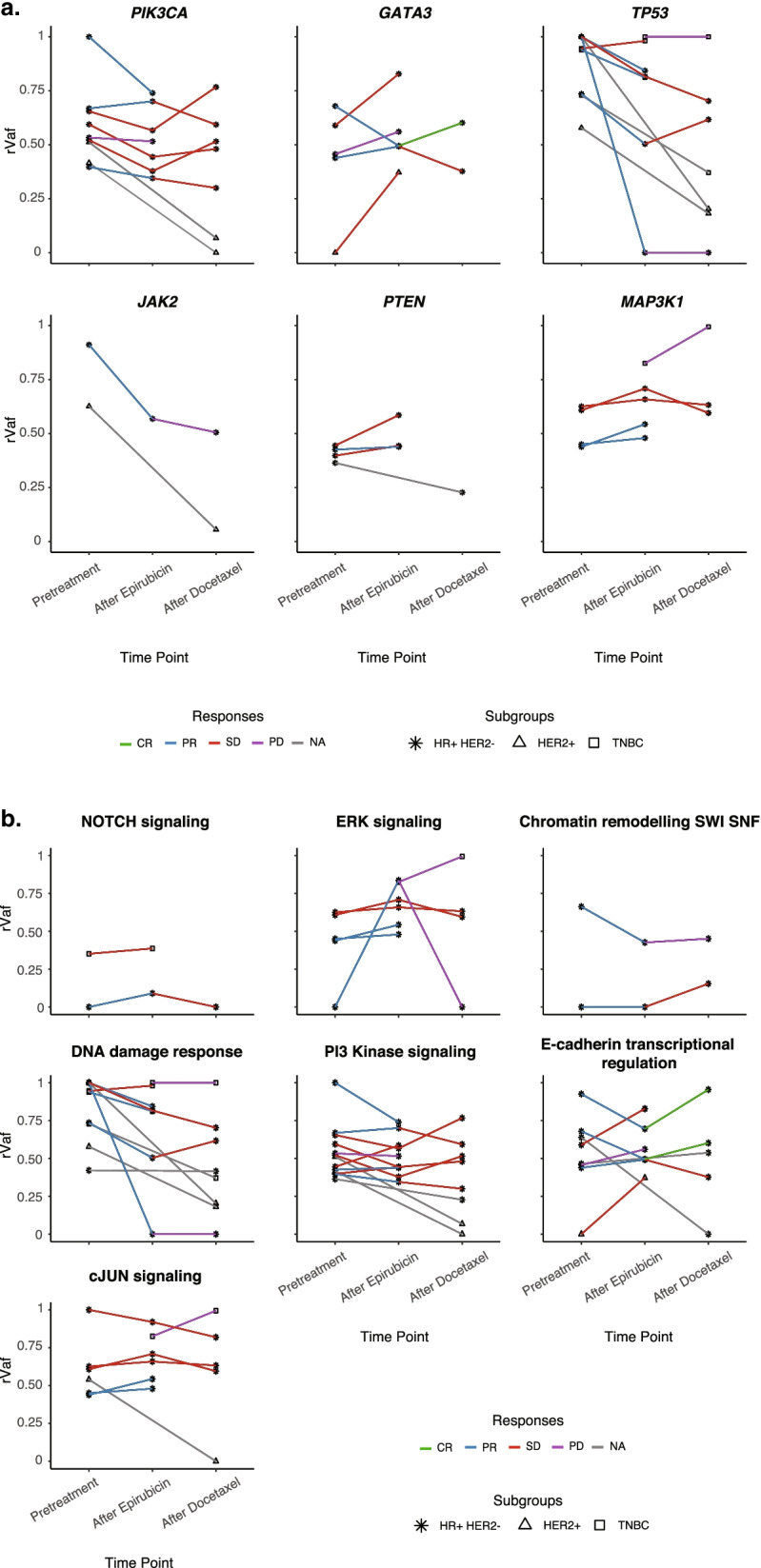
Changes in allele fractions of key drivers during treatment. **a** Parallel coordinate plots showing the relative variant allele frequency (rVAF; corrected for tumor cell fraction) for mutations in key breast cancer genes, across sequential epirubicin and docetaxel* for individual patients harboring mutations in these genes. Lines are colored based on the response to each of the two treatments. Asterisk: hormone receptor positive, HER2 normal (HR+HER2−) tumors; triangle: HER2+ tumors; squares: triple-negative breast cancers (TNBC). **b** Plots for alterations in signaling pathways/biological processes. The *Y*-axis indicates rVAF for mutations affecting genes involved in the illustrated pathways, while the *X*-axis indicates the three time points (pretreatment, post-epirubicin, and post-docetaxel). Lines are colored based on the response of each of the two treatments. Asterisk: HR+HER2− tumors, triangle: HER2+ tumors, squares: TNBC. *HER2-positive breast cancers received concomitant docetaxel and trastuzumab

Furthermore, mutations in established breast cancer genes were examined in subclones that expanded or regressed within each tumor. In line with our previous finding that truncal mutations generally persist through treatment in the metastatic setting [6], mutations in established breast cancer driver genes remained through epirubicin treatment in the current analysis. This was the case for *PIK3CA*, *PTEN*, and *MAP3K1* (Fig. 5a). Notably, for these genes, relative variant allele frequencies (rVAFs) in individual tumor pretreatment were typically 0.5 or higher, indicating an early truncal occurrence. Although the number of observations was low, rVAFs of *GATA3* mutations seemed to increase during epirubicin treatment, indicating growth of *GATA3*-mutated subclones.

Similarly, when mutations were grouped according to signaling pathways/biological processes, the majority of mutations in key breast cancer-related processes persisted through epirubicin treatment (Fig. 5b).

### Subclonal redistribution in response to docetaxel treatment

Subclonal evolution was assessed in 17 out of 51 patients with sufficient tumor cellularity (≥20%) in biopsies before and after docetaxel treatment (Additional file 3: Fig. S9-10). Paired biopsies were available in 11 out of 17 patients immediately before and after docetaxel treatment, whereas six patients had insufficient tumor cellularity after epirubicin treatment (before docetaxel). For these six patients, post-docetaxel samples were compared to pre-epirubicin samples.

Docetaxel mediated profound subclonal redistribution, which was clearly different from changes induced by epirubicin treatment (exemplified by patient DDP014; Fig. 4).

In 16 out of 17 patients, at least one tumor subclone disappeared during docetaxel treatment. This was observed in 10 out of 11 patients with biopsies extracted before and after docetaxel and in all of the six patients where biopsies were taken before epirubicin and after subsequent docetaxel. The only exception was a patient where all pre-docetaxel mutations were clonal (DDP063; Additional file 3: Fig. S9).

Notably, assessing the raw data beyond conventional mutation calling, we found traces (sequencing reads) of the subclones which disappeared in all 16 patients. This indicates that subclones which were no longer present after docetaxel, according to the preset detection limits, were not completely eradicated by the treatment. At the same time, for all patients (*n*=17), new subclones were observed, after docetaxel treatment, indicating (as for epirubicin) that even in tumors with profound regression, small subclones may emerge during chemotherapy. Notably, among the 24 subclones seemingly appearing under docetaxel treatment, we found raw sequencing reads supporting the presence of 11 of these in the pre-docetaxel sequencing data. This indicates that nearly half of the “new” subclones in fact expanded from smaller subclones, rather than being acquired during docetaxel treatment.

Only two patients with an objective response had adequate quality (high tumor cell fraction) in both pre- and post-docetaxel biopsies, thus precluding comparison of responders versus non-responders with respect to numbers of subclones appearing/disappearing under docetaxel treatment. Notably, similar to the observations under epirubicin treatment, we also found docetaxel to have a profound effect on subclonal composition, even in tumors without an objective clinical response to chemotherapy (Fig. 4; DDP014 and DDP076).

Interestingly, among pretreatment subclones which shrunk below detection limits during epirubicin treatment, none of these re-emerged during docetaxel, indicating that they were probably eradicated by epirubicin.

We further characterized the mutations in subclones that expanded or regressed within each tumor under docetaxel treatment. Similar to what was observed during epirubicin, most truncal mutations affecting key breast cancer genes remained through docetaxel treatment (Fig. 5a). Also, the majority of mutations in key breast cancer-related processes persisted through docetaxel treatment (Fig. 5b). Of notice, the number of samples examined to make these comparisons is low, and the data should be interpreted with caution.

Notably, we sequenced tissue samples after docetaxel treatment for four of the patients that were classified as pCR. In three out of these four, we detected somatic mutations. All four had remaining ductal carcinoma in situ (DCIS) after treatment and most likely the mutations detected after treatment were from the DCIS tissue component.

Finally, we assessed the subclonal evolution during epirubicin or docetaxel monotherapy, or as a results of both sequential treatments, split by HR and HER2 status. This was performed in all 27 cases where biopsies from at least two out of three time points were analyzed. Among these cases, 18 were HR positive and HER2 negative and five were HER2 positive (out of which four were HR positive), while four were TNBC. Although the numbers of HER2-positive and TNBC cases were limited and results should be interpreted with caution, we did not observe any specific differences in the subclonal dynamics with respect to breast cancer subtypes (Additional file 3: Fig. S9).

## Discussion

In the present study, we aimed at assessing clonal evolution in treatment-naïve breast cancers during neoadjuvant chemotherapy. Thus, we applied WES to map the genetic alterations in biopsies collected before, at therapy switch, and after treatment with sequential epirubicin and docetaxel monotherapy.

Comparing pre- and post-treatment cancer genomes is challenging due to a decreasing content of cancer cells during therapy. For this reason, we included patients with large primary breast cancers to compare pretreatment to post-epirubicin and post-docetaxel sequencing results and to obtain longitudinal data across the two main breast cancer chemotherapeutics. With respect to resistance mechanisms, the most interesting pre- and post-treatment genomic comparisons may be in those tumors where a substantial amount of invasive cancer remains after treatment. In our clinical trial cohort, 27 out of 51 post-treatment samples had more than 20% tumor cellularity, allowing the comparison of tumor genomic landscapes before and after therapy. Doing so, we identified several evolutionary patterns of breast cancer during each of the two treatments. Similarly to a previously reported evolutionary pattern from primary to metastatic breast cancer [6], we found that pretreatment truncal clones generally persisted through neoadjuvant chemotherapy, seemingly regardless of which driver mutations that were present. This opens the intriguing question of whether, in a majority of breast cancers, mechanisms involved in tumorigenesis in early treatment-naïve disease are also key factors for subsequent treatment resistance [40].

Despite the persistence of truncal mutations in most patients, we observed a profound subclonal redistribution during neoadjuvant chemotherapy: some subclones shrunk and/or disappeared during epirubicin and subsequent docetaxel treatment, while other tumors seemingly acquired mutations as part of new subclones emerging. Strikingly, the majority of these changes were related to subclones of lower VAFs rather than the larger truncal clones, suggesting a more recent origin during tumorigenesis. Moreover, there were cases where a small, pre-existing subclone grew into a major subclone, without gaining new mutations during the treatment period. Importantly, profound redistribution of the subclonal composition was observed also in tumors with no objective treatment response. Thus, even in breast cancers with stable or progressive disease upon treatment, epirubicin and docetaxel seemed to eradicate a substantial number of subclones, whereas other subclones of resistant cells expanded in the same timespan.

While some of the mutations emerging during treatment were in key driver genes, these may have been present in a low fraction of cells, below detection limits, in the pretreatment setting [41]. Re-analyzing our original raw data, we observed that the majority of subclones that seemingly appeared during epirubicin treatment were in fact present at very low levels pretreatment. Furthermore, almost half of the subclones which emerged during docetaxel treatment pre-existed at low levels prior to commencing docetaxel. Of notice, we did not perform ultra-deep sequencing to fully evaluate the fraction of subclones with pre-existing mutations. Thus, our findings may have underestimated the number of mutations that were actually present in minor subclones pretreatment. At the same time, our findings are largely in line with a previous study where a single-cell approach was used to identify pre-existing mutations emerging during chemotherapy in TNBC [42]. In those cases where emerging mutations could not be detected in pretreatment samples, one may speculate whether or not some of these are in fact induced by the cytotoxic compounds, although it seems unlikely that a large number of mutations would be induced by chemotherapy in such a short-lasting neoadjuvant treatment window.

Apart from a smaller investigation of subclonal evolution in 20 patients with TNBC [42], the mechanisms by which chemotherapy influences the breast cancer genome and subclonal distribution has only been assessed to a limited extent. In a study of 47 patients, genetic diversity was examined in biopsies before and after completed neoadjuvant combination chemotherapy, using in situ hybridization for a small number of genomic loci [43]. The authors established that genetic diversity is an intrinsic trait which remains relatively stable during treatment for each individual tumor. We confirm this finding in our samples, but we reveal a much higher complexity and dynamic changes of the smaller subclones during neoadjuvant treatment. A study of 29 patients selected due to lack of response to neoadjuvant chemotherapy (NAC) [44] found that therapy did not induce any chances to allelic profiles in the majority of tumors. Our data indicate the opposite, with large CNA changes both during epirubicin and docetaxel treatment.

Recently, Denkert et al. [45] found mutational signatures linked to HRD and APOBEC activity to be predictive of pCR after neoadjuvant chemotherapy in estrogen receptor (ER)-positive, HER2-negative, but not in ER-negative, breast cancers. Furthermore, a signature associated with BRCA-mediated DNA repair has previously been suggested as predictive of pCR among 29 patients receiving neoadjuvant dose-dense anthracycline and taxane chemotherapy [46]. While no mutational signature was predictive of chemotherapy response in the current analysis of pretreatment biopsies, our results should be interpreted with caution due to a low number of patient samples. Regarding mutations emerging or disappearing during treatment, these were too few for formal assessments of mutational signature changes in our material.

Of notice, we observed no profound changes in the mutational processes from the pre- to post-treatment setting. This is in line with previous findings comparing primary to metastatic breast cancer, where the same mutational processes seemed to persist within each patient over time, with the only exception being a slight increase in the contribution from APOBEC-related signatures [6, 47]. While our present study focused on subclonal genomic evolution, Echeverria et al. found differences at the transcriptomic and proteomic levels when comparing patient-derived TNBC xenografts collected before and after doxorubicin and cyclophosphamide [48]. Furthermore, in patients with TNBC and non-pCR after neoadjuvant chemotherapy, specific expression profiles have been identified in the remaining (resistant) tumor tissue [49]. Also, changes in hormone receptors and HER2 expression as part of disease progression are important alterations potentially modulating treatment response [50]. However, in the present study, there was insufficient tumor material for repeated analysis of these biomarkers *after* treatment. In essence, for future trials, multi-omics approaches are clearly needed to elucidate all molecular mechanisms involved in sensitivity or resistance to neoadjuvant treatment.

While our study focused on chemotherapy, previous studies have assessed the selection of subclones under treatment with more targeted drugs. In ER-positive breast cancers undergoing endocrine treatment, emerging *ESR1* mutations are well established as a likely resistance mechanism [51]. In a comprehensive analysis, Razavi and colleagues found that mutations in *ESR1*, *MAPK*, and ER transcriptional regulators were selected for during endocrine therapy. However, the majority of cases had other, unknown causes of resistance [9], indicating potentially complex mechanisms at play.

Apart from parameters selecting patients for primary endocrine therapy, like high ER expression and intrinsic luminal subtypes, HER2 overexpression predicting benefit from adding trastuzumab/pertuzumab and, more recently, a potential benefit from adding platinum-compounds or immune checkpoint inhibitors in TNBC [52–55], there are currently no validated predictive biomarkers to guide the selection of particular NAC regimens for individual patients. Of notice, PD-L1 expression was not predictive of response to immune checkpoint inhibitors on top of standard NAC regimens [54, 55], whereas the predictive value of homologous recombination deficiency (HRD) for PARP inhibitor efficacy still needs further validation in primary TNBC [56]. While inactivating *TP53* mutations are predictive of resistance to low-dose anthracycline and mitomycin-based NAC, results with today’s anthracycline regimens, at higher doses or combined with cyclophosphamide, are at variance [57–60]. Thus, clinical trials aiming to identify additional predictive biomarkers are warranted. We have previously demonstrated that inactivation of p53 signaling, caused by *TP53* or *CHEK2* mutations or low *ATM* gene expression, is associated with resistance to low or conventional doses of anthracyclines, but not to taxanes [58, 61–63]. In the present analysis, *TP53* mutations were not predictive of response to epirubicin, but there was a trend towards worse disease-specific survival and earlier relapses among patients with hotspot *TP53* mutations, as compared to wildtype *TP53* status. Compared to our previous studies examining the predictive impact of *TP53* mutations, the current trial used a dose-dense epirubicin regimen, which may have circumvented anthracycline resistance, similar to previous findings with cyclophosphamide dose intensification [60].

Notably, from a clinical perspective, neoadjuvant therapy aims at obtaining pCR at surgery, based on the correlation between pCR and improved survival outcome [64]. Yet, pCR is rarely obtained for the majority of HR-positive, HER2-negative breast cancers, and pCR is even less likely for large T3, as compared to smaller T1-2 primary tumors [64–66]. In the current trial, we included patients with large primary tumors and obtained pCR rates of 3% for HR+/HER2−, 33% for HER2+ breast cancers, and 30% for TNBC. While pCR rates are generally higher in recent, international trials with combination chemotherapy regimens for TNBC and HER2+ breast cancers, these trials have a large percentage of T1-2 tumors [64, 66] where pCR rates are expectedly higher. In a previous neoadjuvant trial, with similar tumor characteristics to ours, pCR rates were similar to what we obtained [67]. Still, recent improvements in neoadjuvant regimens, such as the implementation of endocrine therapy in strongly ER-positive, luminal breast cancers, pertuzumab for HER2-positive tumors, and platinum and immune checkpoint inhibitors for TNBC, would be considered more preferable if a similar trial was designed today.

## Conclusions

We found both epirubicin and docetaxel monotherapy to cause profound redistribution of smaller subclones in primary breast cancer, while early truncal mutations and the main subclones generally persisted through treatment.

## Supplementary Information

**Additional file 1.** The Dose Dense Trial (Methods and Results) and Sequencing approaches.

**Additional file 2.** Study Protocol.

**Additional file 3: Figure S1.** CONSORT diagram for the Dose-Dense trial. **Figure S2**. Survival in the Dose-Dense trial after a minimum follow-up of five years or until death. **Figure S3**. Average sequencing coverage for each sample subject to whole exome sequencing (WES). **Figure S4**. Average sequencing coverage for each pretreatment tumor sample subject to amplicon-based sequencing. **Figure S5**. Oncoplot depicting the frequency of mutations in tumor samples undergoing whole exome sequencing (WES). **Figure S6**. Merged overview of copy number alterations (CNAs) in the sample set. **Figure S7**. Scatter plot showing Pearson correlation between copy number alterations (CNAs) and tumor mutation burden. **Figure S8**. Estimated contribution of mutational processes for each of the patients according to the classification by COSMIC. **Figure S9**. Graphical representation of clonal evolution from the pretreatment setting to post-epirubicin and post-docetaxel. **Figure S10**. Circos plots, coxcomb plots and heatmaps illustrating copy number logRs and mutation rVAFs pretreatment, post-epirubicin and post-docetaxel.

**Additional file 4: Table S1**. Baseline characteristics. **Table S2a**. 6 most common serious adverse event (grade 2 or more). **Table S2b**. 8 most common adverse event (any grade). **Table S3**. Response to neoadjuvant epirubicin and docetaxel in stage II-III breast cancers, and outcome at surgery. **Table S4**. Treatment response. **Table S5a**. Pretreatment mutations and prediction to epirubicin treatment. **Table S5b**. Pretreatment mutations and prediction to docetaxel treatment. **Table S6**. Mutations in pretreatment biopsies and prediction to epirubicin treatment. **Table S7**. Mutations in post-epirubicin biopsies and prediction to docetaxel treatment.

**Additional file 5: Table S4**. Dose-Dense trial; tumors selected for DNA sequencing, response to epirubicin (EPI) and docetaxel (TAX), surgical outcome and breast cancer characteristics.

## Data Availability

Haukeland University Hospital and the University of Bergen support the dissemination of research data and cooperation between investigators nationally and internationally. Based on the biobank approval for the DDP trial given by the Norwegian Ministry of Health before the study commenced, genomic data are not to be made openly available. After publication and upon formal request, raw sequencing data, including de-identified individual participant data and a data dictionary defining each field in the data set, may be shared according to institutional guidelines, pending project-specific approvals from the Regional Ethics Committee in Norway. Requests are via a standard pro forma describing the nature of the proposed research and extent of data requirements. Data recipients are required to enter a formal data sharing agreement that describes the conditions for release and requirements for data transfer, storage, archiving, publication, and intellectual property. Requests are reviewed by the DDP study team in terms of scientific merit and ethical considerations, including patient consent. An evaluation as described above will typically take 3 months. Requests may be directed to the corresponding author.

## References

[1] Bertucci F, Ng CKY, Patsouris A, Droin N, Piscuoglio S, Carbuccia N (2019). Genomic characterization of metastatic breast cancers. Nature..

[2] The-Cancer-Genome-Atlas-Network (2012). Comprehensive molecular portraits of human breast tumours. Nature..

[3] Yates LR, Gerstung M, Knappskog S, Desmedt C, Gundem G, Van Loo P (2015). Subclonal diversification of primary breast cancer revealed by multiregion sequencing. Nat Med.

[4] Nik-Zainal S, Davies H, Staaf J, Ramakrishna M, Glodzik D, Zou X (2016). Landscape of somatic mutations in 560 breast cancer whole-genome sequences. Nature..

[5] PCAWG I-T (2020). Pan-cancer analysis of whole genomes. Nature..

[6] Yates LR, Knappskog S, Wedge D, Farmery JHR, Gonzalez S, Martincorena I (2017). Genomic evolution of breast cancer metastasis and relapse. Cancer Cell.

[7] Nik-Zainal S, Van Loo P, Wedge DC, Alexandrov LB, Greenman CD, Lau KW (2012). The life history of 21 breast cancers. Cell..

[8] Angelova M, Mlecnik B, Vasaturo A, Bindea G, Fredriksen T, Lafontaine L (2018). Evolution of metastases in space and time under immune selection. Cell..

[9] Razavi P, Chang MT, Xu G, Bandlamudi C, Ross DS, Vasan N (2018). The genomic landscape of endocrine-resistant advanced breast cancers. Cancer Cell.

[10] O'Leary B, Cutts RJ, Liu Y, Hrebien S, Huang X, Fenwick K (2018). The genetic landscape and clonal evolution of breast cancer resistance to palbociclib plus fulvestrant in the PALOMA-3 trial. Cancer Discov..

[11] Hong SP, Chan TE, Lombardo Y, Corleone G, Rotmensz N, Bravaccini S (2019). Single-cell transcriptomics reveals multi-step adaptations to endocrine therapy. Nat Commun.

[12] Cardoso F, Kyriakides S, Ohno S, Penault-Llorca F, Poortmans P, Rubio IT (2019). Early breast cancer: ESMO Clinical Practice Guidelines for diagnosis, treatment and follow-up. Ann Oncol..

[13] Kaufmann M, von Minckwitz G, Mamounas EP, Cameron D, Carey LA, Cristofanilli M (2012). Recommendations from an international consensus conference on the current status and future of neoadjuvant systemic therapy in primary breast cancer. Ann Surg Oncol.

[14] Tryfonidis K, Senkus E, Cardoso MJ, Cardoso F (2015). Management of locally advanced breast cancer-perspectives and future directions. Nat Rev Clin Oncol.

[15] Early Breast Cancer Trialists' Collaborative G. Increasing the dose intensity of chemotherapy by more frequent administration or sequential scheduling: a patient-level meta-analysis of 37 298 women with early breast cancer in 26 randomised trials. Lancet. 2019;393(10179):1440-1452. 10.1016/S0140-6736(18)33137-4 PMC6451189 30739743

[16] Paridaens R, Biganzoli L, Bruning P, Klijn JG, Gamucci T, Houston S (2000). Paclitaxel versus doxorubicin as first-line single-agent chemotherapy for metastatic breast cancer: a European Organization for Research and Treatment of Cancer Randomized Study with cross-over. J Clin Oncol.

[17] Sledge GW, Neuberg D, Bernardo P, Ingle JN, Martino S, Rowinsky EK (2003). Phase III trial of doxorubicin, paclitaxel, and the combination of doxorubicin and paclitaxel as front-line chemotherapy for metastatic breast cancer: an intergroup trial (E1193). J Clin Oncol.

[18] Lonning PE (2003). Study of suboptimum treatment response: lessons from breast cancer. Lancet Oncol.

[19] Therasse P, Arbuck SG, Eisenhauer EA, Wanders J, Kaplan RS, Rubinstein L (2000). New guidelines to evaluate the response to treatment in solid tumors. European Organization for Research and Treatment of Cancer, National Cancer Institute of the United States, National Cancer Institute of Canada. J Natl Cancer Inst.

[20] Hayward JL, Carbone PP, Heuson JC, Kumaoka S, Segaloff A, Rubens RD (1977). Assessment of response to therapy in advanced breast cancer: a project of the Programme on Clinical Oncology of the International Union Against Cancer, Geneva, Switzerland. Cancer.

[21] Storaas E, Holmaas G, Gravdal K, Borretzen A, Eikesdal HP (2013). Lethal pneumonitis after docetaxel chemotherapy: case report and review of the literature. Acta Oncol.

[22] Birkeland E, Zhang S, Poduval D, Geisler J, Nakken S, Vodak D (2018). Patterns of genomic evolution in advanced melanoma. Nat Commun.

[23] Li H, Durbin R (2009). Fast and accurate short read alignment with Burrows-Wheeler transform. Bioinformatics..

[24] McKenna A, Hanna M, Banks E, Sivachenko A, Cibulskis K, Kernytsky A (2010). The Genome Analysis Toolkit: a MapReduce framework for analyzing next-generation DNA sequencing data. Genome Res.

[25] Cibulskis K, Lawrence MS, Carter SL, Sivachenko A, Jaffe D, Sougnez C (2013). Sensitive detection of somatic point mutations in impure and heterogeneous cancer samples. Nat Biotechnol.

[26] Saunders CT, Wong WS, Swamy S, Becq J, Murray LJ, Cheetham RK (2012). Strelka: accurate somatic small-variant calling from sequenced tumor-normal sample pairs. Bioinformatics..

[27] Wang K, Li M, Hakonarson H (2010). ANNOVAR: functional annotation of genetic variants from high-throughput sequencing data. Nucleic Acids Res.

[28] Van Loo P, Nordgard SH, Lingjaerde OC, Russnes HG, Rye IH, Sun W (2010). Allele-specific copy number analysis of tumors. Proc Natl Acad Sci U S A.

[29] Forbes SA, Beare D, Gunasekaran P, Leung K, Bindal N, Boutselakis H (2015). COSMIC: exploring the world’s knowledge of somatic mutations in human cancer. Nucleic Acids Res.

[30] Rosenthal R, McGranahan N, Herrero J, Taylor BS, Swanton C (2016). DeconstructSigs: delineating mutational processes in single tumors distinguishes DNA repair deficiencies and patterns of carcinoma evolution. Genome Biol.

[31] Roth A, Khattra J, Yap D, Wan A, Laks E, Biele J (2014). PyClone: statistical inference of clonal population structure in cancer. Nat Methods.

[32] Wickham H (2016). ggplot2: elegant graphics for data analysis.

[33] Smith M. timescape: Patient Clonal Timescapes. R package version 2020:1.18.0.

[34] Nilsen G, Liestol K, Van Loo P, Moen Vollan HK, Eide MB, Rueda OM (2012). Copynumber: efficient algorithms for single- and multi-track copy number segmentation. BMC Genomics.

[35] Mermel CH, Schumacher SE, Hill B, Meyerson ML, Beroukhim R, Getz G (2011). GISTIC2.0 facilitates sensitive and confident localization of the targets of focal somatic copy-number alteration in human cancers. Genome Biol.

[36] Ciriello G, Gatza ML, Beck AH, Wilkerson MD, Rhie SK, Pastore A (2015). Comprehensive molecular portraits of invasive lobular breast cancer. Cell..

[37] Kandoth C, McLellan MD, Vandin F, Ye K, Niu B, Lu C (2013). Mutational landscape and significance across 12 major cancer types. Nature..

[38] Forbes SA, Beare D, Boutselakis H, Bamford S, Bindal N, Tate J (2017). COSMIC: somatic cancer genetics at high-resolution. Nucleic Acids Res.

[39] Alexandrov LB, Nik-Zainal S, Wedge DC, Aparicio SA, Behjati S, Biankin AV (2013). Signatures of mutational processes in human cancer. Nature..

[40] Lonning PE (2004). Genes causing inherited cancer as beacons to identify the mechanisms of chemoresistance. Trends Mol Med.

[41] Turner NC, Reis-Filho JS (2012). Genetic heterogeneity and cancer drug resistance. Lancet Oncol.

[42] Kim C, Gao R, Sei E, Brandt R, Hartman J, Hatschek T (2018). Chemoresistance evolution in triple-negative breast cancer delineated by single-cell sequencing. Cell..

[43] Almendro V, Cheng YK, Randles A, Itzkovitz S, Marusyk A, Ametller E (2014). Inference of tumor evolution during chemotherapy by computational modeling and in situ analysis of genetic and phenotypic cellular diversity. Cell Rep.

[44] Varna M, Soliman H, Feugeas JP, Turpin E, Chapelin D, Legres L (2007). Changes in allelic imbalances in locally advanced breast cancers after chemotherapy. Br J Cancer.

[45] Denkert C, Untch M, Benz S, Schneeweiss A, Weber KE, Schmatloch S (2021). Reconstructing tumor history in breast cancer: signatures of mutational processes and response to neoadjuvant chemotherapy (small star, filled). Ann Oncol.

[46] Powles RL, Wali VB, Li X, Barlow WE, Nahleh Z, Thompson AM (2020). Analysis of pre- and posttreatment tissues from the SWOG S0800 trial reveals an effect of neoadjuvant chemotherapy on the breast cancer genome. Clin Cancer Res.

[47] Brady SW, McQuerry JA, Qiao Y, Piccolo SR, Shrestha G, Jenkins DF (2017). Combating subclonal evolution of resistant cancer phenotypes. Nat Commun.

[48] Echeverria GV, Ge Z, Seth S, Zhang X, Jeter-Jones S, Zhou X, et al. Resistance to neoadjuvant chemotherapy in triple-negative breast cancer mediated by a reversible drug-tolerant state. Sci Transl Med. 2019;11(488):eaav0936. 10.1126/scitranslmed.aav0936. 10.1126/scitranslmed.aav0936 PMC6541393 30996079

[49] Balko JM, Giltnane JM, Wang K, Schwarz LJ, Young CD, Cook RS (2014). Molecular profiling of the residual disease of triple-negative breast cancers after neoadjuvant chemotherapy identifies actionable therapeutic targets. Cancer Discovery.

[50] Walter V, Fischer C, Deutsch TM, Ersing C, Nees J, Schutz F (2020). Estrogen, progesterone, and human epidermal growth factor receptor 2 discordance between primary and metastatic breast cancer. Breast Cancer Res Treat.

[51] Robinson DR, Wu YM, Vats P, Su F, Lonigro RJ, Cao X (2013). Activating ESR1 mutations in hormone-resistant metastatic breast cancer. Nat Genet.

[52] Poggio F, Bruzzone M, Ceppi M, Ponde NF, La Valle G, Del Mastro L (2018). Platinum-based neoadjuvant chemotherapy in triple-negative breast cancer: a systematic review and meta-analysis. Ann Oncol.

[53] von Minckwitz G, Schneeweiss A, Loibl S, Salat C, Denkert C, Rezai M (2014). Neoadjuvant carboplatin in patients with triple-negative and HER2-positive early breast cancer (GeparSixto; GBG 66): a randomised phase 2 trial. Lancet Oncol.

[54] Schmid P, Cortes J, Pusztai L, McArthur H, Kummel S, Bergh J (2020). Pembrolizumab for early triple-negative breast cancer. N Engl J Med.

[55] Mittendorf EA, Zhang H, Barrios CH, Saji S, Jung KH, Hegg R (2020). Neoadjuvant atezolizumab in combination with sequential nab-paclitaxel and anthracycline-based chemotherapy versus placebo and chemotherapy in patients with early-stage triple-negative breast cancer (IMpassion031): a randomised, double-blind, phase 3 trial. Lancet..

[56] Eikesdal HP, Yndestad S, Elzawahry A, Llop-Guevara A, Gilje B, Blix ES (2021). Olaparib monotherapy as primary treatment in unselected triple negative breast cancer. Ann Oncol.

[57] Kandioler-Eckersberger D, Ludwig C, Rudas M, Kappel S, Janschek E, Wenzel C (2000). TP53 mutation and p53 overexpression for prediction of response to neoadjuvant treatment in breast cancer patients. Clin Cancer Res.

[58] Chrisanthar R, Knappskog S, Lokkevik E, Anker G, Ostenstad B, Lundgren S (2008). CHEK2 mutations affecting kinase activity together with mutations in TP53 indicate a functional pathway associated with resistance to epirubicin in primary breast cancer. PLoS One.

[59] Bonnefoi H, Piccart M, Bogaerts J, Mauriac L, Fumoleau P, Brain E (2011). TP53 status for prediction of sensitivity to taxane versus non-taxane neoadjuvant chemotherapy in breast cancer (EORTC 10994/BIG 1-00): a randomised phase 3 trial. Lancet Oncol.

[60] Lehmann-Che J, Andre F, Desmedt C, Mazouni C, Giacchetti S, Turpin E (2010). Cyclophosphamide dose intensification may circumvent anthracycline resistance of p53 mutant breast cancers. Oncologist..

[61] Chrisanthar R, Knappskog S, Lokkevik E, Anker G, Ostenstad B, Lundgren S (2011). Predictive and prognostic impact of TP53 mutations and MDM2 promoter genotype in primary breast cancer patients treated with epirubicin or paclitaxel. PLoS One.

[62] Knappskog S, Chrisanthar R, Lokkevik E, Anker G, Ostenstad B, Lundgren S (2012). Low expression levels of ATM may substitute for CHEK2 /TP53 mutations predicting resistance towards anthracycline and mitomycin chemotherapy in breast cancer. Breast Cancer Res.

[63] Aas T, Borresen AL, Geisler S, Smith-Sorensen B, Johnsen H, Varhaug JE (1996). Specific P53 mutations are associated with de novo resistance to doxorubicin in breast cancer patients. Nat Med.

[64] Cortazar P, Zhang L, Untch M, Mehta K, Costantino JP, Wolmark N (2014). Pathological complete response and long-term clinical benefit in breast cancer: the CTNeoBC pooled analysis. Lancet..

[65] Houssami N, Macaskill P, von Minckwitz G, Marinovich ML, Mamounas E (2012). Meta-analysis of the association of breast cancer subtype and pathologic complete response to neoadjuvant chemotherapy. Eur J Cancer.

[66] Early Breast Cancer Trialists' Collaborative G (2018). Long-term outcomes for neoadjuvant versus adjuvant chemotherapy in early breast cancer: meta-analysis of individual patient data from ten randomised trials. Lancet Oncol.

[67] Silwal-Pandit L, Nord S, von der Lippe GH, Moller EK, Fleischer T, Rodland E (2017). The longitudinal transcriptional response to neoadjuvant chemotherapy with and without bevacizumab in breast cancer. Clin Cancer Res.

