# Supplementary material for: Clonal evolution in primary breast cancers under sequential epirubicin and docetaxel monotherapy
Source: Genome Med. 2022 Aug 11;14:86. doi: 10.1186/s13073-022-01090-2 (PMC9367103; doi:10.1186/s13073-022-01090-2)
Supplement: Supplementary file 1 — Additional file 1. The Dose Dense Trial (Methods and Results) and Sequencing approaches. [file 13073_2022_1090_MOESM1_ESM.docx]

**Supplementary information**

1. The Dose-Dense trial
2. Massive parallel sequencing procedures
3. **The Dose-Dense trial**

**Methods**

***Patients and study protocol***

The Dose-Dense Protocol (DDP; Clinicaltrials.gov: NCT00496795) was an investigator-initiated single institution single-arm phase II study including patients ≤65 years, with non-inflammatory, primary breast cancers with a tumor size >4 cm and/or N2-3 regional lymph node metastases. Limited metastatic disease (M1) was permitted, provided the inclusion criteria regarding tumor/nodal status were fulfilled and the local primary tumor was considered the main clinical problem. Patients with known allergy to epirubicin or docetaxel were excluded, as were patients with liver enzymes > 2 times upper normal limit or bilirubin > 3 times upper normal limit. Patients deemed unfit for dose-dense chemotherapy were also excluded. Evaluation of exclusion of patients not to receive trastuzumab due to cardiac insufficiency, was made at the physician’s discretion. The treatment regimen consisted of four courses of i.v. epirubicin 60 mg/m^2^ q2w followed by four courses of docetaxel 100 mg/m^2^ q2w (Fig. 1a) prior to surgery, including pegfilgrastim after each chemotherapy course. Patients who did not tolerate docetaxel treatment were shifted to paclitaxel (n=3), 80 mg/m2 qW for the remaining part of the eight-week taxane period. Patients with HER2 positive disease received weekly trastuzumab (4 mg/kg loading dose, then maintenance 2 mg/kg) during neoadjuvant docetaxel treatment, as well as postoperatively, to a total treatment duration of 52 weeks. After surgery, all patients received locoregional radiotherapy and adjuvant systemic treatment according to national guidelines. Endocrine therapy (premenopausal women: tamoxifen, postmenopausal women: aromatase inhibitor (AI) or sequential AI-tamoxifen) was given for five years in case of hormone receptor positive disease.

The primary objective of the study was to identify factors predicting response to dose-intensive epirubicin and docetaxel as sequential therapy in patients with large primary breast cancers and to assess responsiveness to this dose-intensive regimen in a cohort of 60-100 patients (Study protocol, Additional file 2).

All patients were recruited at a single cancer center (Haukeland University Hospital, Bergen, Norway). All patients provided written informed consent prior to any study-specific procedure, and the protocol was approved by the Regional Ethics Committee. Baseline staging included breast MRI, chest X-ray, liver ultrasound, bone scan, electrocardiogram and echocardiography. All patients underwent an incisional breast cancer biopsy (split into snap-frozen tissue stored on liquid nitrogen and tissue for paraffin-fixation). Additionally, a Tru-cut biopsy was obtained after completing epirubicin treatment - immediately before the first docetaxel course. Finally, an incisional biopsy was collected at surgery, after completing docetaxel treatment (Fig. 1a). Blood samples were drawn during screening to extract normal leucocyte DNA.

Clinical tumor measurements (by caliper) were performed before commencing neoadjuvant treatment and again before each subsequent chemotherapy course. Breast MRI exams were performed before and at the end of the initial epirubicin treatment and then again after finalizing docetaxel, immediately prior to surgery. All MRI exams were evaluated individually by two experienced breast radiologists, before reaching a consensus regarding response in each individual patient. Clinical and MRI responses were classified according to Response Evaluation Criteria in Solid Tumors (RECIST) (1). According to the RECIST criteria, an objective response (OR) was either a complete response (CR) or partial response (PR, ≥30% tumor regression) to therapy. For progressive disease (PD), the RECIST criteria require an increase in largest diameter of targetable lesions of ≥20%. For patients with large tumors like in the Dose-Dense trial, we aimed at earlier detection of PD than with the RECIST criteria. Thus, for the definition of PD, we used the more conservative UICC criteria where PD is defined as an increase of ≥25% in the product of the largest and the perpendicular tumor diameter (2).

If PD was observed during epirubicin, neoadjuvant treatment was immediately switched to docetaxel, and in case of PD on docetaxel, further therapy was left to the discretion of the physician. At surgery, the definition of a pathological complete response (pCR) was ypT0 ypN0, where remaining *in situ* carcinoma (is+) was allowed.

Follow-up before the current report was for a minimum of 5 years for each patient from inclusion in the trial, or until time of death. Last follow-up status was extracted in March 2021. No patient was lost to follow-up.

**Results**

***Patient characteristics and clinical outcome***

A total of 109 patients were included in the Dose-Dense trial between November 2007 and February 2016 (Fig. 1a). Out of 109 patients included in the trial, six patients were inclusion failures; with tumors ≤ 4 cm (CONSORT diagram; Additional file 3: Fig. S1). Yet, all 109 patients were included in the formal statistical analyses for therapy response and genomic analyses on an intention-to-treat basis, as they received treatment per protocol and underwent evaluation of treatment response. One patient had M1 disease at inclusion and was excluded from the survival analyses. Neoadjuvant epirubicin was completed according to protocol in 97% (n=106/109 patients) whereas docetaxel was completed in 88% (n=95/108 patients) of the patients (Additional file 3: Fig. S1), i.e., 95 out of 109 patients (87%) completed neoadjuvant chemotherapy per protocol.

Treatment was discontinued due to chemotherapy-related toxicity in 12 patients (epirubicin 1/109, docetaxel 11/108), and due to progressive disease (PD) in two patients (epirubicin 2/109; Additional file 3: Fig. S1 and Additional file 4: Table S2). No dose reductions were prescribed for any patient while on epirubicin but were prescribed for six patients (6.5%) while on docetaxel. Additionally, three patients were switched from docetaxel 100 mg/m2 q2w to paclitaxel 80 mg/m2 qW due to toxicity, facilitating completion of taxane treatment for two out of these three patients. Of notice, changing from docetaxel q2w to paclitaxel qW was a protocol violation, as it was not pre-defined in the protocol. However, these three patients were included in the intention-to-treat analyses for docetaxel treatment, since they continued to receive a taxane.

Two patients were not referred for surgery; one patient due to progression (subcutaneous metastasis emerged, and the patient was no longer eligible for surgery) and another due to a lethal taxane-induced pneumonitis prior to surgery (3).

The median time from the last chemotherapy course to breast surgery was 28.0 days (mean time: 28.5 days).

Response rates were evaluated by clinical examination (with caliper), and radiologically by breast MRI, using the RECIST criteria (1). PD was defined by the UICC criteria (4), as described above. The clinical objective response rate (ORR) after completing sequential epirubicin and docetaxel was 71.6%, where initial epirubicin treatment yielded an ORR of 41.3%, followed by ORR 29.5% for docetaxel after epirubicin. In comparison, for breast MRI the ORR was 91.7%, with ORR 36.2 % for epirubicin and 76.8% for docetaxel, excluding patients where MRI exams were not performed as part of the evaluation of treatment response. In total 13 patients lacked MRI exams in order to evaluate the total ORR, whereas four patients lacked MRI exams to evaluate ORR after completing epirubicin and 14 patients lacked MRI exams to evaluate ORR after completing docetaxel treatment (Additional file 4: Table S3).

Overall, a pCR rate of 15% (n=16) was observed in the Dose-Dense trial. For the different breast cancer subgroups, pCR was observed in 3% of hormone receptor (HR) positive, HER2 normal, 33% HR+/-, HER2 positive and 30% of triple negative breast cancers (TNBC). Among patients with HER2 positive breast cancers, a pCR rate of 15% was observed for HR+/ HER2 positive and 55% for HR-/HER2 positive tumors.

After a median follow-up time of 111 months (range 61 – 160 months) or until death, 25 patients (24%) had experienced a breast cancer recurrence; 15 patients with HR+/HER2- disease, five HER2+ and five TNBC. The median disease-free survival (DFS) was 95 months (range 1-156). Out of 20 patients (19%) who died during follow-up, n=16 patients died due to breast cancer, n=1 died due to the breast cancer treatment (see below), and n=3 died from other causes, not related to breast cancer or breast cancer treatment (Additional file 3: Fig. S2). The median overall survival (OS) was 103 months (range 5-160).

Adverse events registered in patient records were collected retrospectively. The most common adverse event during epirubicin treatment was infection (7.3%, any grade), whereas for docetaxel, hand-foot syndrome was the most common adverse event (46%, any grade and 28%, grade ≥2) (Additional file 4: Table S2). Hospital admittance due to treatment-related adverse events was required for 32 patients during neoadjuvant treatment. The main reason for hospitalization was infection or fever of unknown cause, constituting 66% of all hospital admittances. One patient died from a probable docetaxel-associated pneumonitis (3).

1. **Massive parallel sequencing procedures**

***Library generation and massive parallel sequencing***

From the Dose-Dense trial, samples from 51 patients were selected (Table 1) for whole exome sequencing (WES). Among them, 27 tumors were HR+/HER2-; 15 were HER2+ and nine were HR-/HER2-, i.e. TNBC (Fig. 1b). Eight out of 15 tumors were HR+/HER2+.

For WES, DNA from tumor biopsies collected pretreatment, after epirubicin (before docetaxel) and at surgery was analyzed together with matched blood DNA. In total, 146 tumor samples and 51 blood samples were analyzed. Library preparation and sequencing were performed as previously described (5). In brief, library preparation was performed using the Agilent SureSelectXT Human All Exon V5 kit (covering up to 50 megabases (MB) of exonic sequence). Libraries were paired-end sequenced using Illumina’s TruSeq sequencing by synthesis (SBS) chemistry V3 on a HiSeq2500, resulting in a mean depth of coverage in the targeted regions ranging from 115x to 229x for tumor samples (median across samples: 159.06), and from 49x to 167x for normal samples (median across patients: 68.61). No systematic differences in sequencing quality control (QC) parameters and/or coverage of WES were observed between subtypes of breast cancer. Coverage comparisons between subtypes, within normal samples and tumors pretreatment, after epirubicin and after docetaxel, all yielded p-values >0.7, by Kruskal-Wallis test (Additional file 3: Fig. S3).

Tumor samples from the remaining 45 patients, with available tissue, and their corresponding blood samples underwent amplicon-based targeted sequencing. First, the concentration of amplifiable DNA was quantified by qPCR on the LightCycler® 480 System, using the *KAPA hgDNA quantification and QC kit* (KAPA Biosystems, Roche) with primers for amplifying 129bp DNA according to manufactures protocol. Sequencing library preparation was performed with a custom designed Accel-Amplicon panel from Swift Biosciences. The panel was design to target the following six genes: *PIK3CA, GATA3, TBX3, TP53, CDH1* and *BRCA1* and generated a total of 273 amplicons. 10-25 ng amplifiable DNA was used as input for the multiplex PCR reaction with primers specific for the custom panel genes, with settings according to the specifications by the manufacturer (“Accel-Amplicon and Accel-Amplicon plus panels protocol”, version 3.0). The panel-specific PCR settings were; step1: 98°C- 30sec, step2: 98°C-10sec, step3: 65°C-6 min, step 2-3: 4 cycles, step4: 98°C-10sec, step5: 64°C-1min, step 4-5: 22 cycles, step6:65°C- 1min, step7: 4°C- Hold. Following bead-based size-selection and clean up, the amplicons were dual-indexed by ligation reaction for 20 minutes at 37°C. A final size-selection and cleanup was performed before elution of the amplicon library samples in TE buffer provided in the kit. Library concentration calculations were based on a qPCR assay with *KAPA Library Quantification Kit* (KAPA Biosystems) for Illumina® platforms according to manufacturer’s protocol. Aiming at 5000x in average depth, 16 library samples were multiplexed on each sequencing run. Libraries were pooled equimolar prior to denaturation and further dilution to a final 10pM pool with 1% PhiX spike-in that was sequenced on an Illumina Miseq with Miseq Reagent kit V2. No systematic differences in sequencing QC and/or coverage of the amplicon-based sequencing were observed between subtypes of breast cancer (coverage comparisons between subtypes; p=0.73 by Kruskal-Wallis test; Additional file 3: Fig. S4).

***Data analyses***

Mutation calling

The sequence reads were aligned to the human genome (Build-UCSC hg19) using the BWA-MEM alignment algorithm (6). Sample-wise sorting and duplicate marking was performed on the initial alignments using Picard tools (<http://broadinstitute.github.io/picard>). Indel realignment and base quality recalibration was performed by GATK tools (7). Somatic small variant identification on the matched tumor-normal sample pairs was performed using MuTect (8) (single nucleotide variant [SNV] detection) and Strelka (9) (SNV and small indel detection), using default parameters, and applying the intersect as the true positive for SNVs (5). Only SNVs fulfilling the following criteria were included in further analyses: variant allelic fractions (VAF) of at least 5% and somatic classification support by both MuTect and Strelka. Functional annotation of SNVs and InDels was performed with ANNOVAR (10), and only amino acid changing mutations were included for further analyses.

Copy number analysis

Copy number profiling was performed using the ASCAT algorithm to obtain genomic distributions of gains, losses, loss of heterozygosity (LOH), and number-neutral events as well as the cancer cellular fraction (11). Copy-number alterations (CNAs) for each patient are shown in Circos plots (Additional file 3: Fig. S10).

Mutational signatures

The weights of each mutational signature contributing to an individual tumor sample, with high cosine similarity to 13 mutational processes for breast cancer that have been reported in the COSMIC database, were determined with the R package DeconstructSigs (12, 13). Quality control of the raw input data was performed with the FastQC program (<http://www.bioinformatics.babraham.ac.uk/projects/fastqc>). GATK tool kit was used for calculating duplication ratios, coverage statistics and quality scores based on the alignment files.

Annotation of driver events

To identify likely driver events, we followed a similar approach as we have previously described for breast cancer data sets (14). We used a two-step approach where we first selected the genes most likely to contribute to breast cancer oncogenesis, and then, for each individual mutation within these genes, assessed any evidence indicating a role as a driver mutation. We used published literature to identify the genes most likely to contribute to breast cancer oncogenesis. Also, for each individual mutation that occurred in a high-confidence breast cancer gene, we assigned a likely oncogenic status. Mutations presumed oncogenic were those that met any of the following criteria:

1. Canonical oncogenic mutations in recurrent hotspots.
2. Recurrent mutations in a known oncogene. I.e. ≥2 confirmed non-synonymous SNVs or in-frame deletions previously confirmed at the same position in COSMIC.
3. Likely damaging events in a known tumor suppressor. I.e. truncating, frameshift, essential splice variant or in a mutation hotspot (≥2 somatic mutations) or synonymous mutation in a known recurrent splice-site hotspot.

Oncogenic mutations included previously unreported variants in a high-confidence breast cancer gene that occur within three amino acids of ≥2 confirmed somatic mutations (point 3 above) or truncating events in medium-confidence tumor suppressors (defined as having a known tumor-suppressor role in cancers other than breast cancer).

Estimation of subclonality

As a measure of the cellular prevalence of each mutation, we calculated rVAF of each mutation as the ratio of observed to expected VAF, given local copy number state, tumor cell content and estimated number of mutated alleles.

rVAF=VAFobs/VAFexp=VAFobs/(𝑛mut×𝜌/2×(1−𝜌)+𝑛tot×𝜌),

where nmut refers to the number of mutated alleles, ntot refers to the total copy number at the mutated locus, and ρ refers to the tumor cell content. Sample mutation clustering across samples collected at different time points for each patient (pretreatment, post-epirubicin and post-docetaxel / after neoadjuvant treatment) was performed by use of PyClone (15) and displayed in parallel coordinate- and fish plots (Additional file 3: Fig. S9).

Graphics

All graphics were generated using R version 3.6.1 (http://www.R-project.org/). The “ggplot2()” function was used to generate coxcomb plots (16) Other packages were dplyr, data.table and tidyverse. Time scape and copy number packages from bioconductor were used for the visualization of clonal evolution and copy number alterations respectively (17, 18). Clusters with one mutation have been merged based on z-score (range -1,+1) - probability and clusters with lower than three mutations have been filtered out.

***Statistical analyses***

Assessment of associations between mutations of individual genes and response to therapy was performed by Fisher´s exact test and by trend test across the response groups, using the prop.trend.test package in R. Comparison of continuous variables between groups were performed by Mann-Whitney rank test and by Wilcoxon rank test for paired samples.

Genomic Identifications of Significant Targets in Cancer (GISTIC) 2.0. was used to identify frequent focal- and arm level- amplifications and deletions.

Survival data were assessed by Cox regression analysis calculating hazard ratios for each parameter. For Kaplan-Meier plots, patient subgroups were compared by the log rank test. Event-free survival (EFS) was defined as the interval from time of inclusion to occurrence of disease progression preoperatively, or recurrence of the disease or death from any cause postoperatively. Disease-free survival (DFS) was defined as the interval between surgery and clinical or radiologically confirmed recurrence of breast cancer, patients that were not amenable to surgery were not included in the DFS analysis. Disease-specific survival (DSS) was defined as the interval between inclusion and death from breast cancer, and overall survival (OS) was the interval between inclusion and death from any cause. All patients, except one diagnosed with M1 disease at screening, were included in the survival analyses. Patients with no events were censored at the time of the last patient chart review (March 2021).

General statistical analyses were performed using R, version 3.6.3 or the SPSS 26/PASW 17.0 software package. All p-values reported are two-tailed, and p<0.05 was considered statistically significant.

***Mutational Signatures***

To elucidate how mutational signatures evolved during epirubicin treatment we first assessed the distribution of the six main types of SNVs (C>A, C>G, C>T, T>A, T>C and T>G). Overall, we found a slight, non-significant, decrease in C>T transitions post-epirubicin while the distribution of the other SNV types remained stable (Additional file 3: Fig. S8c).

Further, to characterize how epirubicin influences mutational profiles, we aimed to identify the weight distribution of mutational signatures after restriction to 20 breast cancer mutational signatures by database COSMIC (as shown in Additional file 3: Fig. S8 [R package, DeconstructSigs]). We identified a relative decrease of signature 1, signature 2 and signature 10. At the same time, the prevalence of other mutational signatures increased, such as of signature 3, which is associated with failure in double-strand break repair by homologous recombination, signature 5 with an unknown aetiology and signature 13 which is linked to activity of AID/APOBEC enzymes (Additional file 3: Fig. S8). However, no overall statistically significant trend was detected for changes in these signatures (trend test p = 0.21) with respect to response to chemotherapy.

After treatment with docetaxel, we found a general decrease in C>A and T>C variants (Additional file 3: Fig. S8d). More specifically, in post-docetaxel tumors a decrease in the weights of signatures 1-3, signature 6 and signature 13 was observed.

**References**

1. Therasse P, Arbuck SG, Eisenhauer EA, Wanders J, Kaplan RS, Rubinstein L, et al. New guidelines to evaluate the response to treatment in solid tumors. European Organization for Research and Treatment of Cancer, National Cancer Institute of the United States, National Cancer Institute of Canada. J Natl Cancer Inst. 2000;92(3):205-16.

2. Hayward JL, Carbone PP, Heuson JC, Kumaoka S, Segaloff A, Rubens RD. Assessment of response to therapy in advanced breast cancer: a project of the Programme on Clinical Oncology of the International Union Against Cancer, Geneva, Switzerland. Cancer. 1977;39(3):1289-94.

3. Storaas E, Holmaas G, Gravdal K, Borretzen A, Eikesdal HP. Lethal pneumonitis after docetaxel chemotherapy: case report and review of the literature. Acta Oncol. 2013;52(5):1034-8.

4. Hayward JL, Carbone PP, Heusen JC, Kumaoka S, Segaloff A, Rubens RD. Assessment of response to therapy in advanced breast cancer. Brit J Cancer. 1977;35(3):292-8.

5. Birkeland E, Zhang S, Poduval D, Geisler J, Nakken S, Vodak D, et al. Patterns of genomic evolution in advanced melanoma. Nat Commun. 2018;9(1):2665.

6. Li H, Durbin R. Fast and accurate short read alignment with Burrows-Wheeler transform. Bioinformatics. 2009;25(14):1754-60.

7. McKenna A, Hanna M, Banks E, Sivachenko A, Cibulskis K, Kernytsky A, et al. The Genome Analysis Toolkit: a MapReduce framework for analyzing next-generation DNA sequencing data. Genome Res. 2010;20(9):1297-303.

8. Cibulskis K, Lawrence MS, Carter SL, Sivachenko A, Jaffe D, Sougnez C, et al. Sensitive detection of somatic point mutations in impure and heterogeneous cancer samples. Nat Biotechnol. 2013;31(3):213-9.

9. Saunders CT, Wong WS, Swamy S, Becq J, Murray LJ, Cheetham RK. Strelka: accurate somatic small-variant calling from sequenced tumor-normal sample pairs. Bioinformatics. 2012;28(14):1811-7.

10. Wang K, Li M, Hakonarson H. ANNOVAR: functional annotation of genetic variants from high-throughput sequencing data. Nucleic Acids Res. 2010;38(16):e164.

11. Van Loo P, Nordgard SH, Lingjaerde OC, Russnes HG, Rye IH, Sun W, et al. Allele-specific copy number analysis of tumors. Proc Natl Acad Sci U S A. 2010;107(39):16910-5.

12. Forbes SA, Beare D, Gunasekaran P, Leung K, Bindal N, Boutselakis H, et al. COSMIC: exploring the world's knowledge of somatic mutations in human cancer. Nucleic Acids Res. 2015;43(Database issue):D805-11.

13. Rosenthal R, McGranahan N, Herrero J, Taylor BS, Swanton C. DeconstructSigs: delineating mutational processes in single tumors distinguishes DNA repair deficiencies and patterns of carcinoma evolution. Genome Biol. 2016;17:31.

14. Yates LR, Gerstung M, Knappskog S, Desmedt C, Gundem G, Van Loo P, et al. Subclonal diversification of primary breast cancer revealed by multiregion sequencing. Nat Med. 2015;21(7):751-9.

15. Roth A, Khattra J, Yap D, Wan A, Laks E, Biele J, et al. PyClone: statistical inference of clonal population structure in cancer. Nat Methods. 2014;11(4):396-8.

16. Wickham H. ggplot2: Elegant Graphics for Data Analysis: Springer-Verlag New York; 2016.

17. Smith M. timescape: Patient Clonal Timescapes. 2020.

18. Nilsen G, Liestol K, Van Loo P, Moen Vollan HK, Eide MB, Rueda OM, et al. Copynumber: Efficient algorithms for single- and multi-track copy number segmentation. BMC Genomics. 2012;13:591.
