# Supplementary material for: Clonal evolution in primary breast cancers under sequential epirubicin and docetaxel monotherapy
Source: Genome Med. 2022 Aug 11;14:86. doi: 10.1186/s13073-022-01090-2 (PMC9367103; doi:10.1186/s13073-022-01090-2)
Supplement: Supplementary file 2 — Additional file 2. Study Protocol. [file 13073_2022_1090_MOESM2_ESM.pdf]

Molecular markers predicting response to dose dense chemotherapy with epirubicin and docetaxel in sequence for locally advanced breast cancer

Protocol summary.

Principal Investigator
Per E Lønning, Professor, Section of Oncology, Department of Medicine

Collaborators.

Dept of Surgery –	Responsible: Turid Aas, Consultant Surgeon
Dept of Molecular Biology –	Responsible: Professor Johan Lillehaug
Dept of Anatomy and Cellular Biology –	Responsible: Professor Rolf Bjerkvig

Participants.

Dept of Oncology	Gun Anker,	Consultant Oncologist
	Stephanie Geisler,	Consultant Oncologist
	Jurgen Geisler,	Consultant Oncologist

Type of Study
Phase II, Translational research

Scientific aims: Addressing factors predicting response to dose intensive epirubicin followed by docetaxel sequential therapy

Treatment regimen: epirubicin 60 mg/m^2 on a 2 weekly basis x 4 followed by docetaxel 100 mg/m^2 2-weekly x 4.

Patients: Breast cancer patients below 65 years of age suffering from large (>4 cm largest diameter, non-inflammatory and / or N2-N3) primary breast cancer.

.

Clinical aim: Assessing responsiveness to this dose intensive regimen.

Number of patients to be enrolled: 60 – 100

Aim.
The scientific aim of this study is to explore mechanisms of resistance to chemotherapy in breast cancer. To do so, we explore molecular parameters predicting response to chemotherapy administered prior to local therapy in large, primary breast cancers.

Background
Neoadjuvant (primary medical) therapy has got wide acceptance as primary therapy in breast cancer. In addition, this treatment provides an optimal setting studying the mechanisms of drug resistance in human cancers.

Clinical Therapy
Our center was the first in Norway applying primary chemotherapy for locally advanced primary breast cancers back in 1991-92 (1). For the years to follow, primary medical therapy has become general clinical practice for large primary breast cancers in all Western countries. While there are no randomized data revealing improved long-term survival for primary medical treatment in comparison to ordinary adjuvant treatment (2), justification is based on improved local control. Thus, many inoperable tumors become fit for surgery following primary medical treatment. In addition, the number of local relapses, previously representing a major clinical problem in patients with stage III tumors, has been significantly reduced. Finally, the possibility of assessing tumor response to treatment allows the clinician to terminate non-effective therapy, implementing other drugs in exchange.

While use of primary medical treatment is now considered general practice for locally advanced tumors in Western hospitals, many clinics also use it for primary operable tumors not suitable for local surgical therapy, aiming at reducing their size allowing breast conservation treatment. There is no standard regimen, but most treatments will include an anthracycline and/or taxane, similar to what is used for adjuvant therapy.

Up to now, our practice has been to treat all tumors above 5 cm in diameter and/or T4 (cutaneous or chess wall infiltration) with primary medical treatment before surgery. Many centers around the world now include patients with primary operable tumors suitable for mastectomy but not breast conservation, aiming at local down-staging to allow more limited surgical procedures. Notably, while such therapy in general implements chemotherapy, in many centers it has also become practice to treat postmenopausal women with receptor positive tumors with one of the novel aromatase inhibitors (3). We currently apply such therapy for older patients harboring receptor positive tumors.

Considering chemotherapy for primary treatment, contemporary trend has been to treat these tumors more aggressively. High-dose therapy involving stem cell support is not advocated (4), as this has not been shown to improve long-term survival in early breast cancer. However, the attitude in general has been toward a more aggressive approach within the frame of "conventional" therapy. There are two main reasons for it: first, there are several studies now revealing pathological complete response to primary therapy to predict improved relapse-free survival (5, 6). While such a finding is consistent with the hypothesis that more aggressive therapy improves outcome, there are certain pitfalls, in as much as it could also mean that tumors harboring an intrinsic responsiveness to therapy for biological reasons may have an improved prognosis. Secondly, studies in the adjuvant setting have revealed implementation of taxanes in concert with anthracyclines to improve relapse-free- and overall survival (7, 8).

An important issue relates to drug dosing, in particular with respect to the anthracycline compounds. Following the seminal study by Muss et al (9) reporting optimal benefits to be achieved from a doxorubicin "normal dose regimen" except for patients with tumors overekspressing HER-2, the concept of a "threshold level" with respect to anthracycline dosing in HER-2 negative tumors has been confirmed by studies in the adjuvant as well as the primary chemotherapy setting (7, 10, 11).

Based on theoretical modeling, an alternative approach, "dose-dense" therapy, has been advocated (12). Recently, that concept was brought to the test in two adjuvant trials. Thus, Citron et al applying doxorubicin, paclitaxel and cyclofosfamide revealed an improved outcome for dose-dense (2-weekly) administration compared to regular 3-weekly scheduling (13). In contrast, the German GEPARDUO study reported doxorubicin plus cyclophosphamide and docetaxel, given in sequence on a 3-weekly basis (8 cycles), to be superior to doxorubicin and docetaxel given in concert on a 2-weekly basis for 4 cycles (14). However, the doses administered (doxorubicin 50 versus 60 mg/m2; docetaxel 75 versus 100 mg/m2) was unequal, meaning total drug dose exposure differed between the two treatment arms. While more data are warranted, a reasonable interpretation of available data suggest sequential administration of different compounds in a dose-density approach to be a suitable regimen provided adequate total doses are given.

Implementing our first study on neoadjuvant chemotherapy , we used a "low-dose" regimen (weekly doxorubicin) based on current practice in advanced disease (15). For the next study, we implemented a regimen of conventional doses of epirubicin or paclitaxel (randomized), subsequently to be followed by the alternative drug in non-responders. While the results of that study are currently analyzed, we recognized the overall response-rate here was lower compared to some of the more intensive regimens currently applied. Thus, the clinical justification of the study outlined here is to implement a regimen resembling the recent dose dense regimen that revealed superiority in adjuvant disease (13) for neoadjuvant treatment of women with large primary breast cancers below 65 years of age. Based on clinical experience, we decided to use docetaxel instead of paclitaxel, although there are no randomized data comparing the two regimens.

Rationale for regimen

Much evidence now suggests treatment involving an anthracycline and a taxane either given in concert or sequentially to be optimal therapy in breast cancer. A potential exception is the recent unpublished study from the BCIRG, revealing a platinum compound with a taxane to provide similar efficacy to a taxane – anthracycline combination in HER2 positive tumors without amplification of the topo-II gene in concert (16, 17). Notably, in this group of patients the anthracycline – taxane approach was not inferior to the experimental arm, suggesting the two options may be of equal efficacy. Considering the fact that the number of tumors harboring HER-2 amplifications being Topo-II normal may account for probably 15% of the total population, we select not to develop a separate drug regimen for this sub-group. .

Considering anthracyclines, most regimens today combine either epirubicin or doxorubicin in concert with 5-fluorouracil and cyclophosphamide. However, based on the evidence in the literature, it is not clear what the contribution of 5-FU or cyklophosphamide is to the effectiveness of such regimens (18), in particular not when a taxane is administered in sequence or concert (19).. Thus, the NSABP-group has abandoned use of 5-FU from their adjuvant regimen (20). Considering cyclophosphamide, this compound seems to add to the carcinogenetic effect of anthracyclines enhancing the risk of secondary leukemia (21), while

the contribution to the antitumor efficacy of the regimen remains uncertain. The taxanes are known to have significant antitumor effects in breast cancer when administered as monotherapy (22, 23). Considering docetaxel, the dose generally advocated for monotherapy is 100 mg/m^2, while a dose of 75mg/m^2 is recommended for combined use. (19). Thus, the potential exists that the dose for combined use may be sub-optimal in some patients.

In the recent adjuvant study by Citron et al. (13) they evaluated a dose dense treatment with doxorubicin, paclitaxel and cyklophosphamide in sequence versus doxorubicin plus cyclophosphamide followed by paclitaxel. Each of the 2 regimens was administered at 2- or 3-weekly intervals (2 X 2 factorial design). Total dose of each drug administered was similar in each arm. Here, they recorded similar relapse-free and overall survival between the sequential and combined regimen, but with a clear advantage for the two dose-dense regimens. Thus, the results from this study suggest 1) similar efficacy for sequential and combined treatment provided each drug is administered at similar doses, and 2) an advantage for dose-dense treatment.

Justification for a sequential approach is further supported by studies in metastatic breast cancer (24-27).

Scientific aims

A major issue in breast cancer research (as well as research on other types of cancer) is to explore potential mechanism of drug resistance. Drug resistance is the major reason for therapy failure and subsequent death in cancer patients. In addition to characterizing molecular changes, we aim to identify and cultivate stem cells on an epithelial as well as mesenchymal background in culture to further characterize tumor biology with respect to treatment outcome.

While many mechanisms of resistance have been identified in experimental systems, we have little knowledge what may be the cause of drug resistance in human cancers. The factors so far identified that have been found associated with resistance to chemotherapy in breast cancer are amplification of HER-2 / Topo-II and mutations affecting TP53. Apart from predicting sensitivity to treatment with trastuzumab, overekspression or amplification of the HER-2 gene was found associated with a dose-response to anthracyline therapy in breast cancer not recorded in HER-2 negative tumors (9, 11, 28). However, there is no evidence it may explain the bulk of drug resistance recorded. The reason for this differential sensitivity could be co-amplification of HER-2 with Topo-II (29, 30), a target for anthracycline action cytotoxicity. Notably, patients with tumors harboring Topo-II amplifications do not seem to have an inferior response to anthracyclines; indeed, there is evidence in the literature they may actually be more sensitive to such therapy provided administration at adequate doses (31-33). Considering TP53, we (34) as well as others (35) have found a correlation between TP53 mutations and lack of response to anthracycline therapy in breast cancer. However, TP53 status alone could not fully explain sensitivity, suggesting other genes may interact as well (36).

Recently, several groups have explored responsiveness to chemotherapy using mRNA microarrays. While these studies consistently identified different gene profiles correlating to responsiveness to different regimens (37-41), the predictive value was too low for clinical application. Moreover, the results did not add to our understanding of the biological mechanisms causing drug resistance. In contrast, studies exploring different forms of cancer have started to reveal specific gene alterations, in particular affecting pathways of DNA

damage repair or apoptosis in relation to drug resistance (36). Thus, our primary aim is to explore potential gene disturbances based on functional hypotheses (36, 42).

In an ongoing pilot study (REK 05/3013) vi aim at sampling and cultivating epithelial stem cells from the breast together with mesenchymal stem cells from the bone marrow. While we so far have been unsuccessful identifying an epithelial breast stem cell, we have been able to identify and cultivate mesenchymal stem cells from the bone marrow. Based on recent findings in the literature (43-49), we are hypothesizing these stem cells may play a vital role fueling growth of epithelial cells with a gene damage, suggesting they may be of critical importance to carcinogenesis as well as tumor progression and metastatic seeding. To take this approach forward, we aim at harvesting such stem cells both from the breast but from bone marrow in addition as part of this project.

There are several arguments for choosing a primary medical treatment setting for such studies (50). Allowing direct assessment of tumor response to therapy, such studies provide valuable information from cohorts of less than 100 patients. In contrast, adjuvant studies, having relapse-free or overall survival as endpoints, may need enrollment of more than 1000 patients. Considering metastatic disease, results from ongoing studies by our team suggest a biological heterogeneity between different metastatic deposits from the same tumor making such a model inconvenient (S. Knappskog et al; Unpubl results).

A major issue identifying mechanisms of drug resistance is to explore different compounds on a monodrug basis. Clinically, the major reason for combining or administering sequentially an anthracycline and a taxane is the efficacy of each compound combined with a significant lack of cross-resistance. Administered as a combined schedule, this limits the possibility of identifying the mechanisms of resistance / sensitivity to each compound individually. Provided that a sequential regimen provides the same efficacy as a combined schedule, such an approach is justified ethically. Thus, for a patient given 2 and 3 drugs in concert, the potential may be they respond to one drug only, while having the toxicity of the other compounds (50). In case of non-responsiveness to a single compound, this may be exchanged for an alternative treatment option. This contrasts the possibilities provided in the adjuvant setting. In adjuvant therapy, there is no way by which we may assess the response to individual drugs; thus the different compounds have to administer in concert.

Based on what is said above, we consider sequential dose-dense treatment using epirubicin and docetaxel sequentially to be a feasible treatment option.

Treatment regimen
Each patient will receive 4 cycles of epirubicin 60 mg/m^2 given at 2 weekly intervals together with G-CSF. Thereafter, docetaxel 100 mg/ m^2 will be given at 2 weekly intervals for 4 cycles. Patients revealing positivity for HER2 status will have trastuzumab (Herceptin) implemented together with docetaxel but not during anthracycline treatment. The reason for this is two-fold. First, anthracyclines are shown to work to a similar extent in HER-2 positive tumors compared to HER-2 negative ones, provided the doses administered are in the range of 100mg/m^2 on a 3-weekly basis (11).. Considering the regimen provided here, 60 mg/m^2 on a 2-weekly basis should approach the 100 mg/m^2 3-weekly regimen. Second, administration of an anthracycline and trastuzumab in concert may enhance cardiotoxicity.

Notably, while a Finnish study has shown trastuzumab administered for 9 weeks to provide significant benefits in early breast cancer (51), this study enrolled a limited number of patients, and the results need further confirmation before being implemented in the routine

setting. Currently, general recommendation is to extend trastuzumab treatment on a 3-weekly basis for 51 weeks (52, 53), which is what will be done in this study.

HER-2 status will be evaluated based on common routine criteria in our hospital (immunostaining with FISH /CISH when indicated).

Inclusion Criteria.

- Primary breast cancer >4cm in diameter and / or lymph node status N2-3.
- Age 65 years or younger
- "Limited" distant metastases allowed, but patients with massive distant metastases should be excluded
- Willing to participate in the study

Exclusion criteria.

- Known allergy toward any of the cytotoxic compounds to be administered (epirubicin and doxorubicin)
- Liver enzymes > 2 times upper normal limit or bilirubin > 3 times upper normal limit
- Other medical conditions making them unfit for dose-dense therapy
- Cardiac insufficiency; for patients not to receive trastuzumab, decision whether to exclude such patients will be at the physicians discretion. Considering patients with HER-2 positive tumors who should have trastuzumab, exclusion criteria will be according to the NBCG (Norwegian Breast Cancer Group) general guidelines (www.NBCG.net).

Staging at baseline.

After given informed consent, patients will be staged as follows:

- MRI of both breasts
- Chest X-ray
- Liver ultrasound (in case metastases are suspected or verified; to be followed by CT and / or MRI confirmation)
- Skeletal scintigram. Any positive findings to be confirmed by subsequent X-ray / CT and / or MRI
- ECG
- LVEF (Left Ventricular Ejection Fraction)

Response evaluation.

Response will be evaluated based on clinical examination and MRI, each assessment to be done separately.

Clinical examination will be performed prior to commencing therapy (before surgical biopsy) and subsequently at 4, 6 and 8 weeks on therapy. Response will be classified according to the common "RECIST" criteria (54). An important exception is to be made. As argued in a previous protocol, we consider the RECIST definition of "progressive disease" as a 20% increase in the sum of the largest tumor diameters to be too liberal with respect to large primary tumors. By using such a criterion, it should mean that a tumor with a largest diameter of say 6 cm is allowed to expand to a size of 7.2 cm before considered "non-responsive". Based on experience in our clinic, we believe the definition of progressive disease as an

increase of > 25% in the product of the largest tumor diameter and its perpendicular (the previous common UICC criteria) to be a more suitable definition, protecting patients from undergoing deterioration of their clinical condition. This is in accordance to our previous experience.

In case of "progressive disease" at any stage during epirubicin treatment, the patient will terminate epirubicin immediately and go ahead with docetaxel treatment. In case of progressive disease on docetaxel treatment, further therapy is left to the physician's discretion.

Logistically, clinical response evaluation will be based on a log scheme recording tumor diameters with documentation of which physician performed the measurement. For correct assessment, measurements performed by the same doctor on different occasions are to be compared.

Considering MRI assessment, this should be performed prior to commencing therapy, in the interval following the 4th cycle of epirubicin (prior to commencing docetaxel) and after the 4th cycle of docetaxel, prior to surgery.

Tissue sampling.

1. Each patient will undergo a surgical biopsy prior to commencing treatment. This biopsy should contain between 150 – 500 mg of tissue, to be split into samples snap-frozen (liquid nitrogen) in the theatre, with a single piece for paraffin-fixation. In addition, a small sample will be brought to the laboratory for stem cell culturing.
2. A Tru-cut biopsy should be obtained 24 hours after administration of the first epirubicin cycle to assess "immediate" alterations in response to cytotoxic damage. Of particular interest is to explore changes in protein phosphorylation status (like for p53)
3. Immediately prior to commencing treatment with docetaxel, a third sample (this time a Tru-Cut biopsy) is obtained for snap-freezing.
4. A Tru-cut biopsy should be obtained 24 hours after administration of the first docetaxel cycle to assess "immediate" alterations in response to cytotoxic damage.
5. Finally, tumor tissue is collected and snap-frozen at surgery following docetaxel therapy.

Bone marrow aspiration.

A single unilateral bone marrow aspiration is performed prior to commencing chemotherapy treatment.

Study Endpoint.

- Primary endopoint is to correlate molecular parameters to objective response to each of the 2 regimens applied.
- Secondary endpoint are
:
    - to correlate molecular parameters to relapse-free and overall survival
    - to identify and explore characteristics of epithelial and mesenchymal stem cells isolated in tumor tissue and bone marrow

Treatment regimen.

Epirubicin is administered as 60 mg/m^2, while docetaxel is given as 100 mg/m^2. Both regimens are administered on a 2-weekly basis. Each drug is administered as an i.v. infusion with anti-emetics (and for docetaxel glucocorticoids) in accordance to general treatment administration in our Department.

Due to bone marrow suppression, each patient will be treated with pegfilgrastim (Neulasta) 6 mg injection 24 hours after each chemotherapy cycle.

After each cycle, a blood sample is obtained on day 7-10 to assess for bone marrow toxicity.

Surgery.
While many centers practice breast conservative surgery for tumors successfully downstaged by primary medical treatment, in general we have applied a conservative approach, advocating mastectomy. However, downstaging for limited surgery is not a primary or secondary endpoint of this study. In general, we will advocate mastectomy also for patients with a clinical complete response. However, this practice may change based on contemporary results from other centers, and the protocol allow limited surgery at the physicians discretion in individual patients.

Laboratory Investigations.
The area of molecular biology is rapidly developing with respect to biological knowledge as well as technical analytical methods. Thus, it is not possible to predict upfront which genes may be of particular interest in 5 years from know; neither is it possible to foresee completely which laboratory methods will be available. Our aim is to explore potential genetic alterations explaining the mechanisms of drug resistance. While it is not possible to predict in detail, the aim of the study and all analysis to be conducted should aim at this major goal.

Previously, we identified mutations in the TP53 but also the CHEK2 gene to be associated with lack of responsiveness to anthracycline as well as mitomycin therapy in primary breast cancer (34, 55, 56). Current, we are analyzing other genes for mutations but also promoter hypermethylations aiming at identifying not individual genetic events but rather disturbances in "functional pathways" controlling drug sensitivity (36). Considering we may start the laboratory part of our program in 4-5 years from now, decisions which genes / molecular factors are of most relevance need to be based on contemporary results achived from our own research as well as reports from the literature. As such, we believe studies like this need an "open frame" with respect to individual molecular parameters and also which laboratory techniques are of most relevance.

Considering laboratory experience, our team has the knowledge and experience with common methods in molecular biology including gene sequencing, promoter methylation status, analysis for splice variants and so on (Head of activity; Professor Johan Lillehaug).. In addition, we have collaborations with other teams working with micro-array techniques. With respect to proteomics, we aim at establishing collaborations with other Norwegian research teams with that type of knowledge here in Bergen or at other Norwegian institutions.

While the stem cell research in breast cancer is at an early stage, our team has successfully identified mesenchymal bone marrow stem cells and is well on way characterizing their biology on a general basis. Thus, we have in hand most of the techniques needed for this research activity (Head; Professor Rolf Bjerkvig).

Number of patients to be enrolled

The study is an exploratory translational study. Thus, we do not know the number of patients expected to achieve a clinical or pathological complete response. Currently, on average 20 patients are referred to our Department for a diagnosis of locally advanced breast cancer on an annual basis; including patients with tumors measuring between 4 and 5 cm in diameter, we estimate the total number may be somewhere between 30 and 40 patients per year. From this cohort, we estimate an average number of 20 patients to be enrolled in the study on an annual basis. Our aim is to recruit 100 patients with a minimum of 60.

Publication.

Our aim is to publish the results from this study in peer-reviewed international journals with contributors from the clinic and laboratory as authors.

1. Aas T, Børresen A-L, Geisler S, Smith-Sørensen B, Johnsen H, Varhaug JE, et al. Specific P53 mutations are associated with *de novo* resistance to doxorubicin in breast cancer patients. Nature Med 1996;2:811-814.
2. Fisher B, Bryant J, Wolmark N, Mamounas E, Brown A, Fisher ER, et al. Effect of preoperative chemotherapy on the outcome of women with operable breast cancer. J Clin Oncol 1998;16(8):2672-2685.
3. Eiermann W, Paepke S, Appfelstaedt J, LlombartCussac A, Eremin J, Vinholes J, et al. Preoperative treatment of postmenopausal breast cancer patients with letrozole: A randomized double-blind multicenter study. Ann Oncol 2001;12(11):1527-1532.
4. Bergh J, Wiklund T, Erikstein B, Lidbrink E, Lindman H, Malmstrøm P, et al. Tailored fluorouracil, epirubicin, and cyclophosphamide compared with marrow-supported high-dose chemotherapy as adjuvant treatment for high-risk breast cancer: a randomised trial. Lancet 2000;356:1384-1391.
5. Kaufmann M, von Minckwitz G, Smith R, Valero V, Gianni L, Eiermann W, et al. International expert panel on the use of primary (Preoperative) systemic treatment of operable breast cancer: Review and recommendations. J. Clin. Oncol. 2003;21(13):2600-2608.
6. Smith IC, Heys SD, Hutcheon AW, Miller ID, Payne S, Gilbert FJ, et al. Neoadjuvant chemotherapy in breast cancer: significantly enhanced response with docetaxel. J Clin Oncol 2002;20:1456-1466.
7. Henderson IC, Berry DA, Demetri GD, Cirrincione CT, Goldstein LJ, Martino S, et al. Improved outcomes from adding sequential paclitaxel but not from escalating doxorubicin dose in an adjuvant chemotherapy regimen for patients with node-positive primary breast cancer. J. Clin. Oncol. 2003;21(6):976-983.
8. Martin M, Pienkowski T, Mackey J, Pawlicki M, Guastalla JP, Weaver C, et al. Adjuvant docetaxel for node-positive breast cancer. N. Engl. J. Med. 2005;352(22):2302-2313.
9. Muss HB, Thor AD, Berry DA, Kute T, Liu ET, Koerner F, et al. c-erB-2 expression and response to adjuvant therapy in women with node-positive early Breast cancer. N Engl J Med 1994;330(18):1260-1266.
10. Group TFAS. Benefit of a high-dose epirubicin regimen in adjuvant chemotherapy for node-positive breast cancer patients with poor prognostic factors: 5-year follow-up results of French Adjuvant Study Group 05 randomized trial. J Clin Oncol 2001;19(3):602-11.
11. Petit T, Borel C, Ghnassia J-P, Rodier J-F, Escande A, Mors R, et al. Chemotherapy response of breast cancer depends on HER-status and antracycline dose intensity in the neoadjuvant setting. Clin Cancer Res 2001;7:1577-1581.
12. Norton L. Adjuvant breast cancer therapy: Current status and future strategies - Growth kinetics and the improved drug therapy of breast cancer. Semin Oncol 1999;26(1):1-4.
13. Citron ML, Berry DA, Cirrincione C, Hudis C, Winer EP, Gradishar WJ, et al. Randomized trial of dose-dense versus conventionally scheduled and sequential versus concurrent combination chemotherapy as postoperative adjuvant treatment of node-positive primary breast cancer: First report of intergroup trial C9741/cancer and leukemia group B trial 9741. J. Clin. Oncol. 2003;21(8):1431-1439.
14. von Minckwitz G, Raab G, Caputo A, Schutte M, Hilfrich F, Blohmer JU, et al. Doxorubicin with cyclophosphamide followed by docetaxel every 21 days compared with doxorubicin and docetaxel every 14 days as preoperative treatment in operable breast cancer: The GEPARDUO Study of the German Breast Group. J. Clin. Oncol. 2005;23(12):2676-2685.
15. Gundersen S, Kvinnsland S, Klepp O, Kvaløy S, Lund E, Høst H. Weekly adriamycin versus VAC in advanced breast cancer. A randomized trial. Eur J Cancer Clin. Oncol. 1986;22:1431-1434.
16. Slamon D, Eiermann W, Robert N, Pienkowski T, Martin M, Pawlicki M, et al. Phase III randomized trial comparing doxorubicin and cyclophosphamide followed by docetaxel (AC (R) T) with doxorubicin and

cyclophosphamide followed by docetaxel and trastuzumab (AC (R) TH) with docetaxel, carboplatin and trastuzumab (TCH) in HER2 positive early breast cancer patients: BCIRG 006 study. Breast Cancer Res Treat 2005;94:S5-S5.
17. Press MF, Bernstein L, Sauter G, Zhou JY, Eiermann W, Pienkowski T, et al. Topoisomerase II-alpha gene amplification as a predictor of responsiveness to anthracycline-containing chemotherapy in the Cancer International Research Group 006 clinical trial of trastuzumab (herceptin) in the adjuvant setting. Breast Cancer Res Treat 2005;94:S54-S54.
18. Bastholt L, Dalmark M, Gjedde SB, Pfeiffer P, Pedersen D, Sandberg E, et al. Dose-response relationship of epirubicin in the treatment of postmenopausal patients with metastatic breast cancer: A randomized study of epirubicin at four different dose levels performed by the Danish Breast Cancer Cooperative Group. J Clin Oncol 1996;14:1146-1155.
19. Nabholtz JM, Falkson C, Campos D, Szanto J, Martin M, Chan S, et al. Docetaxel and doxorubicin compared with doxorubicin and cyclophosphamide as first-line chemotherapy for metastatic breast cancer: Results of a randomized, multicenter, phase III trial. J. Clin. Oncol. 2003;21(6):968-975.
20. Mamounas EP, Bryant J, Leinbersky B, Fehrenbacher L, Sedlacek SM, Fisher B, et al. Paclitaxel after doxorubicin plus cyclophosphamide as adjuvant chemotherapy for node-positive breast cancer: Results from NSABP B-28. J. Clin. Oncol. 2005;23(16):3686-3696.
21. Smith RE, Bryant J, DeCillis A, Anderson S. Acute myeloid leukemia and myelodysplastic syndrome after doxorubicin-cyclophosphamide adjuvant therapy for operable breast cancer: The national surgical adjuvant breast and bowel project experience. J Clin Oncol 2003;21(7):1195-1204.
22. Paridaens R, Biganzoli L, Bruning P, Klijn JGM, Gamucci T, Houston S, et al. Paclitaxel versus doxorubicin as first-line single-agent chemotherapy for metastatic breast cancer: A European organization for research and treatment of cancer randomized study with cross-over. J Clin Oncol 2000;18(4):724-733.
23. Pivot X, Asmar L, Hortobagyi GN. The efficacy of chemotherapy with docetaxel and paclitaxel in anthracycline-resistant breast cancer (Review). Int J Oncol 1999;15(2):381-386.
24. Joensuu H, Holli K, Heikkinen M, Suonio E, Aro AR, Hietanen P, et al. Combination chemotherapy versus single-agent therapy as first- and second-line treatment in metastatic breast cancer: A prospective randomized trial. J Clin Oncol 1998;16(12):3720-3730.
25. Sledge GW, Neuberg D, Bernardo P, Ingle JN, Martino S, Rowinsky EK, et al. Phase III trial of doxorubicin, paclitaxel, and the combination of doxorubicin and paclitaxel as front-line chemotherapy for metastatic breast cancer: An intergroup trial (E1193). Journal of Clinical Oncology 2003;21(4):588-592.
26. Ejlertsen B, Mouridsen HT, Langkjer ST, Andersen J, Sjostrom J, Kjaer M. Phase III study of intravenous vinorelbine in combination with epirubicin versus epirubicin alone in patients with advanced breast cancer: A Scandinavian breast group trial (SBG9403). Journal of Clinical Oncology 2004;22(12):2313-2320.
27. Norris B, Pritchard KI, James K, Myles J, Bennett K, Marlin S, et al. Phase III comparative study of vinorelbine combined with doxorubicin versus doxorubicin alone in disseminated metastatic/recurrent breast cancer: National Cancer Institute of Canada Clinical Trials Group Study MA8. Journal of Clinical Oncology 2000;18(12):2385-2394.
28. Paik SM, Bryant J, Park CH, Fisher B, TanChiu E, Hyams D, et al. erbB-2 and response to doxorubicin in patients with axillary lymph node-positive, hormone receptor-negative breast cancer. J Natl Cancer Inst 1998;90(18):1361-1370.
29. Järvinen TAH, Tanner M, Rantanen V, Barlund M, Borg A, Grenman S, et al. Amplification and deletion of topoisomerase II alpha associate with ErbB-2 amplification and affect sensitivity to topoisomerase II inhibitor doxorubicin in breast cancer. Am J Pathol 2000;156(3):839-847.
30. Di Leo A, Gancberg D, Larsimont D, Tanner M, Jarvinen T, Rouas G, et al. HER-2 amplification and topoisomerase II alpha gene aberrations as predictive markers in node-positive breast cancer patients randomly treated either with an anthracycline-based therapy or with cyclophosphamide, methotrexate, and 5-fluorouracil. Clin Cancer Res 2002;8(5):1107-1116.
31. Cardoso F, Durbecq V, Larsimont D, Paesmans M, Leroy JY, Rouas G, et al. Correlation between complete response to anthracycline-based chemotherapy and topoisomerase II-alpha gene amplification and protein overexpression in locally advanced/metastatic breast cancer. International Journal of Oncology 2004;24(1):201-209.
32. Coon JS, Marcus E, Gupta-Burt S, Seelig S, Jacobson K, Chen S, et al. Amplification and overexpression of topoisomerase IIalpha predict response to anthracycline-based therapy in locally advanced breast cancer. Clin Cancer Res 2002;8(4):1061-7.
33. Knoop AS, Knudsen H, Balslev E, Rasmussen BB, Overgaard J, Nielsen KV, et al. Retrospective analysis of topoisomerase IIa amplifications and deletions as predictive markers in primary breast cancer patients randomly assigned to cyclophosphamide, methotrexate, and fluorouracil or cyclophosphamide, epirubicin, and fluorouracil: Danish Breast Cancer Cooperative Group. J Clin Oncol 2005;23(30):7483-90.

34. Geisler S, Lønning PE, Aas T, Johnsen H, Fluge O, Haugen DF, et al. Influence of TP53 gene alterations and c-erbB-2 expression on the response to treatment with doxorubicin in locally advanced breast cancer. Cancer Res 2001;61(6):2505-2512.
35. Kandioler-Eckersberger D, Ludwig C, Rudas M, Kappel S, Janschek E, Wenzel C, et al. TP53 mutation and p53 overexpression for prediction of response to neoadjuvant treatment in breast cancer patients. Clin Cancer Res 2000;6:50-56.
36. Lønning PE. Genes causing inherited cancer as beacons identifying the mechanisms of chemoresistance. Trends Mol Med 2004;10:113-118.
37. Chang J, Wooten E, Tsimelzon A, Hilsenbeck S, Gutierrez M, Elledge R, et al. Gene expression profiling for the prediction of therapeutic response to docetaxel in patients with breast cancer. Lancet 2003;362:362-69.
38. Ayers M, Symmans WF, Stec J, Damokosh AI, Clark E, Hess K, et al. Gene expression profiles predict complete pathologic response to neoadjuvant paclitaxel and fluorouracil, doxorubicin, and cyclophosphamide chemotherapy in breast cancer. J Clin Oncol 2004;22(12):2284-2293.
39. Hannemann J, Oosterkamp HM, Bosch CAJ, Velds A, Wessels LFA, Loo C, et al. Changes in gene expression associated with response to neoadjuvant chemotherapy in breast cancer. J Clin Oncol 2005;23(15):3331-3342.
40. Gianni L, Zambetti M, Clark K, Baker J, Cronin M, Wu J, et al. Gene expression profiles in paraffin-embedded core biopsy tissue predict response to chemotherapy in women with locally advanced breast cancer. J Clin Oncol 2005;23(29):7265-77.
41. Iwao-Koizumi K, Matoba R, Ueno N, Kim SJ, Ando A, Miyoshi Y, et al. Prediction of docetaxel response in human breast cancer by gene expression profiling. J Clin Oncol 2005;23(3):422-431.
42. Lønning PE, Sørlie T, Børresen-Dale A-L. Genomics in breast cancer - therapeutic implications? Nature Clin Pract Oncol 2005;2:26-33.
43. Houghton J, Stoicov C, Nomura S, Rogers AB, Carlson J, Li H, et al. Gastric cancer originating from bone marrow-derived cells. Science 2004;306(5701):1568-71.
44. Weimann JM, Charlton CA, Brazelton TR, Hackman RC, Blau HM. Contribution of transplanted bone marrow cells to Purkinje neurons in human adult brains. Proc Natl Acad Sci U S A 2003;100(4):2088-93.
45. Shackleton M, Vaillant F, Simpson KJ, Stingl J, Smyth GK, Asselin-Labat ML, et al. Generation of a functional mammary gland from a single stem cell. Nature 2006;439(7072):84-88.
46. Wang Y, Huso DL, Harrington J, Kellner J, Jeong DK, Turney J, et al. Outgrowth of a transformed cell population derived from normal human BM mesenchymal stem cell culture. Cytotherapy 2005;7(6):509-19.
47. Pittenger MF, Mackay AM, Beck SC, Jaiswal RK, Douglas R, Mosca JD, et al. Multilineage potential of adult human mesenchymal stem cells. Science 1999;284(5411):143-7.
48. Kaplan RN, Riba RD, Zacharoulis S, Bramley AH, Vincent L, Costa C, et al. VEGFR1-positive haematopoietic bone marrow progenitors initiate the pre-metastatic niche. Nature 2005;438(7069):820-7.
49. Rubio D, Garcia-Castro J, Martin MC, de la Fuente R, Cigudosa JC, Lloyd AC, et al. Spontaneous human adult stem cell transformation. Cancer Res 2005;65(8):3035-9.
50. Lønning P. Study of suboptimum treatment response: lessons from breast cancer. Lancet Oncol 2003;4:177-185.
51. Joensuu H, Kellokumpu-Lehtinen P, Bono P, Alanko T, Kataja V, Asola R, et al. Adjuvant docetaxel or vinorelbine with or without trastuzumab for breast cancer. N Engl J Med 2006;354(8):809-820.
52. Romond EH, Perez EA, Bryant J, Suman VJ, Geyer CE, Jr., Davidson NE, et al. Trastuzumab plus adjuvant chemotherapy for operable HER2-positive breast cancer. N Engl J Med 2005;353(16):1673-84.
53. Piccart-Gebhart MJ, Procter M, Leyland-Jones B, Goldhirsch A, Untch M, Smith I, et al. Trastuzumab after adjuvant chemotherapy in HER2-positive breast cancer. N Engl J Med 2005;353(16):1659-72.
54. Therasse P, Arbuck SG, Eisenhauer E, Wanders J, Kaplan RS, Rubinstein L, et al. New guidelines to evaluate the response to treatment in solid tumors. J Natl Cancer Inst 2000;92:205-216.
55. Geisler S, Børresen-Dale A-L, Johnsen H, Aas T, Geisler J, Akslen LA, et al. TP53 gene mutations predict the response to neoadjuvant treatment with FUMI in locally advanced breast cancer. Clin Cancer Res 2003;9:5582-5588.
56. Staalesen V, Falck J, Geisler S, Bartkova J, Borresen-Dale AL, Lukas J, et al. Alternative splicing and mutation status of CHEK2 in stage III breast cancer. Oncogene 2004;23(52):8535-8544.
