# Supplementary material for: Clonal evolution in primary breast cancers under sequential epirubicin and docetaxel monotherapy
Source: Genome Med. 2022 Aug 11;14:86. doi: 10.1186/s13073-022-01090-2 (PMC9367103; doi:10.1186/s13073-022-01090-2)
Supplement: Supplementary file 3 — Additional file 3: Figure S1. CONSORT diagram for the Dose-Dense trial. Figure S2. Survival in the Dose-Dense trial after a minimum follow-up of five years or until death. Figure S3. Average sequencing coverage for each sample subject to whole exome sequencing (WES). Figure S4. Average sequencing coverage for each pretreatment tumor sample subject to amplicon-based sequencing. Figure S5. Oncoplot depicting the frequency of mutations in tumor samples undergoing whole exome sequencing (WES). Figure S6. Merged overview of copy number alterations (CNAs) in the sample set. Figure S7. Scatter plot showing Pearson correlation between copy number alterations (CNAs) and tumor mutation burden. Figure S8. Estimated contribution of mutational processes for each of the patients according to the classification by COSMIC. Figure S9. Graphical representation of clonal evolution from the pretreatment setting to post-epirubicin and post-docetaxel. Figure S10. Circos plots, coxcomb plots and heatmaps illustrating copy number logRs and mutation rVAFs pretreatment, post-epirubicin and post-docetaxel. [file 13073_2022_1090_MOESM3_ESM.docx]

**Supplementary Figures**

**
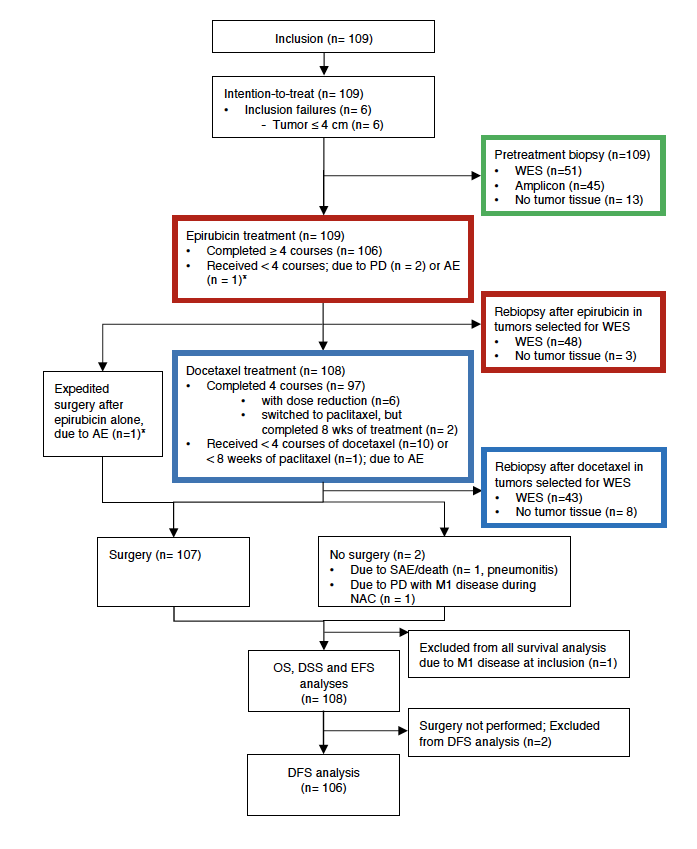
**

**Fig. S1**

CONSORT diagram for the Dose-Dense trial, illustrating patient numbers with respect to inclusion, treatment and molecular analyses.

**
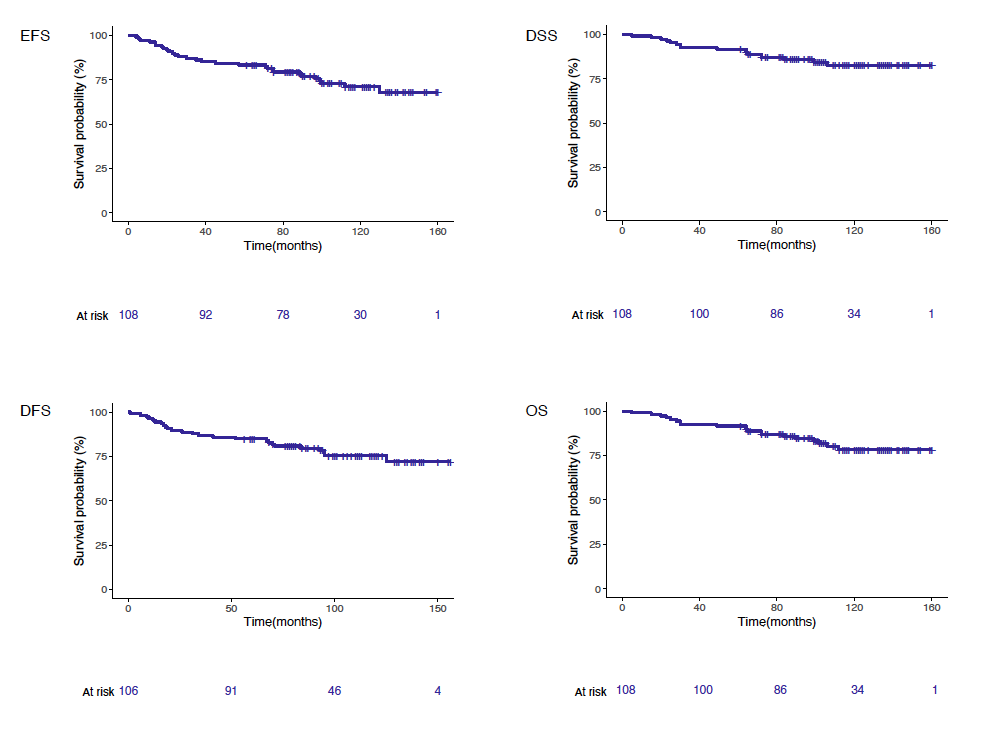
Fig. S2**

Event-free survival (EFS), disease-free survival (DFS), disease-specific survival (DSS) and overall survival (OS) in the Dose-Dense trial after a minimum follow-up of five years or until death.


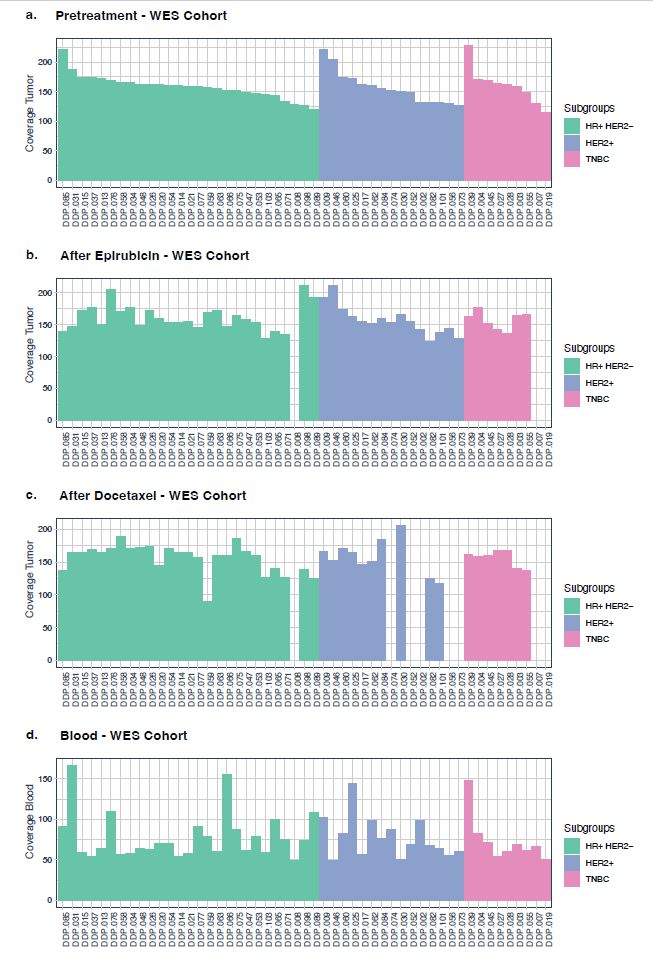


**Fig. S3**

Average sequencing coverage for each sample subject to whole exome sequencing (WES) **a)** tumor samples pretreatment, **b)**, tumor samples after epirubicin, **c)** tumor samples after docetaxel (at surgery). Green bars indicate samples from patients with hormone receptor positive (HR+) and HER2- tumors. Blue bars indicate samples from patients with HER2+ tumors. Pink bars indicate samples from patients with triple negative breast cancer (TNBC).


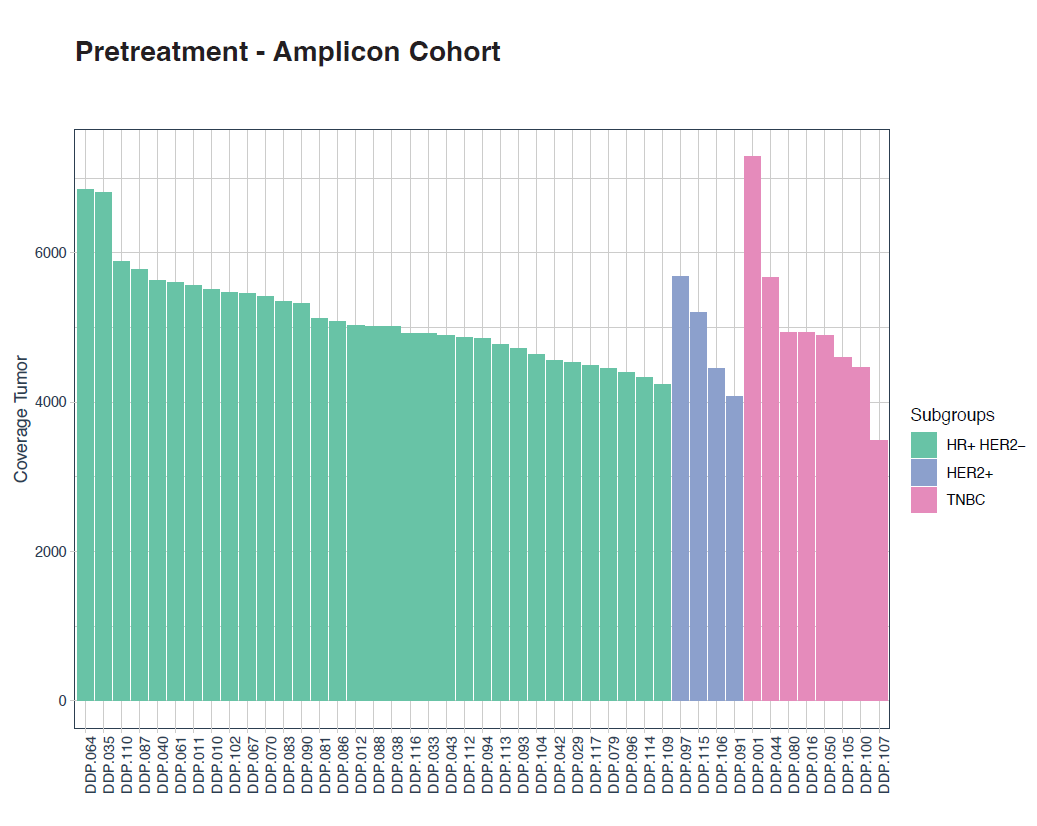


**Fig. S4**

Average sequencing coverage for each pretreatment tumor sample subject to amplicon-based sequencing. Green bars indicate samples from patients with hormone receptor positive (HR+) and HER2- tumors. Blue bars indicate samples from patients with HER2+ tumors. Pink bars indicate samples from patients with triple negative breast cancer (TNBC).

**Fig. S5**

Oncoplot depicting the frequency of mutations in tumor samples (n=51 patients) undergoing whole exome sequencing (WES) across the three different time-points (pretreatment (pre), post-epirubicin (Epi) and post-docetaxel (Doc)). The mutation list is sorted based on the responders and/or non-responders for each of the two treatments, and mutations are colored according to mutation type. Percentages on the right indicate the prevalence of mutations in each of the genes, among the 51 tumor samples analyzed. Each column represents one tumor per patient. Each row represents the three different timepoints (pretreatment, post-epirubicin and post-docetaxel). The panel under the oncoplot area is composed of three single stacked row heatmaps showing, samples across the two treatments with sufficient tumor cell fraction (TCF≥20%) as well as samples with insufficient tumor cell fraction (TCF<20%). White blocks indicate lack of data/biopsies. Responses listed are CR (complete response), PR, (partial response), SD (stable disease), according to the RECIST criteria, while PD (progressive disease) was classified according to the UICC criteria.

**
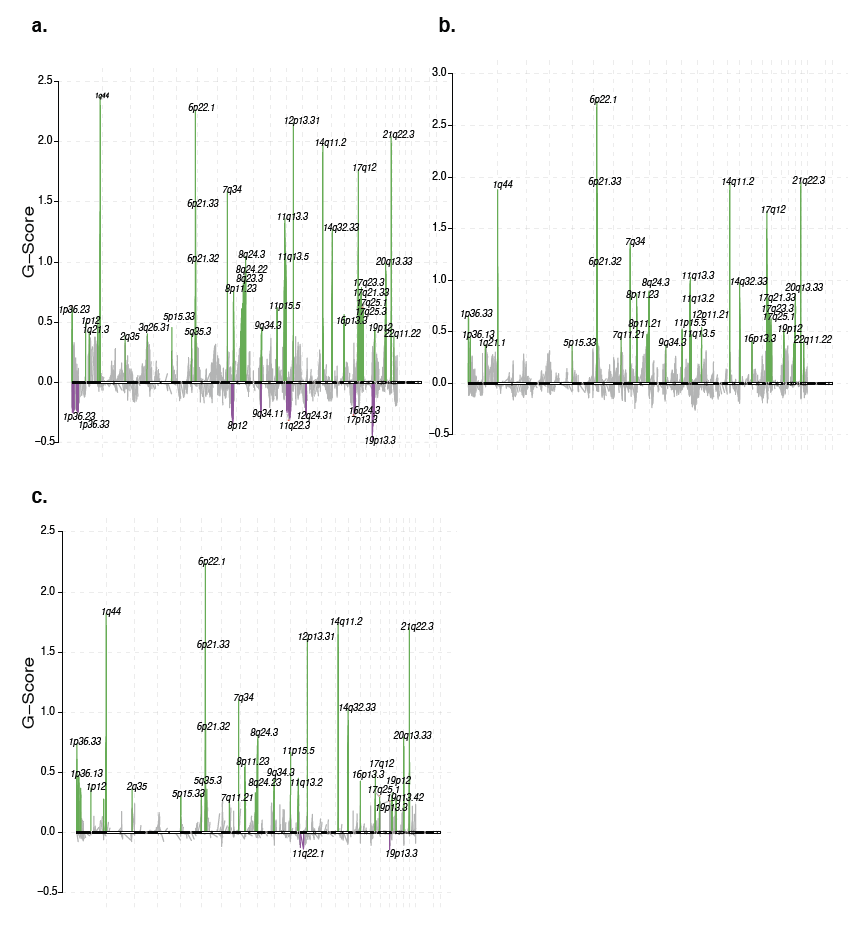
Fig. S6**

Merged overview of copy number alterations (CNAs) in the sample set (n=51 patients) at the three different timepoints **a)** pretreatment, **b)** post-epirubicin and **c)** post-docetaxel. Copy-number gains (green) and losses (purple) identified as significant within the data set using GISTIC2.0 (see Supplementary Methods), are highlighted.

**
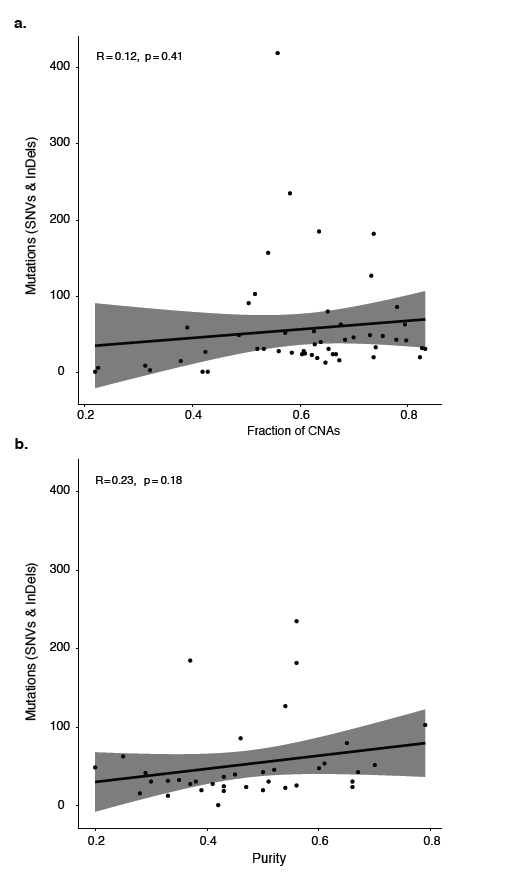
**

**Fig. S7**

Scatter plot showing Pearson correlation between copy number alterations (CNAs) and tumor mutation burden. X-axis; fraction of the exome affected by CNA (relative to ploidy in each sample). Y-axis: number of mutations (single nucleotide variants (SNVs) and indels) causing amino acid changes within the protein coding regions.

**
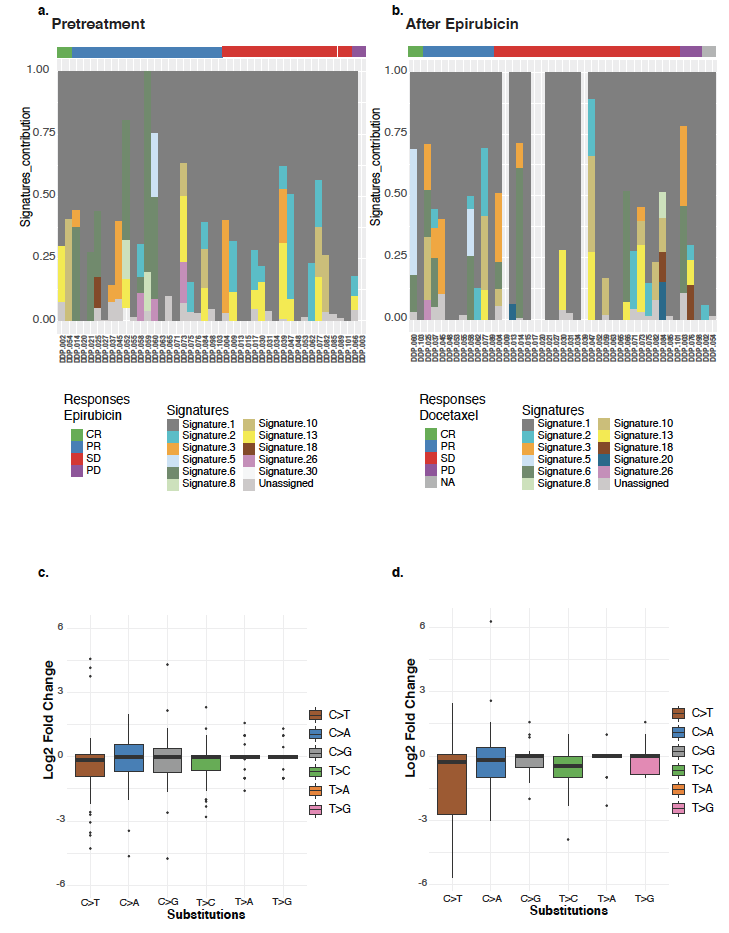
**

**Fig. S8**

Estimated contribution of mutational processes for each of the patients **a)** pretreatment and **b)** post-epirubicin, by fraction of mutations assigned to each mutational signature, according to the classification by COSMIC (only samples with total number of coding mutations >10 included. The number of mutations called post-docetaxel was too low for formal signature assessment). Samples are sorted based on a) response to epirubicin and b) response to docetaxel. Responses listed are CR (complete response), PR (partial response), SD (stable disease), according to the RECIST criteria, while PD (progressive disease) was classified according to the UICC criteria. Box-plots showing the relative fraction (ratio) of mutation types **c)** post-epirubicin versus pre-epirubicin and **d)** post-docetaxel treatment versus pre-docetaxel.


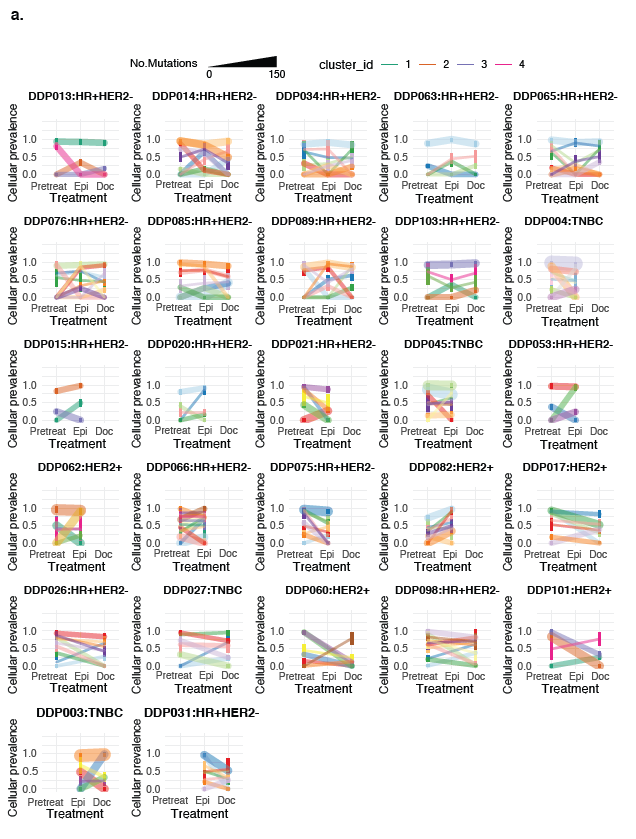


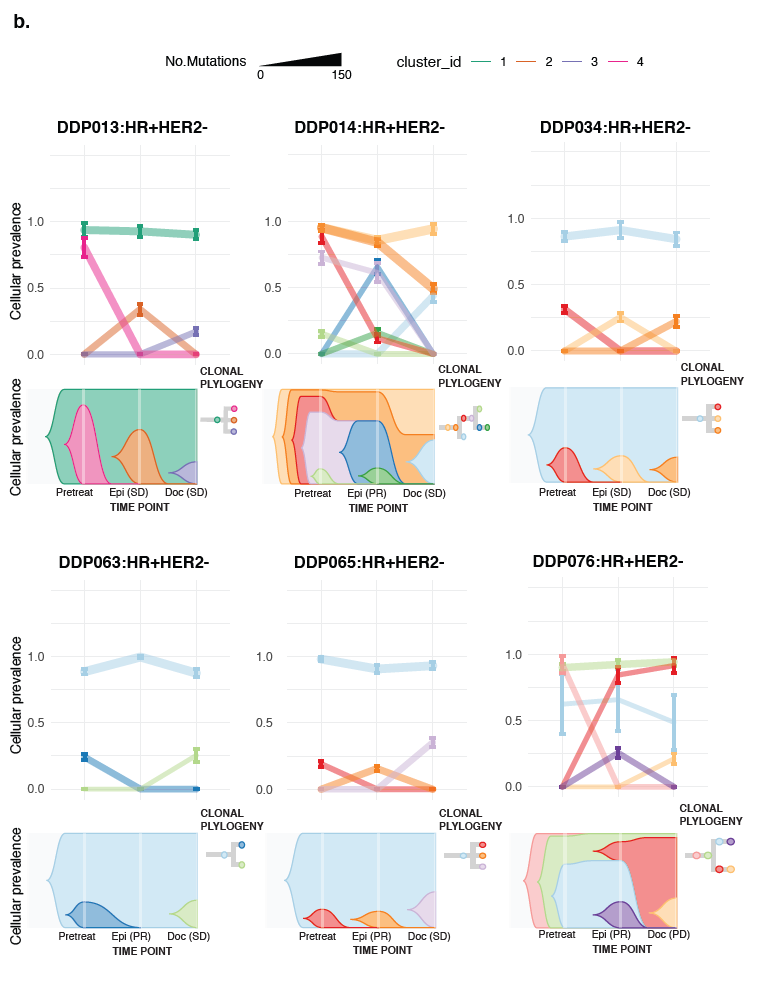


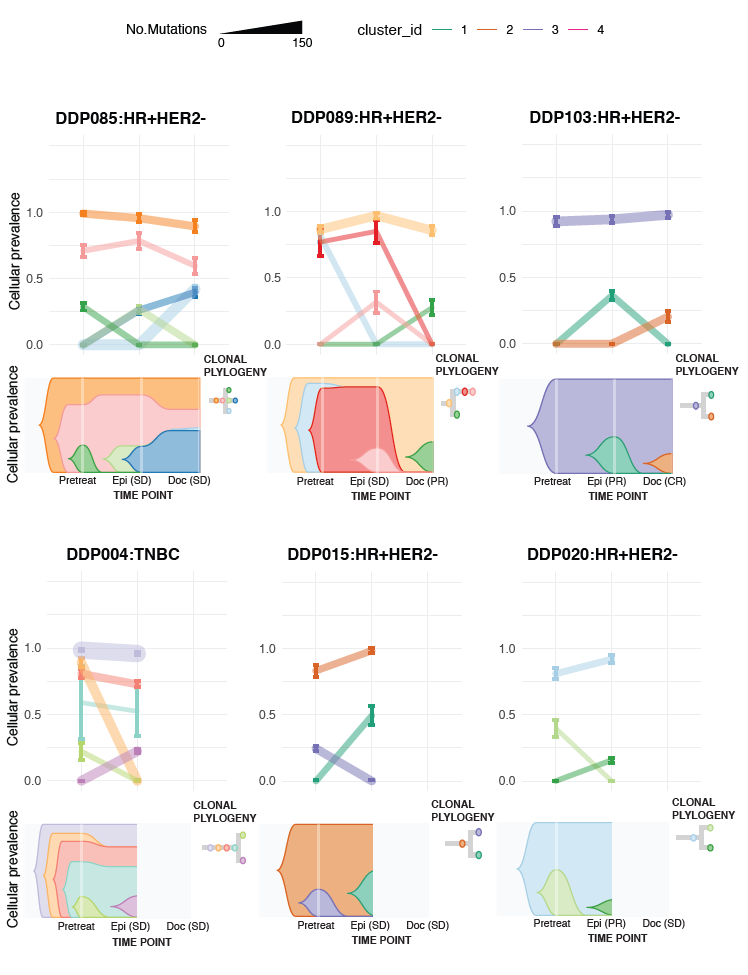


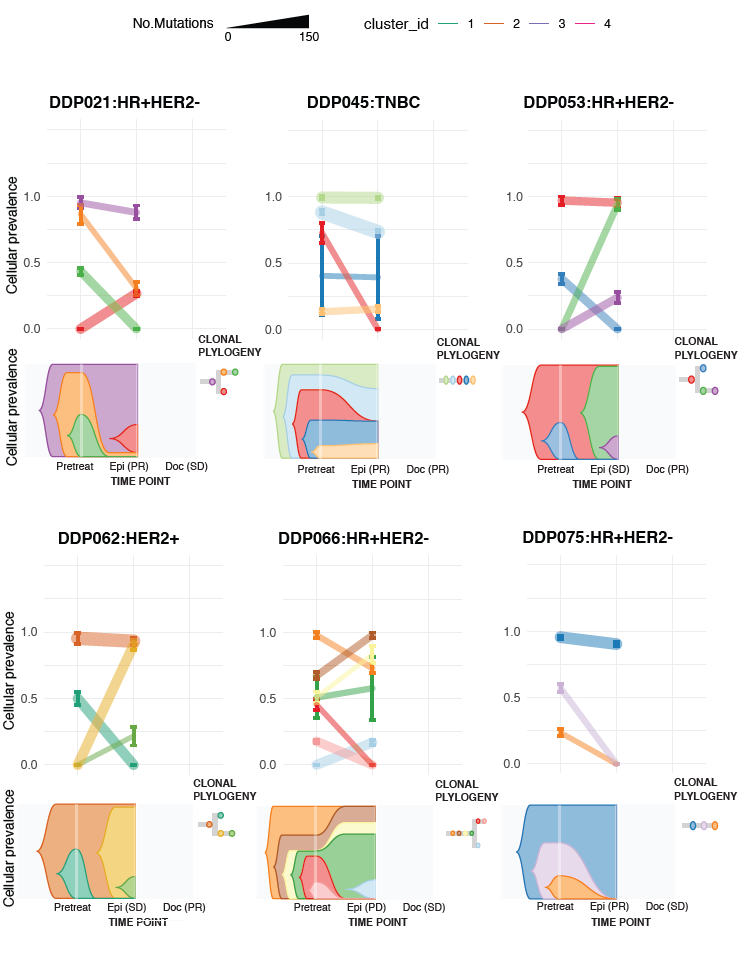


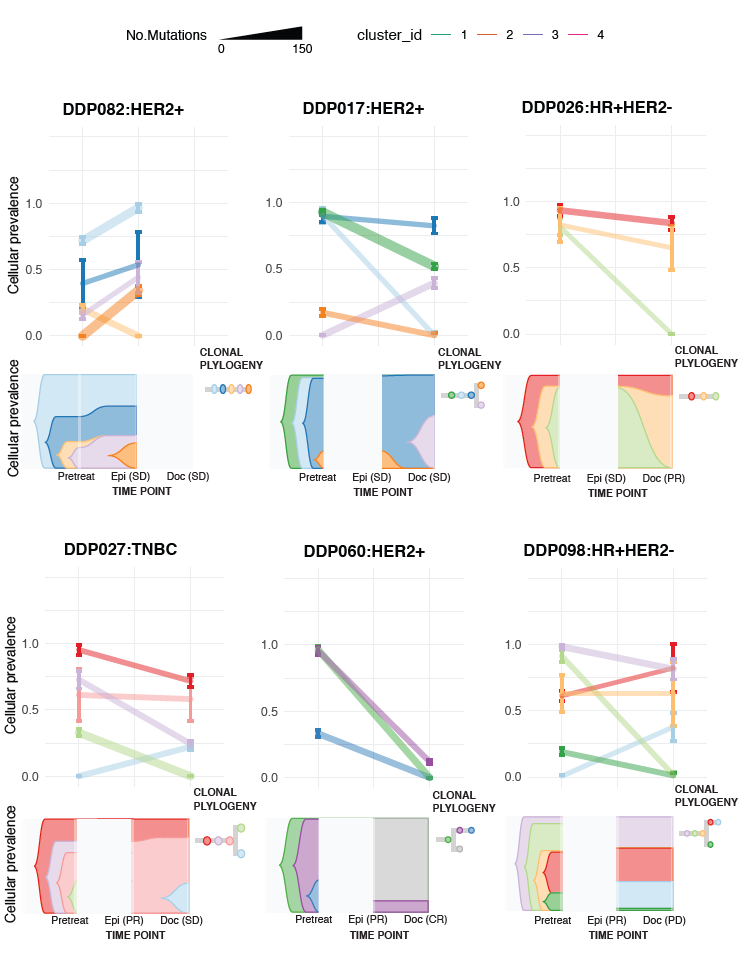


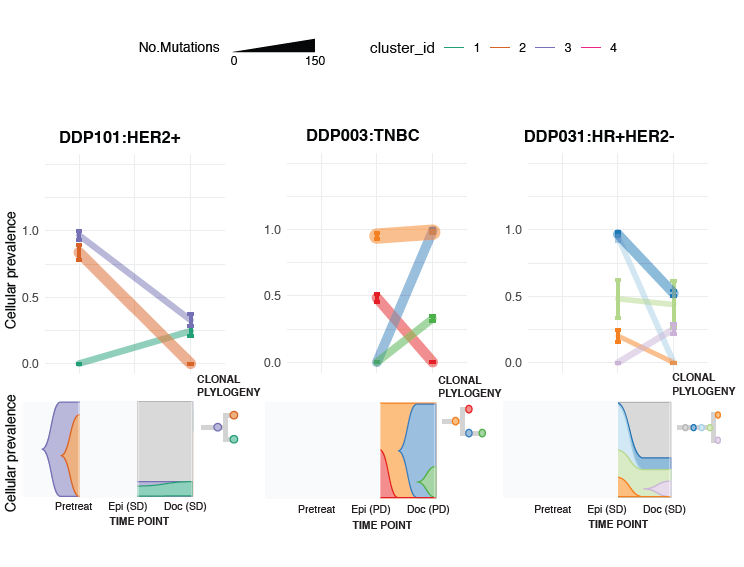


**Fig. S9**

Graphical representation of clonal evolution from the pretreatment setting to post-epirubicin and post-docetaxel*. **a)** Allelic prevalence of mutation clusters, based on variant allele frequencies (VAFs) for all mutations in samples from each patient. Clustering performed using the PyClone algorithm, with default parameters (see Supplementary Methods).

**b)** Allelic prevalence of mutation clusters (top panels), where “clusters” with one mutation have been merged to the nearest cluster based on z-score (range of -1,+1) probability. Subsequent clusters with less than three mutations have been removed for clarity. Tumor evolution diagrams (bottom panels), based on the "timescape algorithm" (see Supplementary Methods) using the cluster data in the top panels. Note that the illustrations do not distinguish between new subclones emerging, harboring all truncal mutations and those that have lost some truncal mutations. Each color represents an estimated subclone from the mutation clusters. The horizontal axis denotes three time points during tumor evolution: pretreatment, post-epirubicin and post-docetaxel (at surgery), with clinical response noted in brackets. Pretreat: pretreatment, Epi: post-epirubicin, Doc: post-docetaxel. *Only breast cancer biopsies with sufficient number of mutations and a tumor cell fraction ≥20% (n=27) were included.


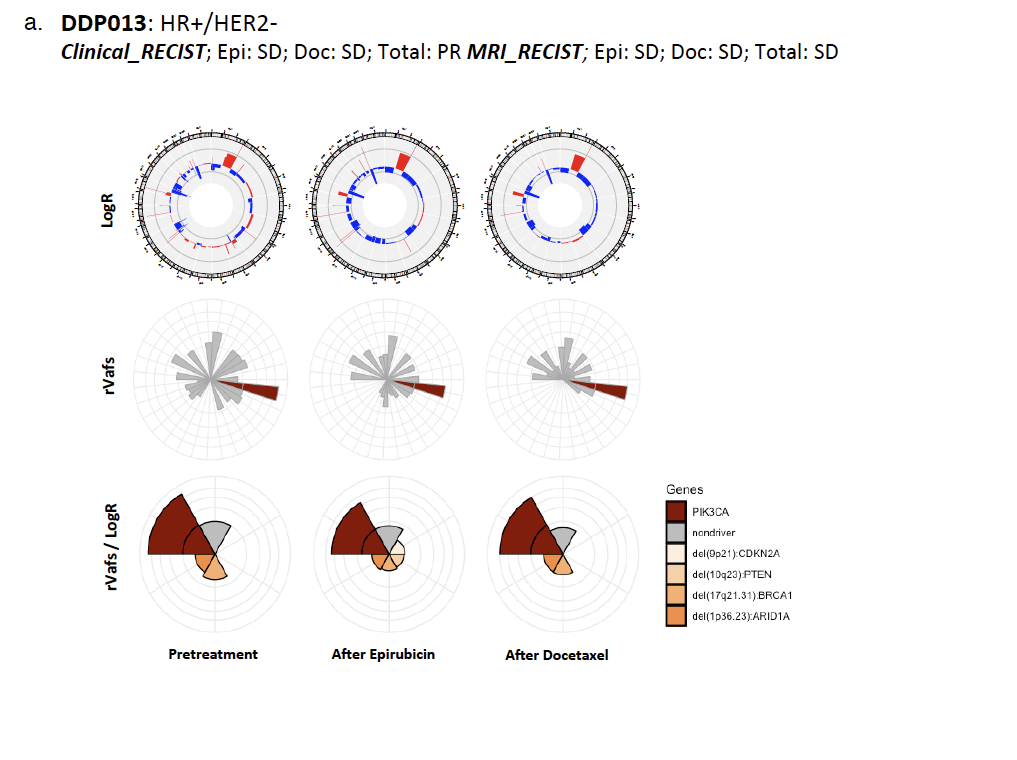


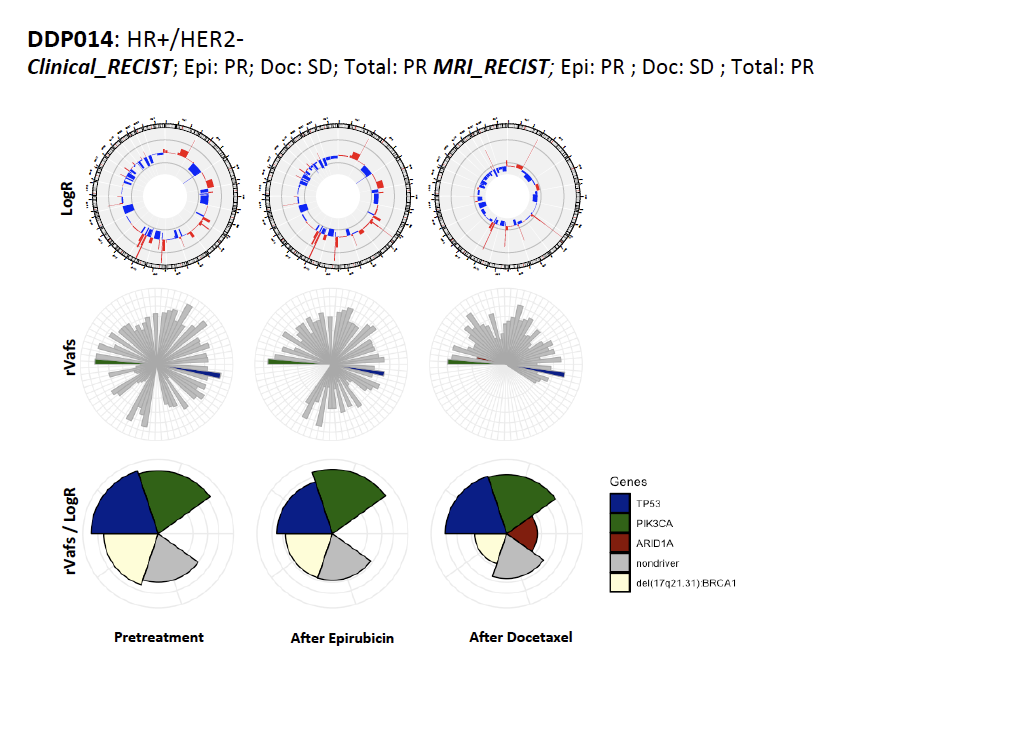


**
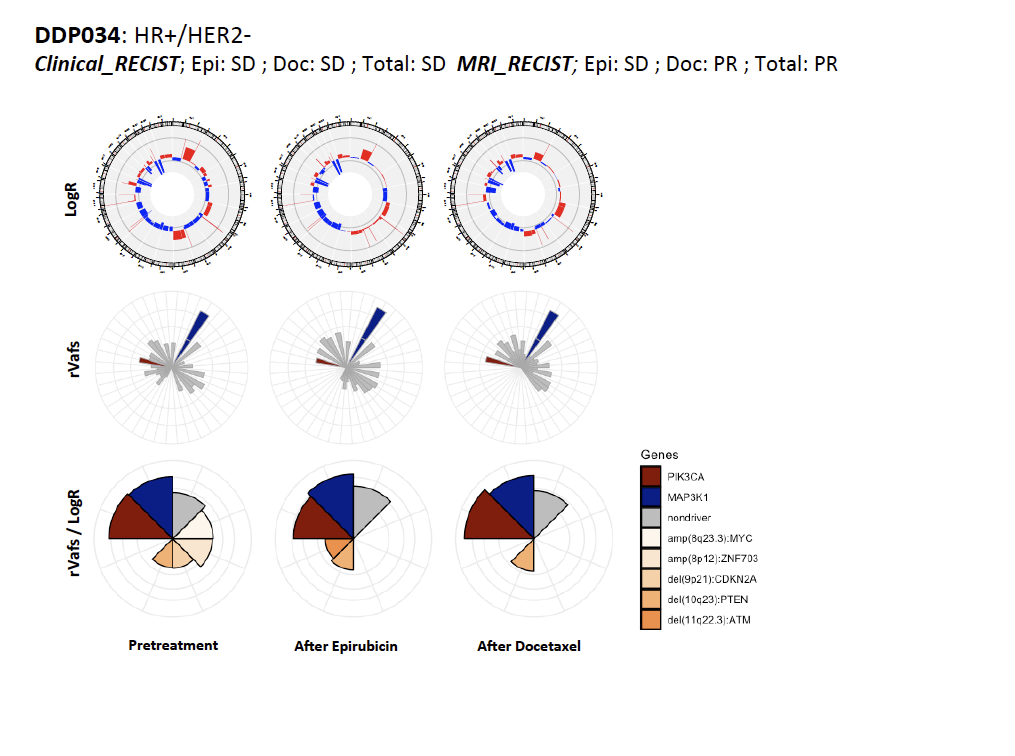
**

**
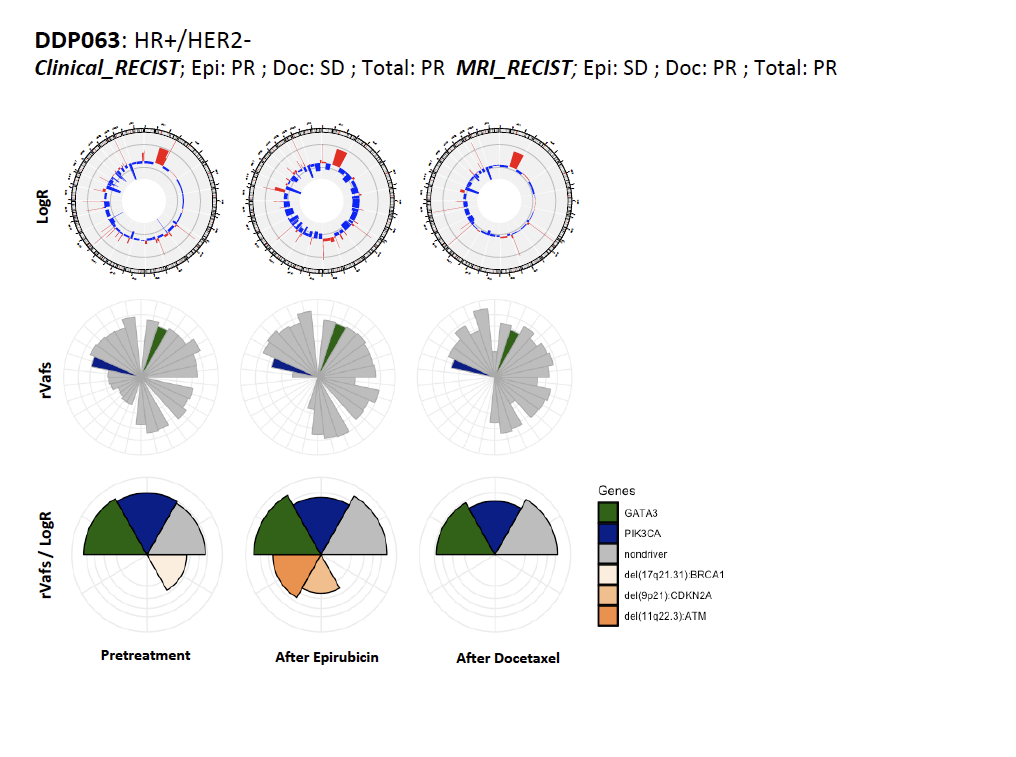
**

**
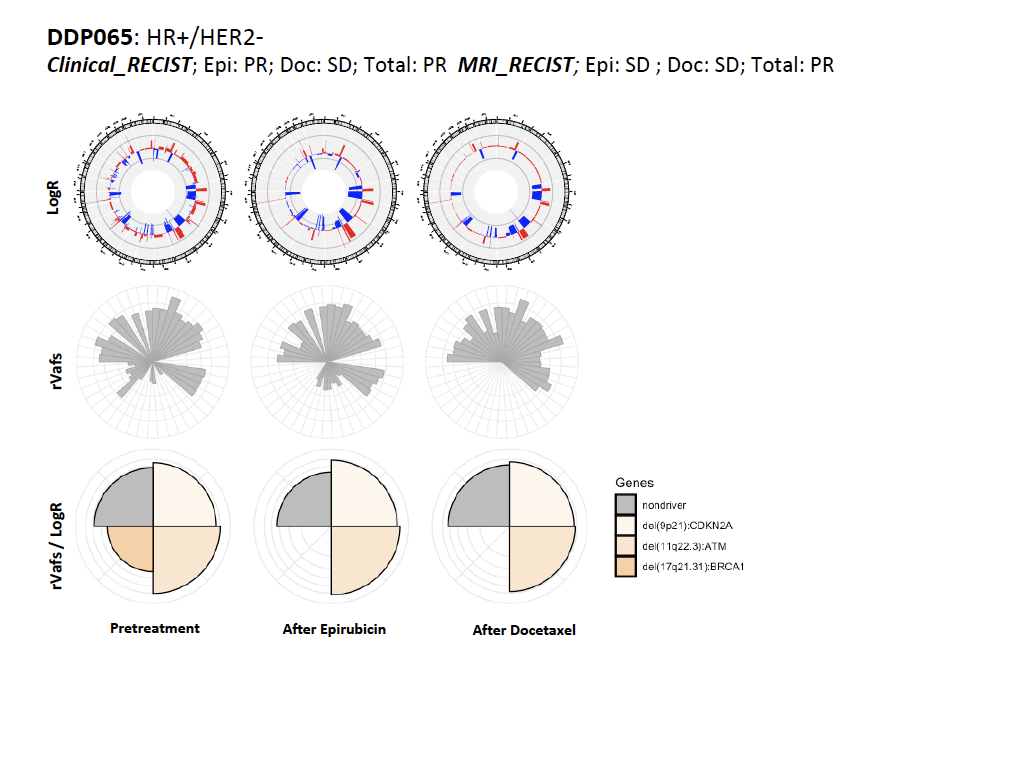
**

**
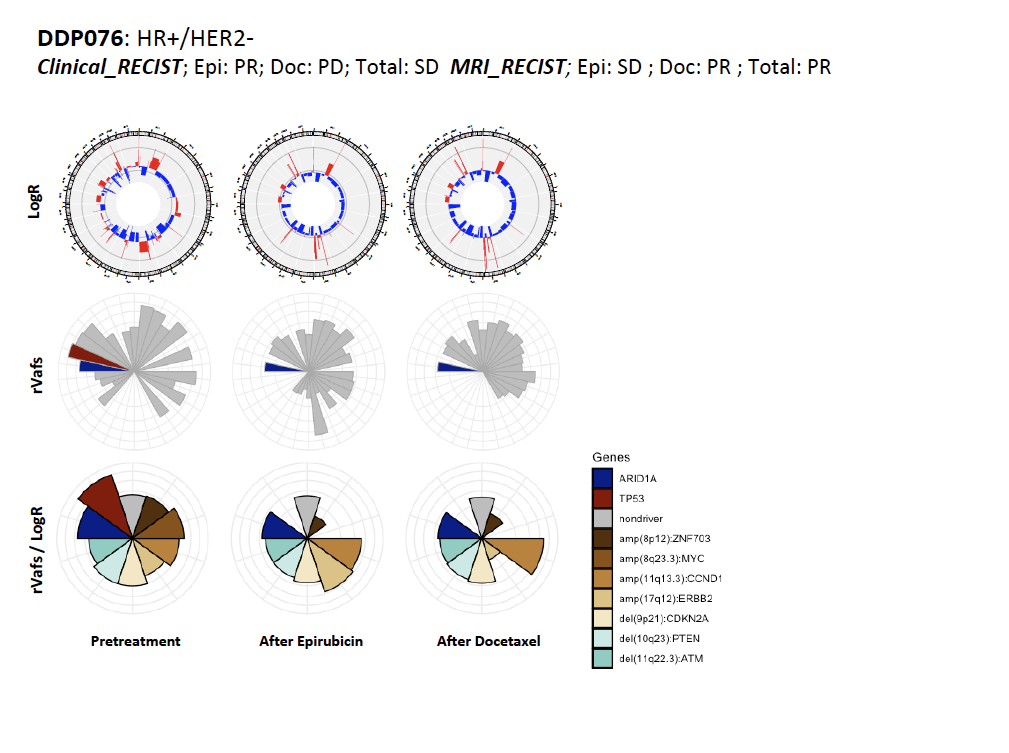
**

**
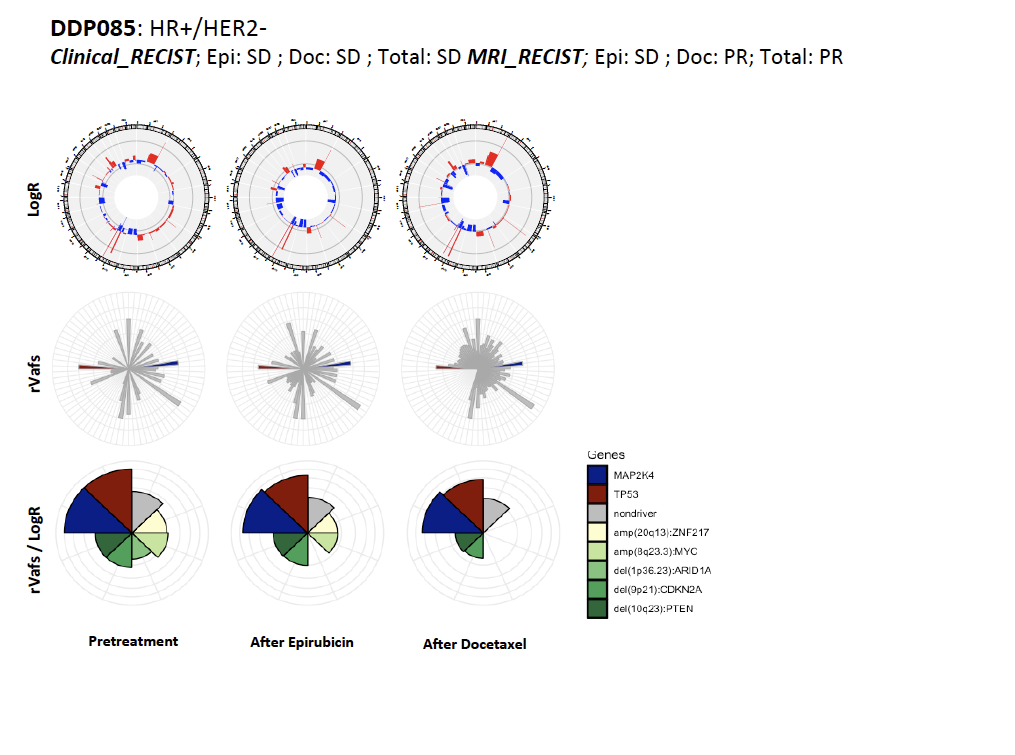
**

**
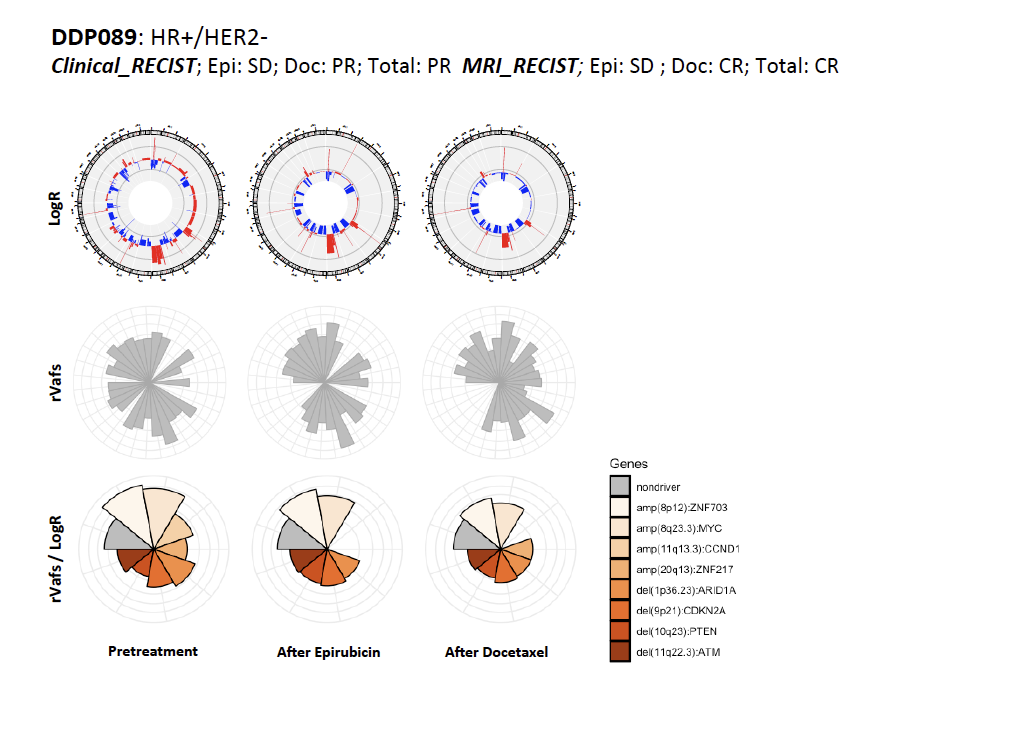
**

**
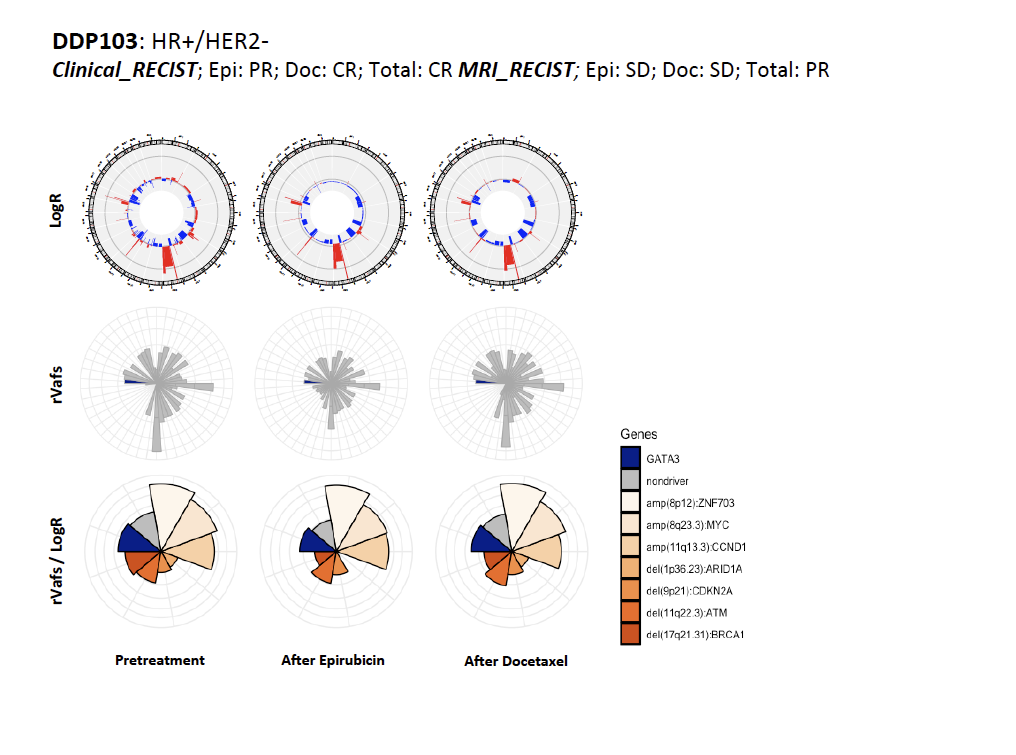

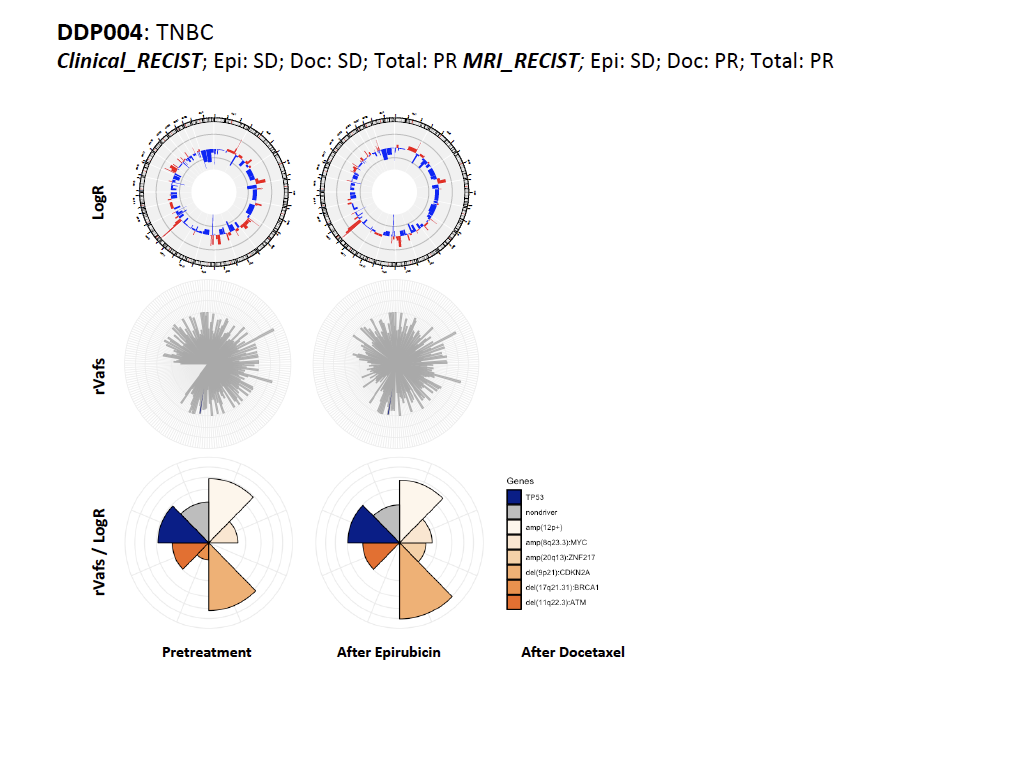
**

**
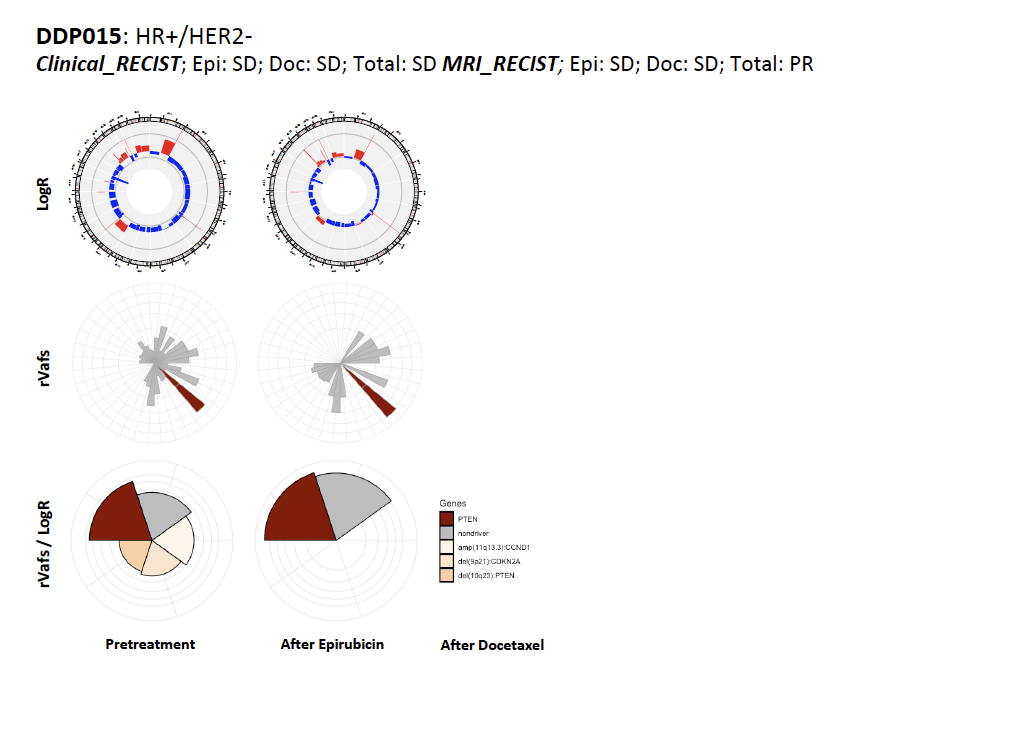
**

**
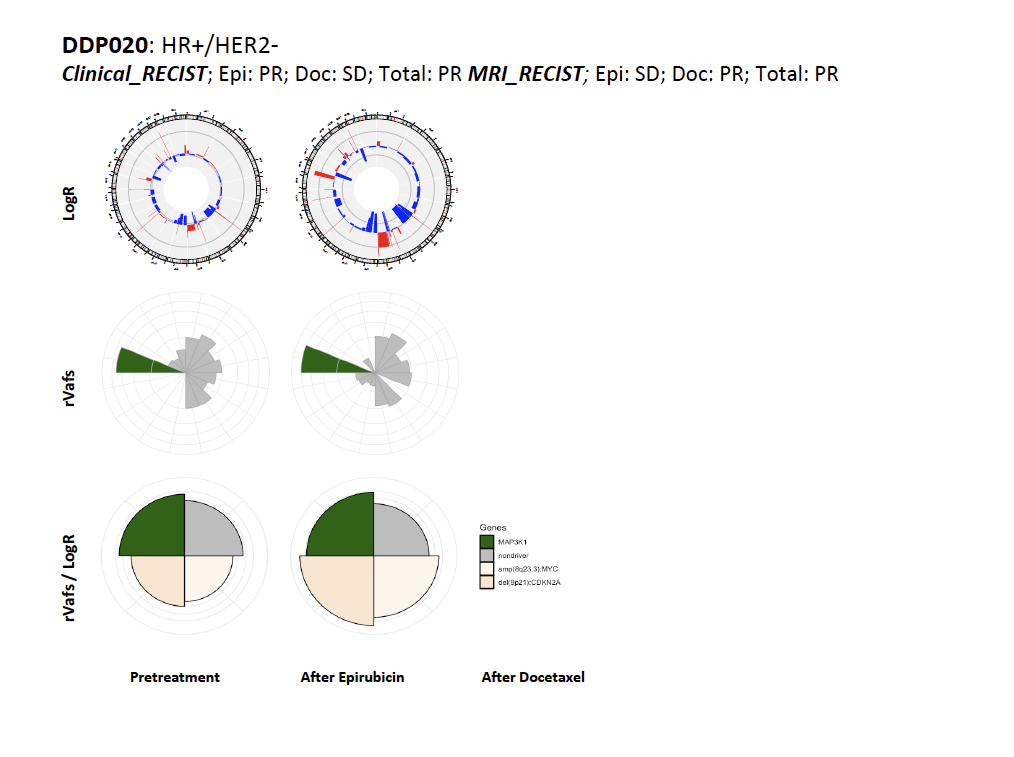
**

**
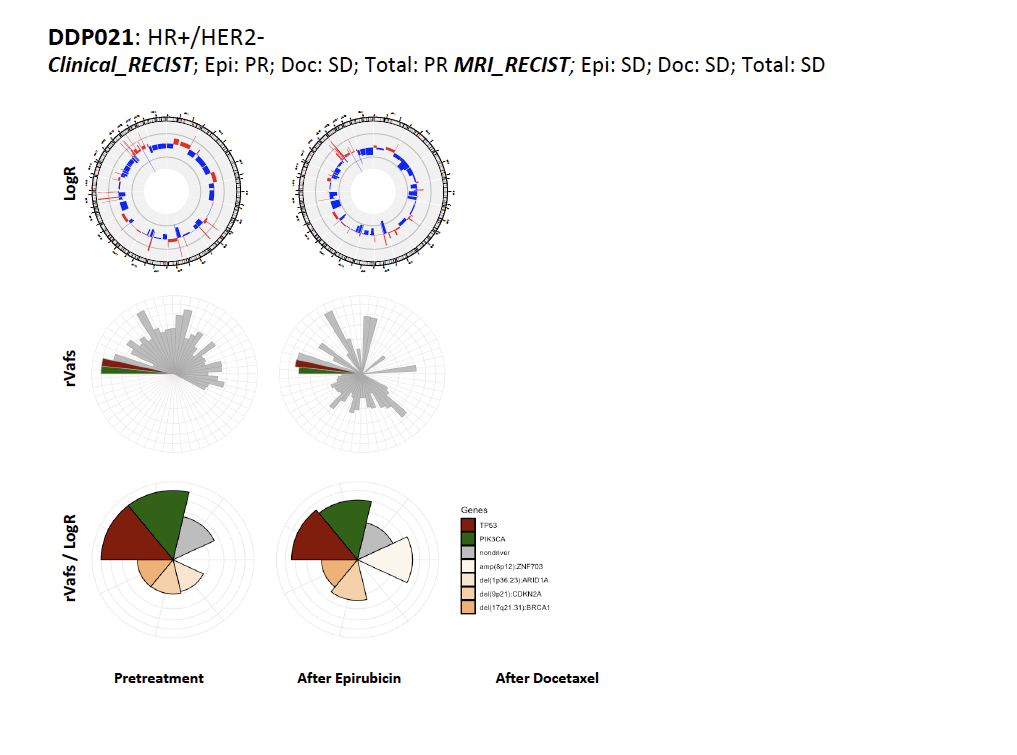
**

**
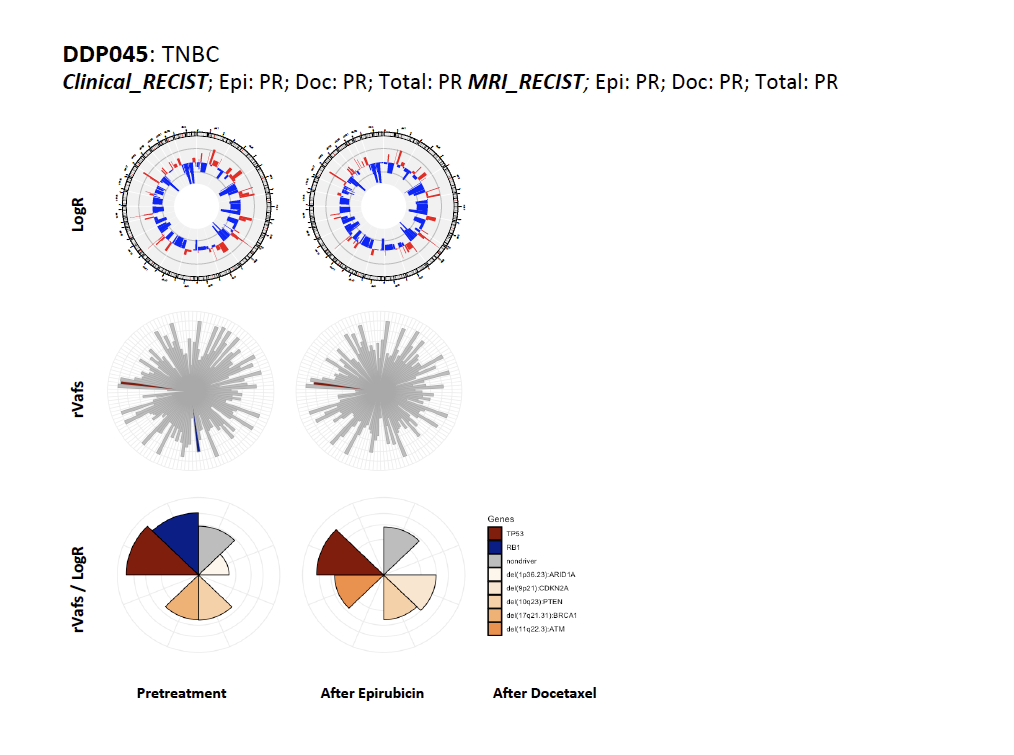
**

**
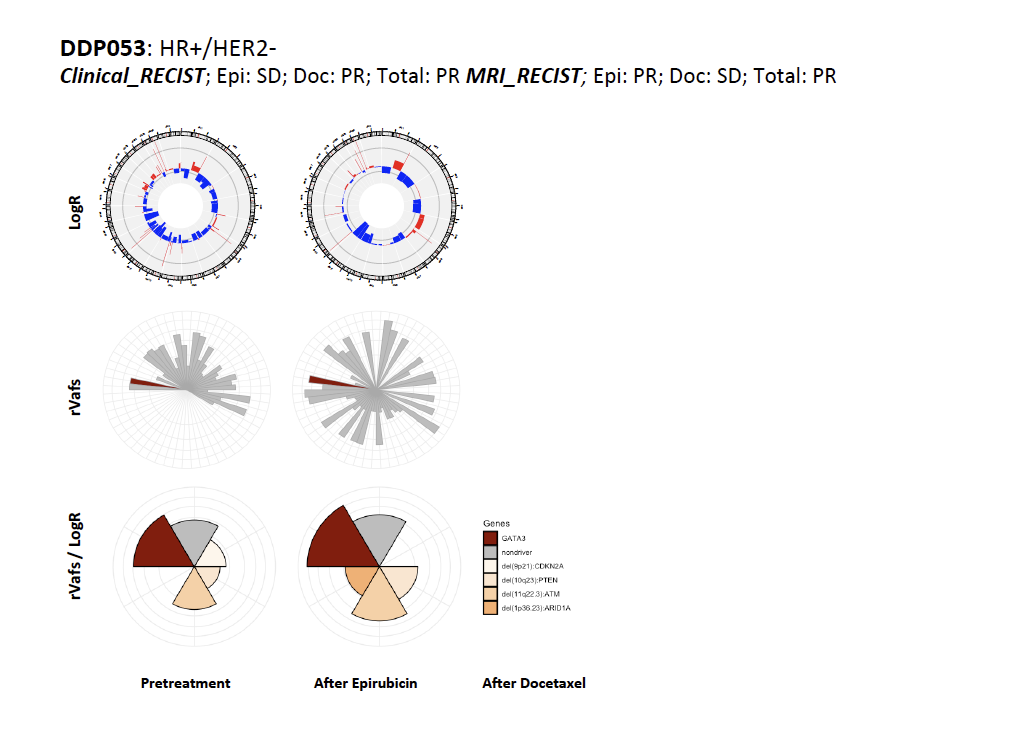

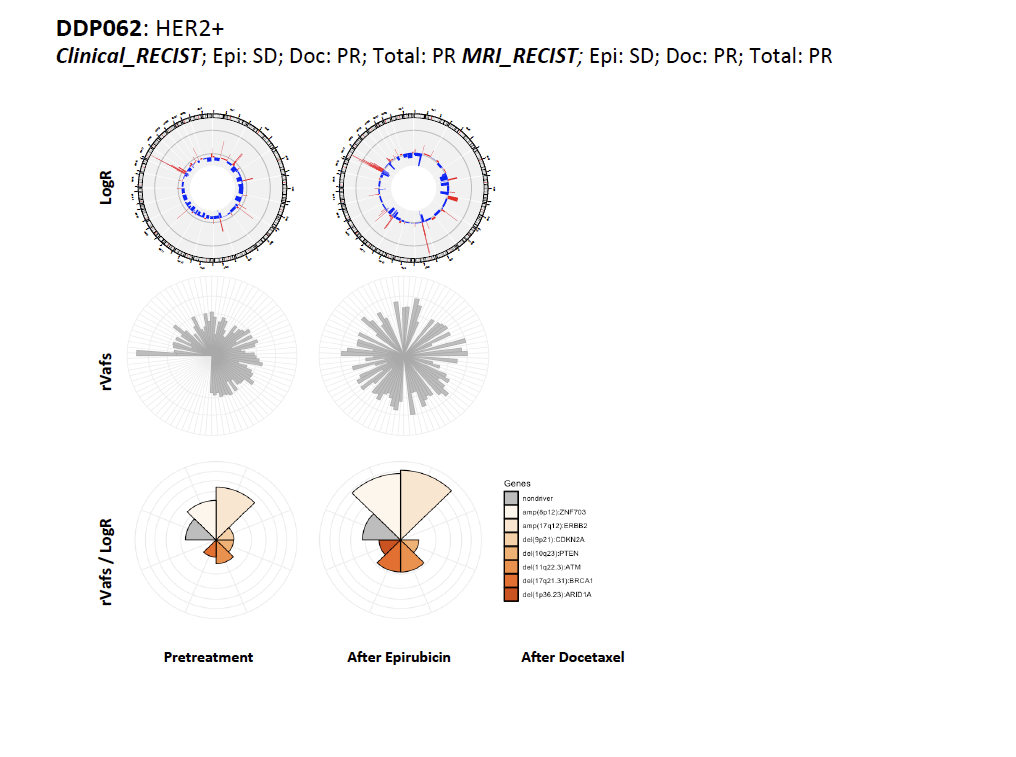
**

**
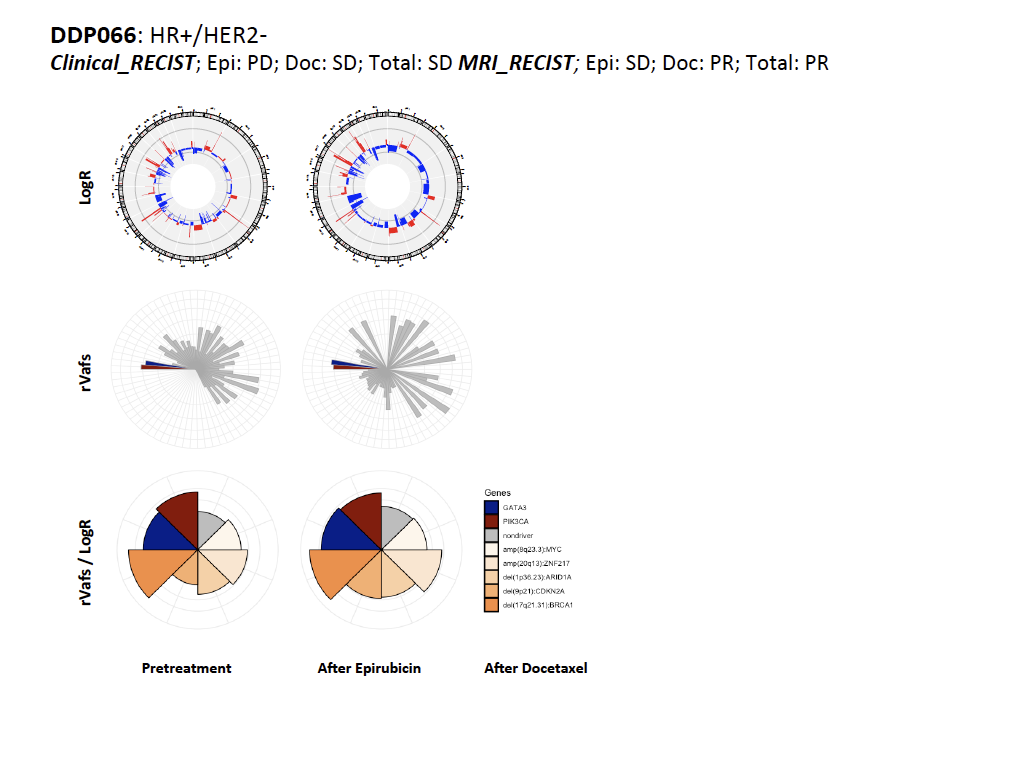
**

**
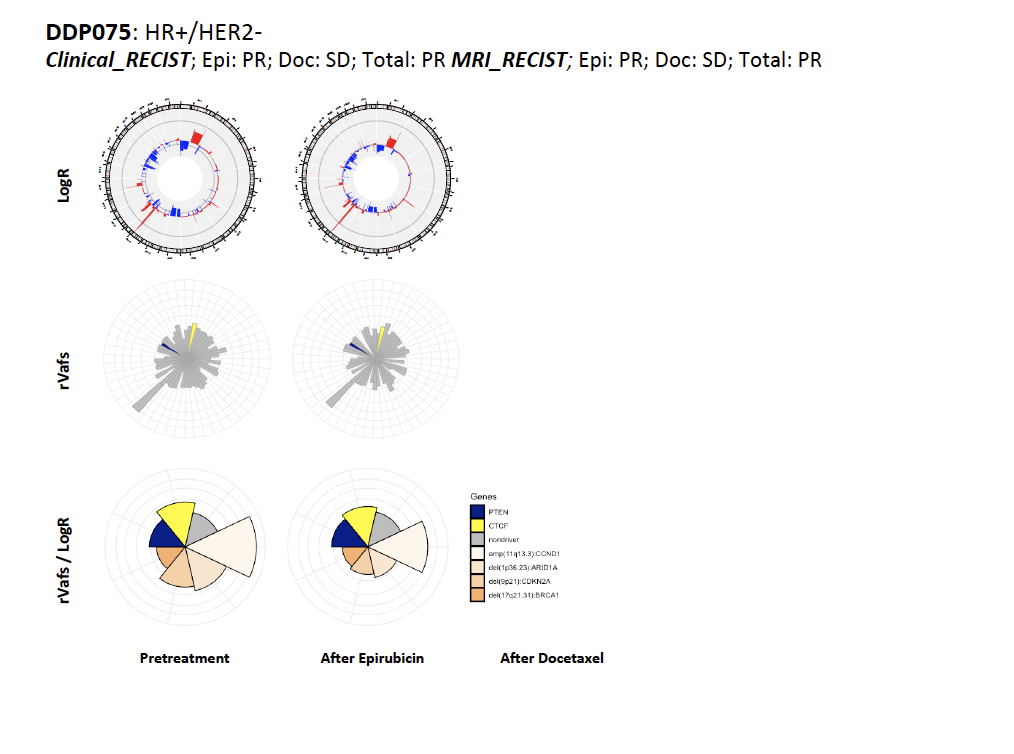
**

**
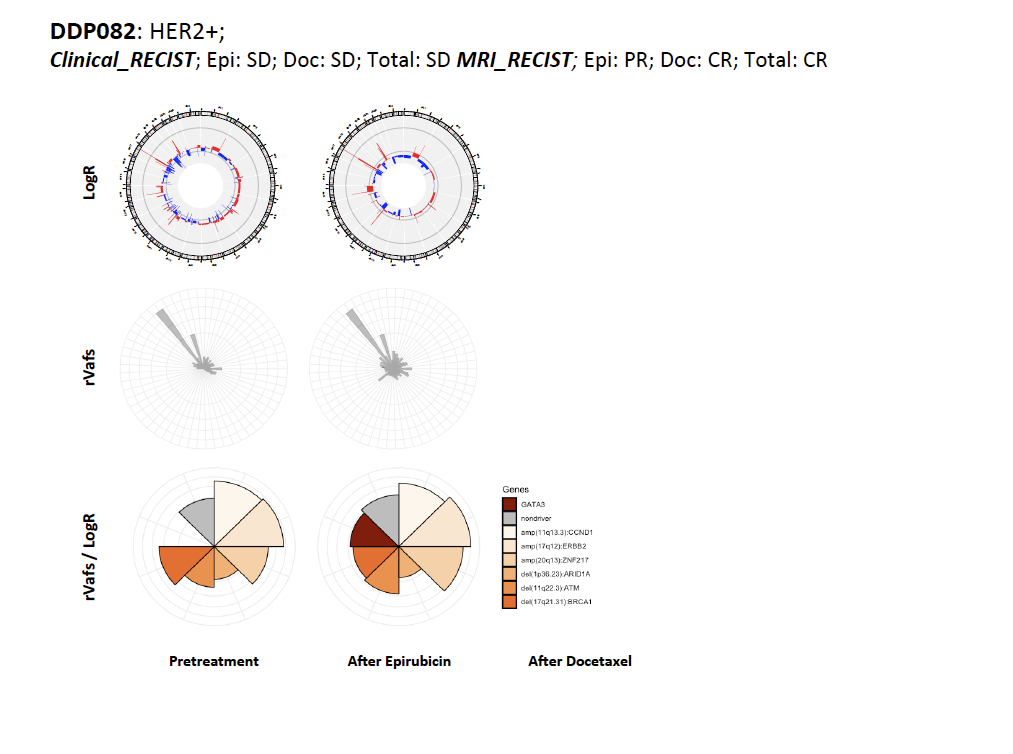
**

**
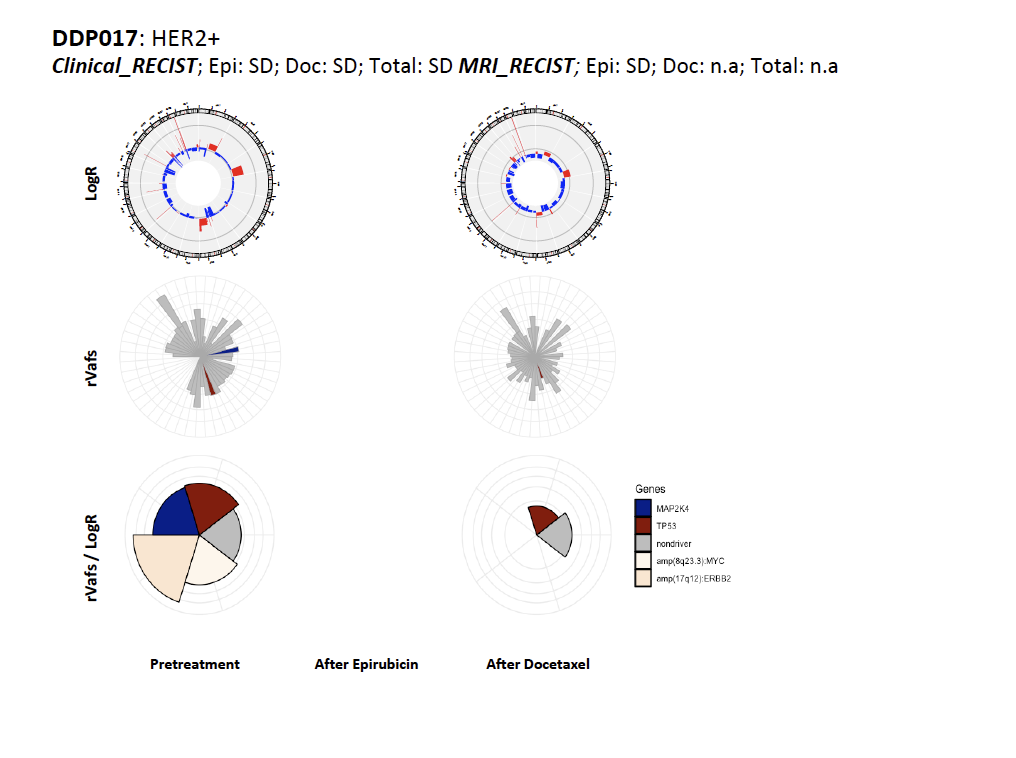
**

**
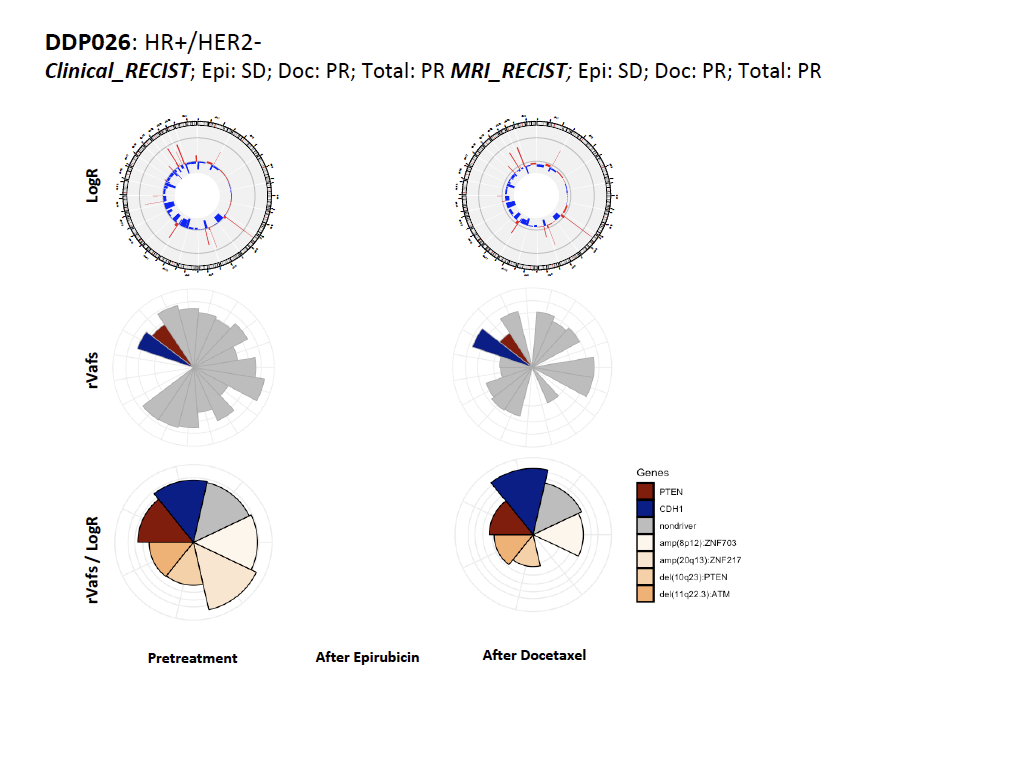

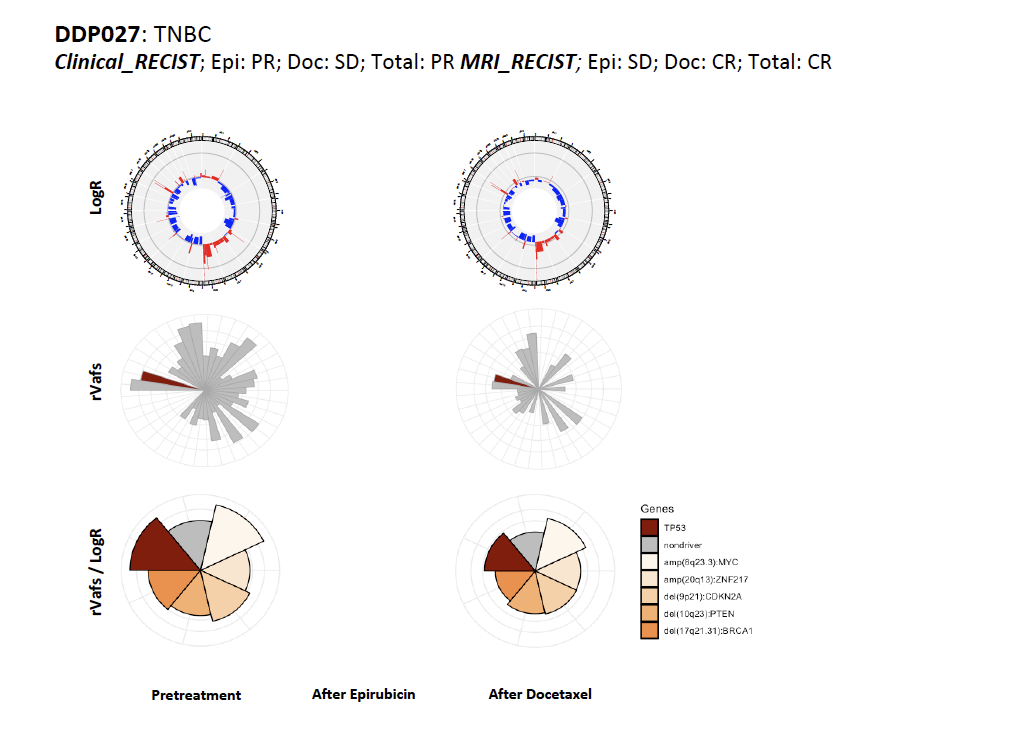
**

**
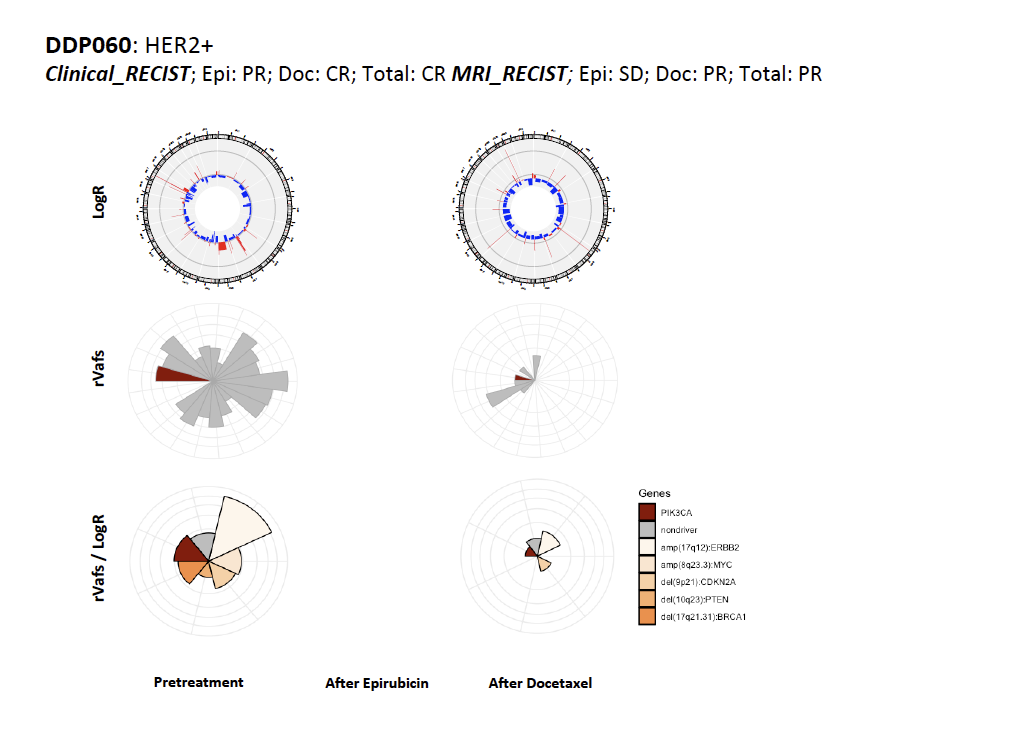
**

**
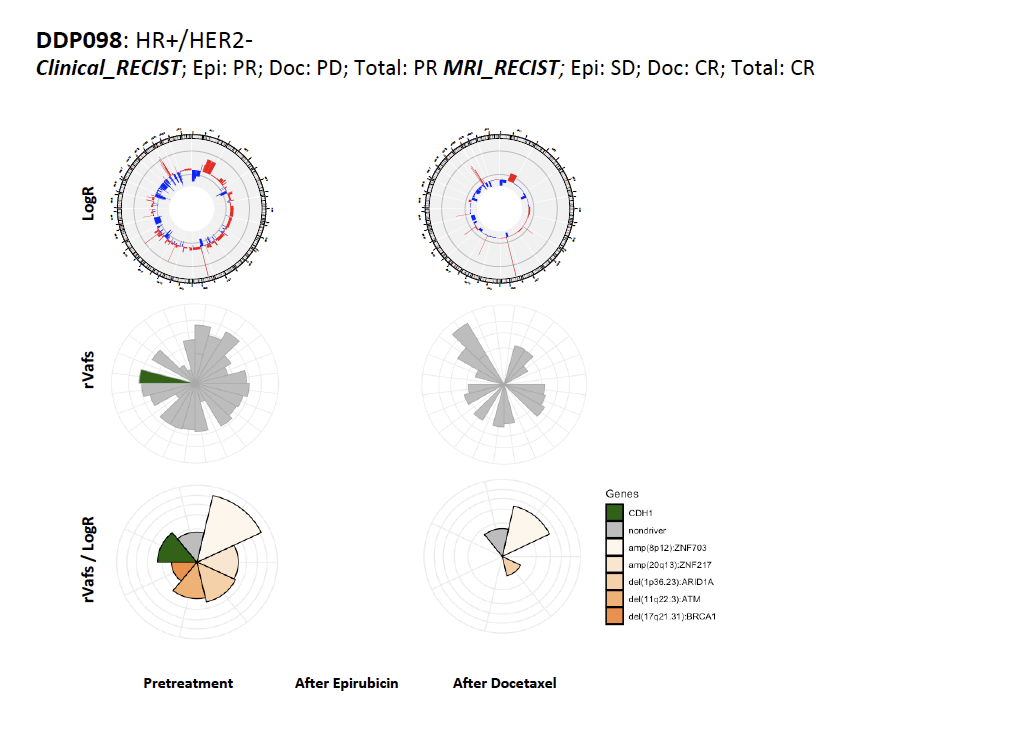
**

**
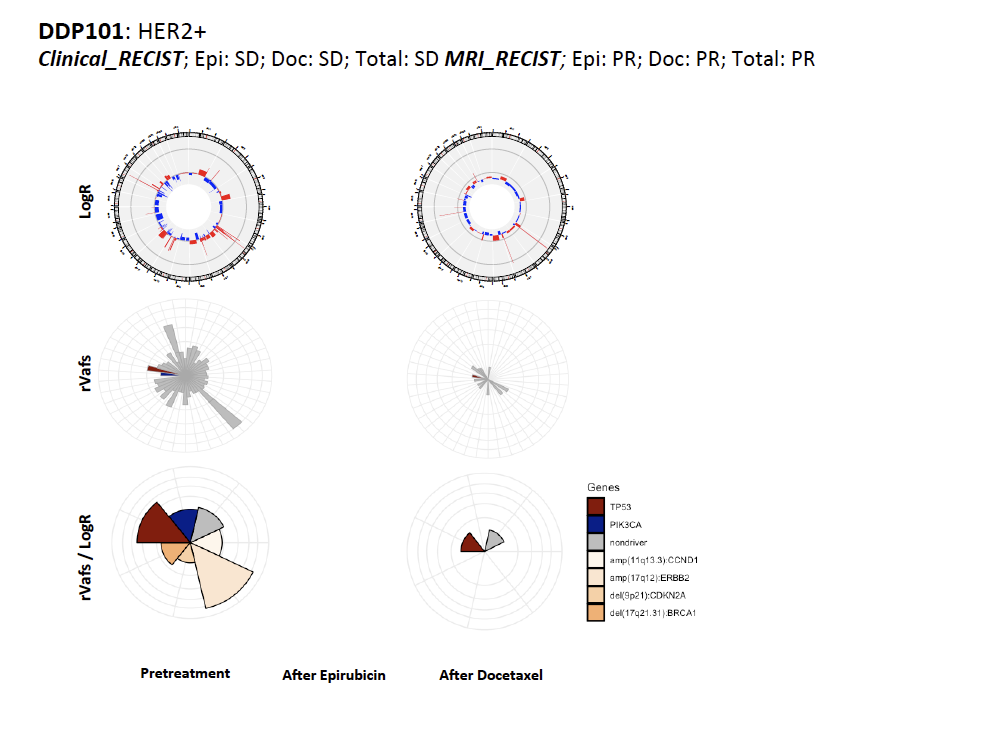
**

**
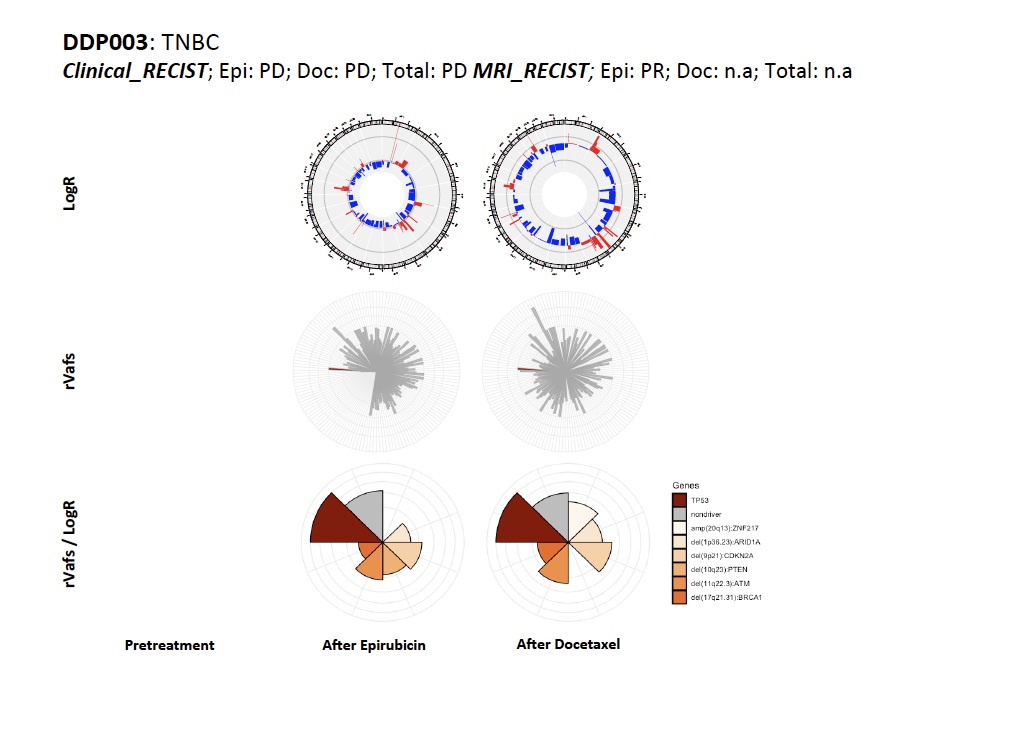
**

**
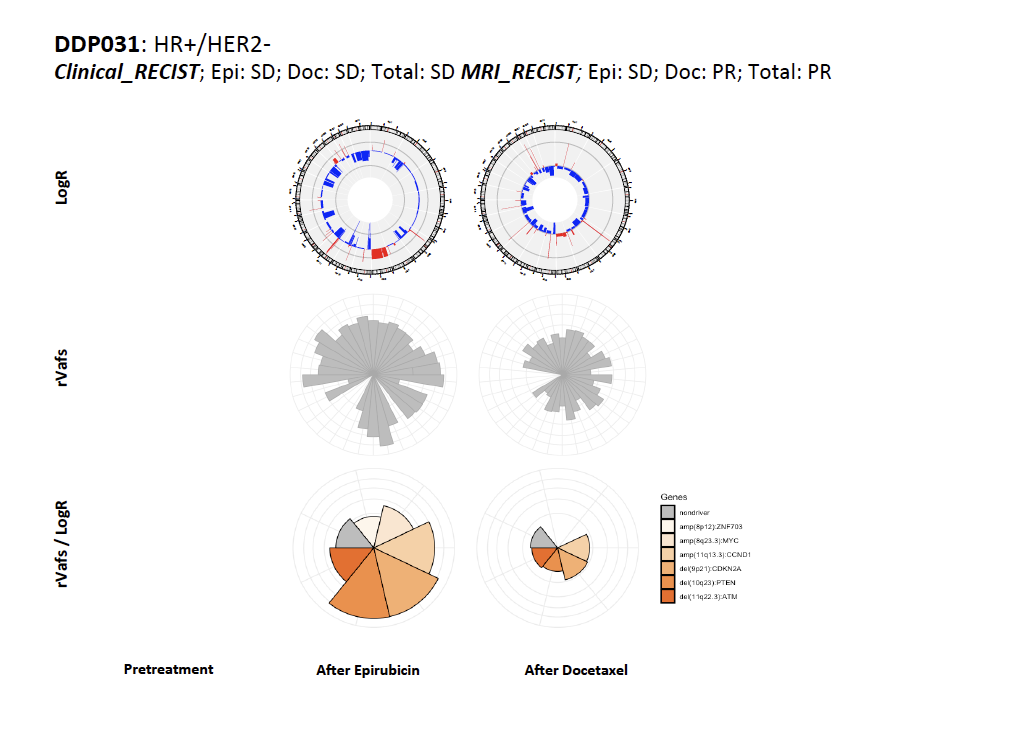

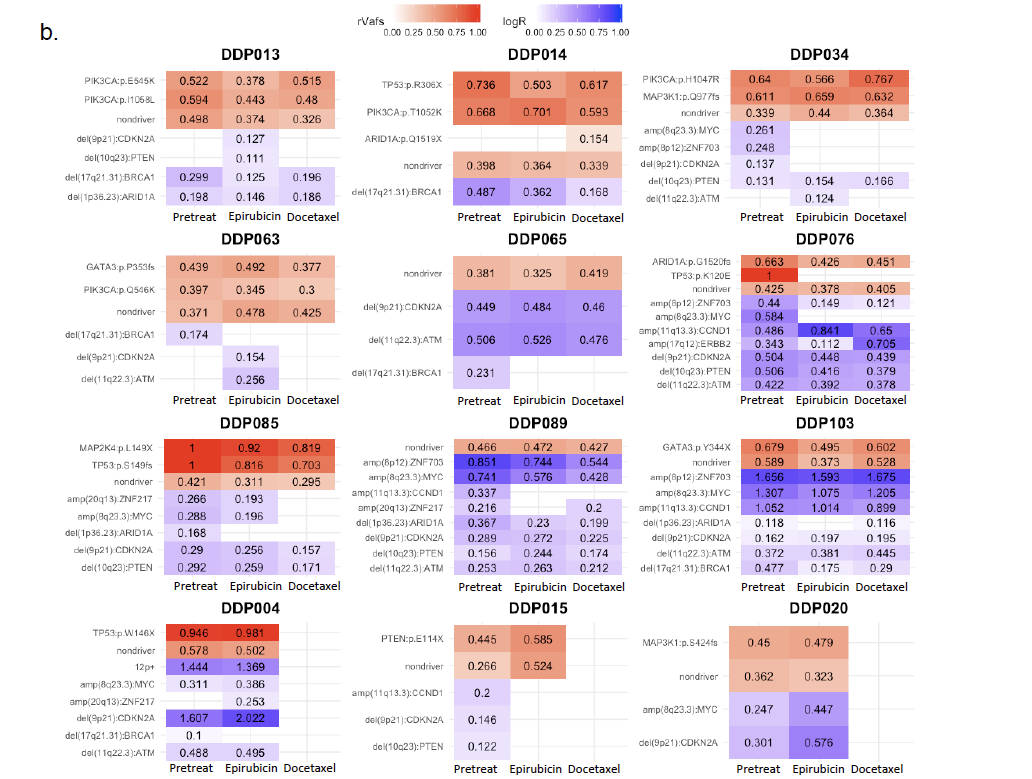
**

**
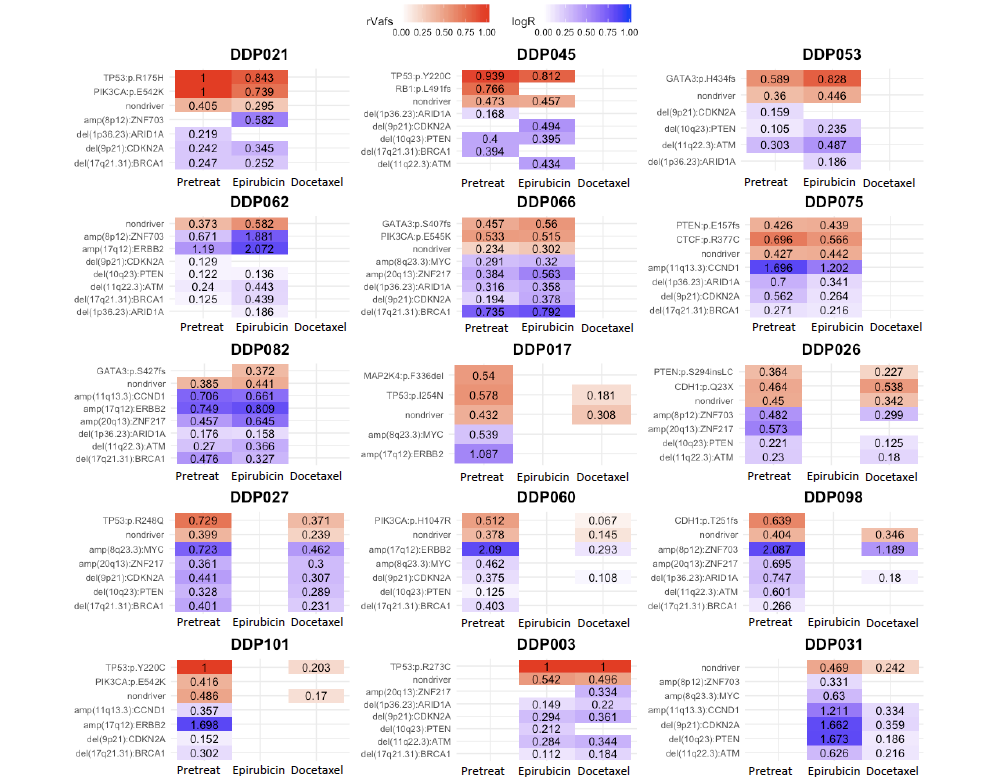
**

**Fig. S10**

**a)** Circos and coxcomp plots for all patients with sufficient number of mutations and tumor cell fraction ≥ 20% (n=27), from pretreatment, post-epirubicin and post-docetaxel biopsies. Top circos plots show copy number alteration (CNA) estimates (logR) for the three timepoints (pretreatment, post-epirubicin and post-docetaxel). Red color indicates amplifications and blue color indicates deletions. Middle coxcomb plots present somatic mutations for the three time points. Relative variant allele frequencies (rVAFs) are presented by lateral extension of an outlined wedge. Grey wedges represent “passenger mutations” while colored wedges “driver mutations”. Bottom coxcomb plots represent somatic mutations as well as driver copy number alterations. Grey wedges represent merged “passenger mutations” while colored wedges represent driver somatic mutations and CNAs.

**b)** Heatmaps for each patient with the exact rVAFs and logR for somatic mutations and CNAs, respectively. Red panel represents the rVAFs while the blue panel logR.
