# Supplementary material for: Clonal evolution in primary breast cancers under sequential epirubicin and docetaxel monotherapy
Source: Genome Med. 2022 Aug 11;14:86. doi: 10.1186/s13073-022-01090-2 (PMC9367103; doi:10.1186/s13073-022-01090-2)
Supplement: Supplementary file 4 — Additional file 4: Table S1. Baseline characteristics. Table S2a. 6 most common serious adverse event (grade 2 or more). Table S2b. 8 most common adverse event (any grade). Table S3. Response to neoadjuvant epirubicin and docetaxel in stage II-III breast cancers, and outcome at surgery. Table S4. Treatment response. Table S5a. Pretreatment mutations and prediction to epirubicin treatment. Table S5b. Pretreatment mutations and prediction to docetaxel treatment. Table S6. Mutations in pretreatment biopsies and prediction to epirubicin treatment. Table S7. Mutations in post-epirubicin biopsies and prediction to docetaxel treatment. [file 13073_2022_1090_MOESM4_ESM.docx]

**Table S1.**  **Baseline** **characteristics**

| Parameter | Overall n = 109 (%) |
| --- | --- |
| Accrual period, mo.yr | 11.2007 - 02.2016 |
| Median age (range) | 47.1 (23.5 - 70.6) |
| Tumor diameter, mm (clinical)  Range  Median  Mean | 12-270  57  59 |
| T stage (clinical)  T2^1^  T3  T4 | 18 (17%)  75 (69%)  16 (15%) |
| N stage (clinical)  N0  N1  N2  N3 | 60 (55%)  29 (27%)  13 (12%)  7 (6%) |
| M stage (radiological)  M0  M1 | 108 (99%)  1 (1%) |
| Histology  Infiltrating ductal carcinoma  Infiltrating lobular carcinoma  Infiltrating carcinoma  Undifferentiated carcinoma  Mixed histology^2^ | 80 (73%)  17 (16%)  1 (1%)  6 (6%)  5 (5%) |
| Tumor grade^3^  1  2  3  not assessed | 5 (5%)  25 (23%)  36 (33%)  43 (39%) |
| Receptor status  ER  Negative^4^  Positive < 50%  Positive > 50%  PgR  Negative^5^  Positive < 50%  Positive > 50%  HR (ER + PgR)  Negative^4+5^  Positive < 50%  Positive > 50%  HER2 ^6+7^  Negative  Positive | 32 (29%)  6 (6%)  71 (65%)  50 (46%)  20 (18%)  39 (36%)  32 (29%)  5 (5%)  72 (66%)  85 (78%)  24 (22%) |

1 T2 tumors only included if T >4cm. T stage and all subsequent tumor characteristics given for stage 2, 3 and 4 combined.

2 A combination of more than one histology type (DCIS components are not included here) 3 When grade is set to 1-2 or 2-3 the highest grade is chosen, "Worst case principle».

4 ER cut-off value <1 %.

5 PR cut-off value <10 %.

6 Patients with HER2 positive breast cancers were given docetaxel + trastuzumab.

7 HercepTest IHC was performed on all tumors, and HER2 in situ hybridization for tumors with staining score 2 by IHC.

**Table S2a.** **6** **most** **common** **serious** **adverse** **event** **(grade** **2** **or** **more)**

|  | **Overall n = 109 (%)** | | |
| --- | --- | --- | --- |
| **Parameter** | **Epirubicin** | **Taxotere** | **Total** |
| **Hand foot syndrome** | 0 (0%) | 30 (27.5%) | 30 (27.5%) |
| **Paraesthesia** | 0 (0%) | 13 (11.9%) | 13 (11.9%) |
| **Infection** | 2 (1.8%) | 9 (8.3%) | 11 (10.1%) |
| **Mucositis** | 1 (0.9%) | 3 (2.8%) | 4 (3.7%) |
| **Diarrhea** | 1 (0.9%) | 3 (2.8%) | 4 (3.7%) |
| **Thrombosis** | 3 (2.8%) | 0 (0%) | 3 (2.8%) |

**Table S2b.** **8** **most** **common** **adverse** **event** **(any grade)**

|  | **Overall n = 109 (%)** | | |
| --- | --- | --- | --- |
| **Parameter** | **Epirubicin** | **Taxotere** | **Total** |
| **Hand foot syndrome** | 0 (0%) | 50 (45.9%) | 50 (45.9%) |
| **Paraesthesia** | 4 (3.7%) | 41 (37.6%) | 45 (41.3%) |
| **Infection** | 8 (7.3%) | 14 (12.8%) | 22 (20.2%) |
| **Mucositis** | 1 (0.9%) | 20 (18.3%) | 21 (19.3%) |
| **Rash** | 4 (3.7%) | 15 (13.8%) | 19 (17.4%) |
| **Nail changes** | 2 (1.8%) | 16 (14.7%) | 18 (16.5%) |
| **Dysgeusia** | 1 (0.9%) | 10 (9.2%) | 11 (10.1%) |
| **Diarrhea** | 2 (1.8%) | 8 (7.3%) | 10 (9.2%) |

**Table S3. Response to neoadjuvant epirubicin and docetaxel in stage II-III breast cancers, and outcome at surgery**

| Clinical response* | *Response epirubicin* | *Response docetaxel* | *Response total* |
| --- | --- | --- | --- |
| *CR* | 3 | 7 | 10 |
| *PR* | 42 | 25 | 69 |
| *SD* | 61 | 68 | 29 |
| *PD* | 3 | 5 | 1 |
| *Not evaluable*** |  | 4 |  |
| *Clinical ORR* | 45/109 (41%) | 32/105 (30%) | **79/109 (72%)** |
|  |  |  |  |
| MRI response* | ***Response epirubicin*** | ***Response docetaxel*** | ***Response total*** |
| *CR* | 0 | 22 | 22 |
| *PR* | 38 | 51 | 66 |
| *SD* | 62 | 21 | 8 |
| *PD* | 5 | 1 | 0 |
| *MRI not taken/not evaluable* | 4 | 14 | 13 |
| *ORR* | 38/105 (36%) | 73/95 (77%) | **88/96 (92%)** |
|  |  |  |  |
| Pathological complete response  (pCR)*** | ***Breast cancer subgroup******* |  | ***pCR rates*** |
|  | HR+/HER2- |  | 2/63 (3%) |
|  | HER2+ |  | 8/24 (33%) |
|  | TNBC |  | 6/20 (30%) |
| pCR total |  |  | **16/107 (15%)** |

*evaluation by RECIST criteria, except PD, which was defined by UICC criteria.

**not evaluable due to CR on epirubicin (n=3) or no docetaxel given (n=1)

***pCR defined as ypT0 ypN0, in situ carcinoma (is+) included

****HR+: hormone receptor positive, HER2-: HER2 normal/negative, TNBC: triple-negative breast cancer

**Table S4.** **Treatment response**

See separate file

(Additional file 5)

**Table S5a. Pretreatment mutations and prediction to epirubicin treatment**

|  | Response groups | | | | Trend analysis | |
| --- | --- | --- | --- | --- | --- | --- |
|  | **CR** | **PR** | **SD** | **PD** | **p.val** | **q.val** |
| Patients | 3 | 40 | 50 | 3 |  |  |
| Mutations |  |  |  |  |  |  |
| *TP53* | 1 | 19 | 16 | 0 | 0.09 | 0.55 |
| *PIK3CA* | 1 | 15 | 12 | 1 | 0.27 | 0.84 |
| *GATA3* | 0 | 7 | 8 | 1 | 0.60 | 1 |
| *CDH1* | 1 | 10 | 9 | 0 | 0.21 | 0.84 |
| *BRCA1* | 0 | 3 | 4 | 0 | 0.93 | 1 |
| *TBX3* | 0 | 3 | 0 | 0 | 0.11 | 0.56 |

**Table S5b. Pretreatment mutations and prediction to docetaxel treatment**

|  | Response groups | | | | Trend analysis | | |
| --- | --- | --- | --- | --- | --- | --- | --- |
|  | **CR** | **PR** | **SD** | **PD** | | **p.val** | **q.val** |
| Patients | 7 | 19 | 60 | 6 | |  |  |
| Mutations |  |  |  |  | |  |  |
| *TP53* | 3 | 8 | 23 | 1 | | 0.40 | 1 |
| *PIK3CA* | 4 | 5 | 17 | 1 | | 0.18 | 0.91 |
| *GATA3* | 1 | 2 | 11 | 2 | | 0.29 | 1 |
| *CDH1* | 2 | 2 | 12 | 2 | | 0.63 | 1 |
| *BRCA1* | 0 | 1 | 6 | 0 | | 0.55 | 1 |
| *TBX3* | 1 | 1 | 1 | 0 | | 0.08 | 0.45 |

**Table S6. Mutations in pretreatment biopsies and prediction to epirubicin treatment**

|  | Response groups | | | | Trend analysis | | |
| --- | --- | --- | --- | --- | --- | --- | --- |
|  | **CR** | **PR** | **SD** | **PD** | | **p.val** | **q.val** |
| Patients | 2 | 23 | 23 | 3 | |  |  |
| Mutations |  |  |  |  | |  |  |
| *JAK2* | 1 | 2 | 0 | 0 | | 0,02 | 0,99 |
| *TP53* | 0 | 12 | 6 | 0 | | 0,12 | 1 |
| *CDH1* | 1 | 3 | 2 | 0 | | 0,16 | 1 |
| *DDX5* | 1 | 1 | 1 | 0 | | 0,16 | 1 |
| *OBSCN* | 1 | 1 | 1 | 0 | | 0,16 | 1 |
| *SCN9A* | 1 | 1 | 1 | 0 | | 0,16 | 1 |
| *BIRC6* | 0 | 3 | 0 | 0 | | 0,16 | 1 |
| *CES1* | 0 | 3 | 0 | 0 | | 0,16 | 1 |
| *MCM3AP* | 0 | 3 | 0 | 0 | | 0,16 | 1 |
| *PRUNE2* | 0 | 3 | 0 | 0 | | 0,16 | 1 |
| *ATM* | 0 | 0 | 3 | 0 | | 0,21 | 1 |
| *DNAJB6* | 0 | 0 | 3 | 0 | | 0,21 | 1 |
| *GPR98* | 0 | 0 | 3 | 0 | | 0,21 | 1 |
| *MEGF8* | 0 | 0 | 3 | 0 | | 0,21 | 1 |
| *USH2A* | 0 | 0 | 3 | 0 | | 0,21 | 1 |
| *DND1* | 1 | 1 | 2 | 0 | | 0,38 | 1 |
| *TMEM14B* | 1 | 2 | 3 | 0 | | 0,44 | 1 |
| *TTN* | 1 | 3 | 4 | 0 | | 0,48 | 1 |
| *HMCN1* | 0 | 1 | 3 | 0 | | 0,49 | 1 |
| *TDRD6* | 1 | 0 | 2 | 0 | | 0,60 | 1 |
| *ZFHX3* | 1 | 0 | 2 | 0 | | 0,60 | 1 |
| *ACACB* | 0 | 2 | 1 | 0 | | 0,60 | 1 |
| *DNAH10* | 0 | 2 | 1 | 0 | | 0,60 | 1 |
| *KMT2C* | 0 | 2 | 1 | 0 | | 0,60 | 1 |
| *PLCB4* | 0 | 2 | 1 | 0 | | 0,60 | 1 |
| *RBMXL3* | 0 | 2 | 1 | 0 | | 0,60 | 1 |
| *RETSAT* | 0 | 2 | 1 | 0 | | 0,60 | 1 |
| *RNF213* | 0 | 2 | 1 | 0 | | 0,60 | 1 |
| *WHSC1L1* | 0 | 2 | 1 | 0 | | 0,60 | 1 |
| *ANK2* | 0 | 1 | 2 | 0 | | 0,71 | 1 |
| *ARID1A* | 0 | 1 | 2 | 0 | | 0,71 | 1 |
| *C10orf71* | 0 | 1 | 2 | 0 | | 0,71 | 1 |
| *FAM83B* | 0 | 1 | 2 | 0 | | 0,71 | 1 |
| *GABRA4* | 0 | 1 | 2 | 0 | | 0,71 | 1 |
| *MALRD1* | 0 | 1 | 2 | 0 | | 0,71 | 1 |
| *PDE3A* | 0 | 1 | 2 | 0 | | 0,71 | 1 |
| *PDE8A* | 0 | 1 | 2 | 0 | | 0,71 | 1 |
| *SIGLEC10* | 0 | 1 | 2 | 0 | | 0,71 | 1 |
| *SVIL* | 0 | 1 | 2 | 0 | | 0,71 | 1 |
| *WDFY4* | 0 | 1 | 2 | 0 | | 0,71 | 1 |
| *ZFHX4* | 0 | 1 | 2 | 0 | | 0,71 | 1 |
| *FLG* | 0 | 2 | 3 | 0 | | 0,80 | 1 |
| *GATA3* | 0 | 3 | 1 | 1 | | 0,80 | 1 |
| *MUC16* | 0 | 2 | 3 | 0 | | 0,80 | 1 |
| *PIK3CA* | 1 | 6 | 6 | 1 | | 0,80 | 1 |
| *PTEN* | 0 | 3 | 3 | 0 | | 0,91 | 1 |
| *DNAH8* | 0 | 2 | 2 | 0 | | 0,93 | 1 |

**Table S7. Mutations in post-epirubicin biopsies and prediction to docetaxel treatment**

|  | Response groups | | | | Trend analysis | | |
| --- | --- | --- | --- | --- | --- | --- | --- |
|  | **CR** | **PR** | **SD** | **PD** | | **p.val** | **q.val** |
| Patients | 2 | 12 | 29 | 3 | |  |  |
| Mutations |  |  |  |  | |  |  |
| *ANK2* | 0 | 0 | 3 | 0 | | 0.43 | 1 |
| *ARID1A* | 0 | 3 | 10 | 2 | | 0.12 | 1 |
| *ATM* | 2 | 10 | 15 | 3 | | 0.23 | 1 |
| *BRCA1* | 2 | 2 | 13 | 1 | | 0.97 | 1 |
| *CCND1* | 1 | 1 | 5 | 1 | | 0.88 | 1 |
| *CDH1* | 1 | 1 | 0 | 1 | | 0.29 | 1 |
| *CDKN2A* | 2 | 5 | 19 | 2 | | 0.67 | 1 |
| *CSMD3* | 1 | 0 | 1 | 1 | | 0.89 | 1 |
| *DNAH10* | 0 | 1 | 2 | 0 | | 0.89 | 1 |
| *DND1* | 0 | 2 | 2 | 0 | | 0.48 | 1 |
| *ERBB2* | 1 | 2 | 7 | 1 | | 0.95 | 1 |
| *FLG* | 0 | 1 | 3 | 0 | | 0.92 | 1 |
| *GATA3* | 1 | 1 | 3 | 0 | | 0.25 | 1 |
| *HMCN1* | 0 | 1 | 2 | 0 | | 0.89 | 1 |
| *KMT2C* | 0 | 1 | 2 | 0 | | 0.89 | 1 |
| *MAP3K1* | 0 | 0 | 2 | 1 | | 0.09 | 1 |
| *MUC16* | 0 | 1 | 4 | 1 | | 0.25 | 1 |
| *MYC* | 1 | 3 | 9 | 1 | | 0.98 | 1 |
| *PIK3CA* | 1 | 2 | 9 | 0 | | 0.75 | 1 |
| *PTEN* | 0 | 6 | 10 | 2 | | 0.61 | 1 |
| *RETSAT* | 0 | 2 | 1 | 1 | | 0.92 | 1 |
| *SPTBN5* | 0 | 0 | 3 | 0 | | 0.43 | 1 |
| *TBX22* | 0 | 0 | 2 | 1 | | 0.09 | 1 |
| *TP53* | 1 | 3 | 8 | 1 | | 0.87 | 1 |
| *TTN* | 0 | 1 | 5 | 1 | | 0.21 | 1 |
| *ZNF217* | 0 | 0 | 6 | 1 | | 0.06 | 1 |
| *ZNF703* | 1 | 2 | 3 | 2 | | 0.88 | 1 |
